# Supplementary material for: RNase III CLASH in MRSA uncovers sRNA regulatory networks coupling metabolism to toxin expression
Source: Nat Commun. 2022 Jun 22;13:3560. doi: 10.1038/s41467-022-31173-y (PMC9217828; doi:10.1038/s41467-022-31173-y)
Supplement: Supplementary file 1 — Supplementary Information [file 41467_2022_31173_MOESM1_ESM.pdf]

Supplementary Information

RNase III CLASH in MRSA uncovers sRNA regulatory networks coupling metabolism to toxin expression

Stuart W. McKellar[1], Ivayla Ivanova[1], Pedro Arede[1], Rachel L. Zapf[2], Noémie Mercier[3], Liang-Cui Chu[1], Daniel G. Mediati[4], Amy C. Pickering[5], Paul Briaud[2], Robert Foster[6], Grzegorz Kudla[6], J. Ross Fitzgerald[5], Isabelle Caldelari[3], Ronan K. Carroll[2,7], Jai J. Tree[4] and Sander Granneman[1]*

Affiliations:

[1]Centre for Synthetic and Systems Biology, University of Edinburgh, Edinburgh EH9 3BF, UK.

[2]Department of Biological Sciences, Ohio University, Athens, OH 45701, USA.

[3]Université de Strasbourg, CNRS, Architecture et Réactivité de l'ARN, UPR9002, F-67000-Strasbourg, France.

[4]School of Biotechnology and Biomolecular Sciences, University of New South Wales, Sydney 2052, NSW, Australia.

[5]The Roslin Institute and Edinburgh Infectious Diseases, University of Edinburgh, Easter Bush Campus, Edinburgh, Scotland, United Kingdom.

[6]MRC Human Genetics Unit, University of Edinburgh, Edinburgh EH4 2XU, United Kingdom.

[7]The Infectious and Tropical Disease Institute, Ohio University, Athens, OH 45701, USA.

*To whom correspondence should be addressed:
Sander Granneman
e-mail: Sander.Granneman@ed.ac.uk
Tel: +44 131 6519082

Content:

**Supplementary Data:**	**2-5**
**Supplementary Figures:**	**6-29**
**Supplementary References:**	**30-32**

Supplementary Data

Optimizing the CLASH protocol for *Staphylococcus aureus*

The CLASH protocol (Supplementary Fig. 5a) has been successfully performed in *Saccharomyces cerevisiae*, *Escherichia coli* and cultured human cells[1–4]. Unfortunately, the cell disruption and immunoprecipitation conditions used for CLASH in these species resulted in poor cell lysis and significant RNA degradation in *S. aureus.* As a result, we further optimised the protocol. Briefly, cells were lysed in the presence of lysostaphin to break down the peptidoglycan cell wall. DNase RQ1 was included during the lysis to degrade the extracellular DNA and reduce viscosity of the lysate. The addition of Triton X-100, Superase-In and EDTA after cell lysis substantially increased the recovery of cross-linked RNPs. Additionally, we also reduced the primary, anti-FLAG capture time from overnight to just 2 hours and compensated by increasing the quantity of anti-FLAG beads. Collectively, these optimisations enhanced the solubility of the bait protein and reduced RNA degradation through both inactivating and reducing the contact time with endogenous RNases. To quantify the recovery of UV cross-linked RNA, we radiolabelled the RNA cross-linked to the bait protein after the purification steps and resolved the RNP complex by SDS-PAGE. The effectiveness of RNA capture was measured through autoradiography. Using the standard CLASH protocol, very long (>50 hours) exposure times were needed to detect the radiolabelled RNA cross-linked to purified RNase III, indicating a paucity of captured RNA (Supplementary Fig. 5b). The optimized protocol reduced exposure times to roughly 3 hours, an almost 19-fold improvement. We also examined RNase III cross-linking to its target RNAs during RPMI and LPM stress. We observed a strong increase in the degree of RNA capture in these stresses in comparison to TSB, suggesting that RNase III plays an active role in the adaptation process (Supplementary Fig. 5c). Encouragingly, no radioactive signal was observed for a parental, untagged control, indicating that the cross-linking is specific to RNase III. Overall, we anticipate that our modifications will facilitate the application of CLASH to Gram-positive bacteria in general.

Known interactions identified by CLASH

Supplementary Fig. 8 shows the structures of RNA-RNA interactions detected through CLASH that have been previously identified. These structures were obtained by extracting the corresponding reads found through CLASH and folding them *in silico* through RNADuplex.

RsaA targets

RsaA contains a distinctive 3' UCCC nucleotide tract; a motif which is utilised by other sRNAs, e.g., RNAIII and RsaE, to bind to the Shine Dalgarno (SD) sequence of targets. RsaA

is known to repress the translation of a global transcription factor, *mgrA*, in order to stimulate biofilm formation and inhibit capsule synthesis[5]. We identified this interaction in both USA300 and JKD6009, primarily in RPMI but also in TSB. Previous structural work has elucidated that RsaA can interact with two regions of *mgrA*. The first of these involves the 3' C-rich motif of RsaA binding to the SD sequence of *mgrA*, creating a target site for RNase III. CLASH of RNase III perfectly recapitulated this interaction and all the involved nucleotides. The second interaction site involves the fourth hairpin loop of *mgrA*, around 200 nucleotides within the coding sequence, but this is not thought to recruit RNase III[5]. We did not recover this site using RNase III CLASH, and as such, our results confirm that only the interaction around the start codon is an RNase III target.

Targetome-capture of RsaA via 'MS2 affinity purification coupled to RNA sequencing' (MAPS) revealed that RsaA can bind *HG001_01977*[6] (annotated here as *JKD6008_01954* and *SAUSA300_1921*), a protein of unknown function. We identified this interaction in both JKD6008 in TSB and in USA300 in RPMI. Previous work found that mutating the 3' C-rich motif of RsaA had no effect on binding to *HG001_01977*, we found the 5' region of RsaA (as is also known to bind hairpin 4 of *mgrA*) as binding to HG001_01977. This is further evidence for a second seed sequence in RsaA at its 5' end.

RsaE targets

RsaE is an sRNA known to be involved in the citric acid cycle[7], amino acid catabolism[8], folate metabolism[9] and oligopeptide transport[7]. It has been found to bind *oppB*, an oligopeptide transporter from the *opp-3* operon, at the SD sequence to prevent ribosomal binding. CLASH perfectly recovered this interaction, showing that this RNA-RNA duplex also acts to recruit RNase III and so maybe also control RNA stability[7,9]. MAPS capture of RsaE also revealed *purH* as a target, although no detailed structural work was performed[8]. We captured this interaction in JKD6009 in TSB, although it did not utilise one of RsaE's canonical UCCC motifs nor the 5' UTR of *purH*.

SprX - *spoVG*

The translational efficiency of a protein involved in antibiotic resistance and capsule formation, *spoVG*, is known to be negatively regulated by SprX[10]. Again, SprX is thought to utilise a UCCC motif to interact with the SD sequence of *spoVG*[10]. RNase III CLASH perfectly recapitulated this interaction. Overexpression of SprX also led to a decrease in the stability of *spoVG*[10], and so our CLASH data puts RNase III as a contributor towards this regulation. This interaction was found both in the JKD6009 and USA300 CLASH data and was highly reproducible.

Toxin-antitoxin systems

Several type I toxin-antitoxin systems are well characterised in *S. aureus*, where an unstable antisense RNA represses the translation of a more stable, toxic mRNA. The most well characterised of these is between *sprA1*, encoding for the toxic PepA1 peptide, and $SprA1_{AS}$[11]. Here, the antisense $SprA1_{AS}$ represses the translation of SprA1 by covering the ribosomal binding site[11]. We consistently identified this interaction in both tested strains and in all tested conditions, and *in silico* folding of the CLASH reads revealed $SprA1_{AS}$ binding to the 5' UTR and start codon, as expected.

Previously linked pathways are directly connected by CLASH

1. RNAIII – *saeR*

The *sae* ('*S. aureus* exoprotein expression') locus encodes for a two-component system that is critical for production of virulence-related transcripts. SaeS, a sensor histidine kinase, autophosphorylates in response to external signals such as human neutrophil peptides[12]. Phosphorylated SaeS can then activate SaeR, a transcription factor that stimulates the transcription of haemolyins (including αPSMs), leukocidins, superantigens, surface proteins and proteases[13].

The *sae* system has been linked to the *agr* quorum sensing network. Firstly, these two networks have similar functionalities. The effector molecule of *agr*, RNAIII, is a major post-transcriptional regulator that also promotes the production of virulence-related transcripts, and AgrA, the transcription factor component of the *agr* system, can induce the transcription of cytolytic PSMs such as αPSMs[14]. Additionally, deletion of the *agr* locus strongly diminishes production of the *sae* operonic mRNA[15]. We observed direct interactions between RNAIII and *saeR* and so the *agr* system may also regulate *sae* at the post-transcriptional level by regulating *saeR* translation and/or RNA stability via RNAIII.

2. *The agr* and *dlt* operons

The *agr* quorum sensing network has been linked to the *dlt* operon, which is involved in D-alanine modification of teichoic acids to confer resistance to human antimicrobial peptides[16]. Deletion of the *agr* locus led to an increase in *dlt* expression in stationary phase (when *agr* is expressed most strongly). Although a role for *agrA*, the transcriptional regulator of the *agr* locus, was proposed, here we observed direct interactions between RNAIII and *dltA* and *dltD*. Thus, these two pathways may also be connected as the post-transcriptional level.

RNAIII and RsaA

One of the most abundant interactions that we identified in the JKD6009 CLASH data was the interaction between RNAIII and RsaA (Supplementary Fig. 9a). This interaction was almost exclusively detected in the JKD6009 CLASH data (Supplementary Fig. 9b), implying it is strain specific or regulated differently in the strains tested. The idea that RsaA is connected to the *agr* pathway, and therefore RNAIII, has been previously suggested: RsaA is positively regulated by σ^B and strains that have a fully functional σ^B also express lower levels of RNAIII[17]. Additionally, MAPS-capture of RsaA identified RNAIII as a putative binding partner, although this was suggested as being due to indirect co-purification with *mgrA*[6].

We firstly recapitulated this interaction using EMSAs. *In silico* folding of the CLASH reads revealed that RNAIII utilised helices 9, 10 and 11 to interact with RsaA (Supplementary Fig. 9c; "RNAIII fragment 1"). Because the interaction between RsaA and RNAIII involved many base-pairs (Supplementary Fig. 9a), as a control we *in vitro* transcribed an RNAIII fragment that had a similar *in silico* base-pairing potential with RsaA but was not recovered in our chimeras ("RNA fragment 2"; Supplementary Fig. 9c). The EMSA revealed that only RNAIII fragment 1 could form a stable duplex with RsaA, with no significant binding observed between RNAIII fragment 2 and RsaA (Supplementary Fig. 9d). As such, we conclude that these two sRNAs interact *in vivo* and this interaction can be recapitulated specifically *in vitro*.

To determine the biological significance of this interaction, we firstly examined if deletion of either RsaA or RNAIII induced stability changes in the partner RNA. Although we found that deletion of RNAIII resulted in reduced levels of the unprocessed RsaA transcript, the levels of the processed RsaA were similar to the WT. Similarly, deletion of RsaA had no significant effect on RNAIII levels (Supplementary Fig. 9e). We then examined if pulse-overexpression of either RsaA or RNAIII induced stability changes in the partner. Each sRNA was put under the control of an anhydrotetracycline promoter[18] and induced for 5 and 10 minutes at OD_{600} ~3, and expression was compared to a strain carrying an empty plasmid control. Again, no significant expression changes were observed (Supplementary Fig. 9f). Interestingly, we found that the levels of RNAIII were rapidly and strongly reduced following the shift to RPMI (Supplementary Fig. 9g, upper panel). This led us to hypothesise that RsaA may bind to RNAIII during stress, marking RNAIII for degradation. As such, we compared the stability of RNAIII following the shift to RPMI in both USA300 WT and a Δ*rsaA* strain. However, no differences were observed, indicating the RsaA does not play a role in regulating RNAIII stability under the tested conditions. Overall, we are unsure of the directionality of this sRNA-sRNA interaction or its functional outcome.

Supplementary Figures and Figure legends

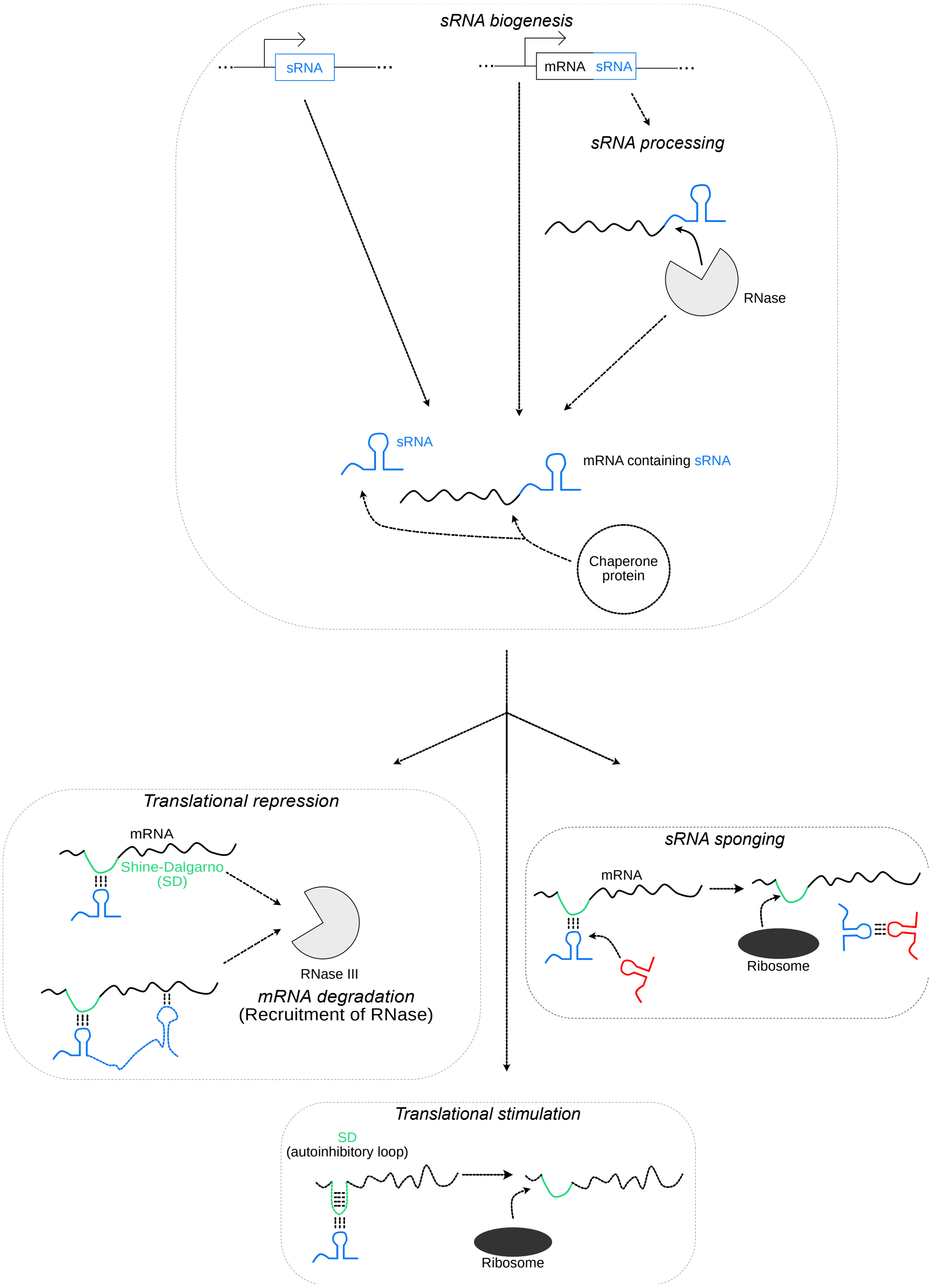

Supplementary Figure 1. sRNA biogenesis and functionality.

sRNAs can stimulate or repress mRNA translation and antagonise the activity of other sRNAs (‘sponging’).

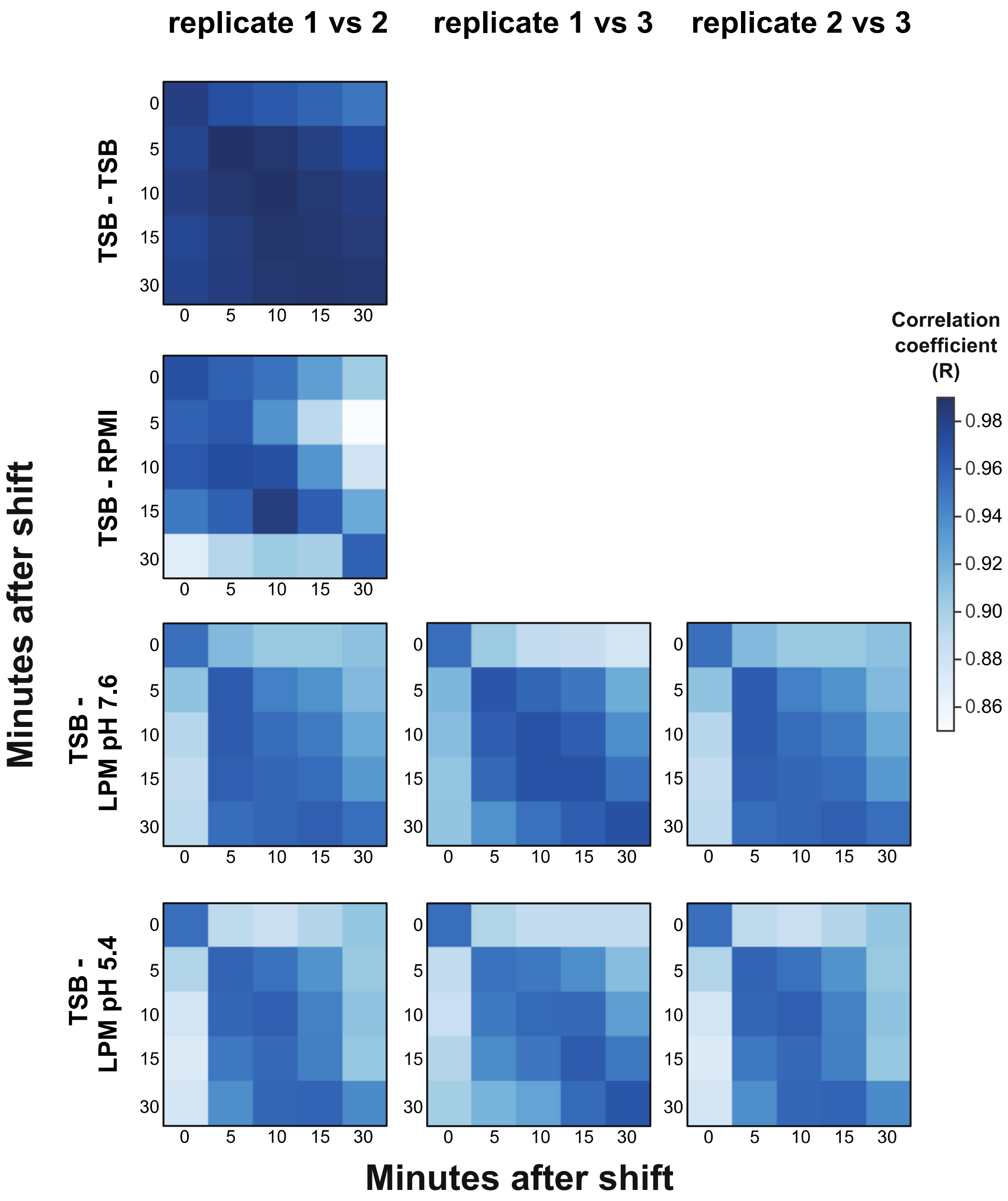

Supplementary Figure 2. Correlation of RNA-seq replicates.

The number of reads for all transcripts in common between replicates were TPM (Transcripts Per Million) and log2-normalised and Pearson correlation coefficients (R) were calculated by comparing each individual timepoint. For TSB-TSB and TSB-RPMI, two independent biological replicates were used. For TSB-LPM pH 5.4 and TSB-LPM pH 7.6, three independent biological replicates were used.

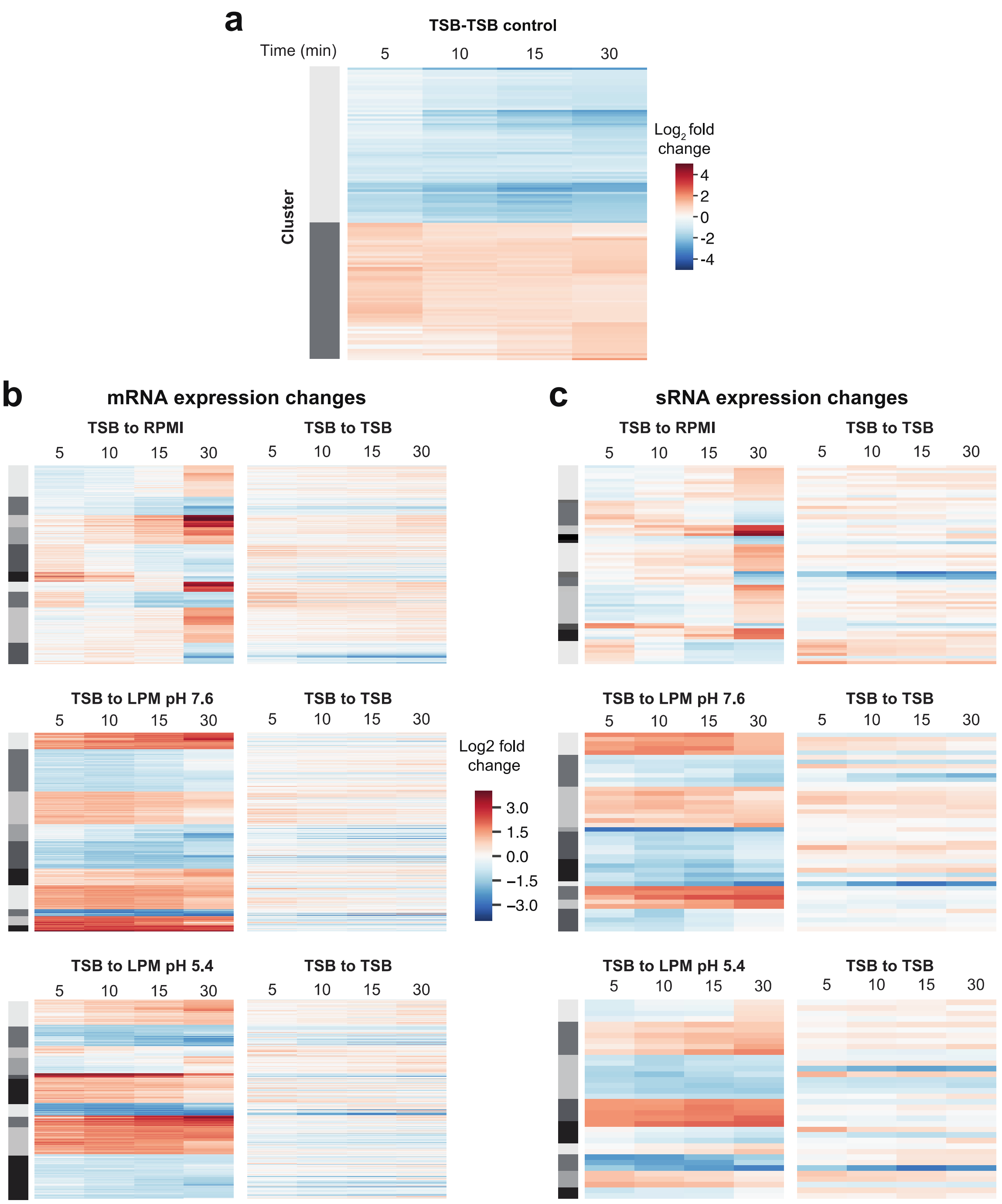

Supplementary Figure 3. Transcript expression after shift to TSB, RPMI, LPM pH 5 and LPM pH 7.

(a) Expression of all significantly changed transcripts (t30 vs t0) in the TSB-shifted control. Time indicates time (minutes) after the shift. Cluster indicates a group of genes that shows similar gene expression behaviour. Log2 fold-change indicate log2-fold normalised Transcripts Per Million (TPM) normalised read counts.

(b) Left: expression of all significantly changed mRNAs (t30 vs t0) in RPMI, LPM pH 5 and LPM pH 7. Right: TSB expression of the transcripts shown on the left, in the same order.

(c) As in (b) but for all sRNAs.

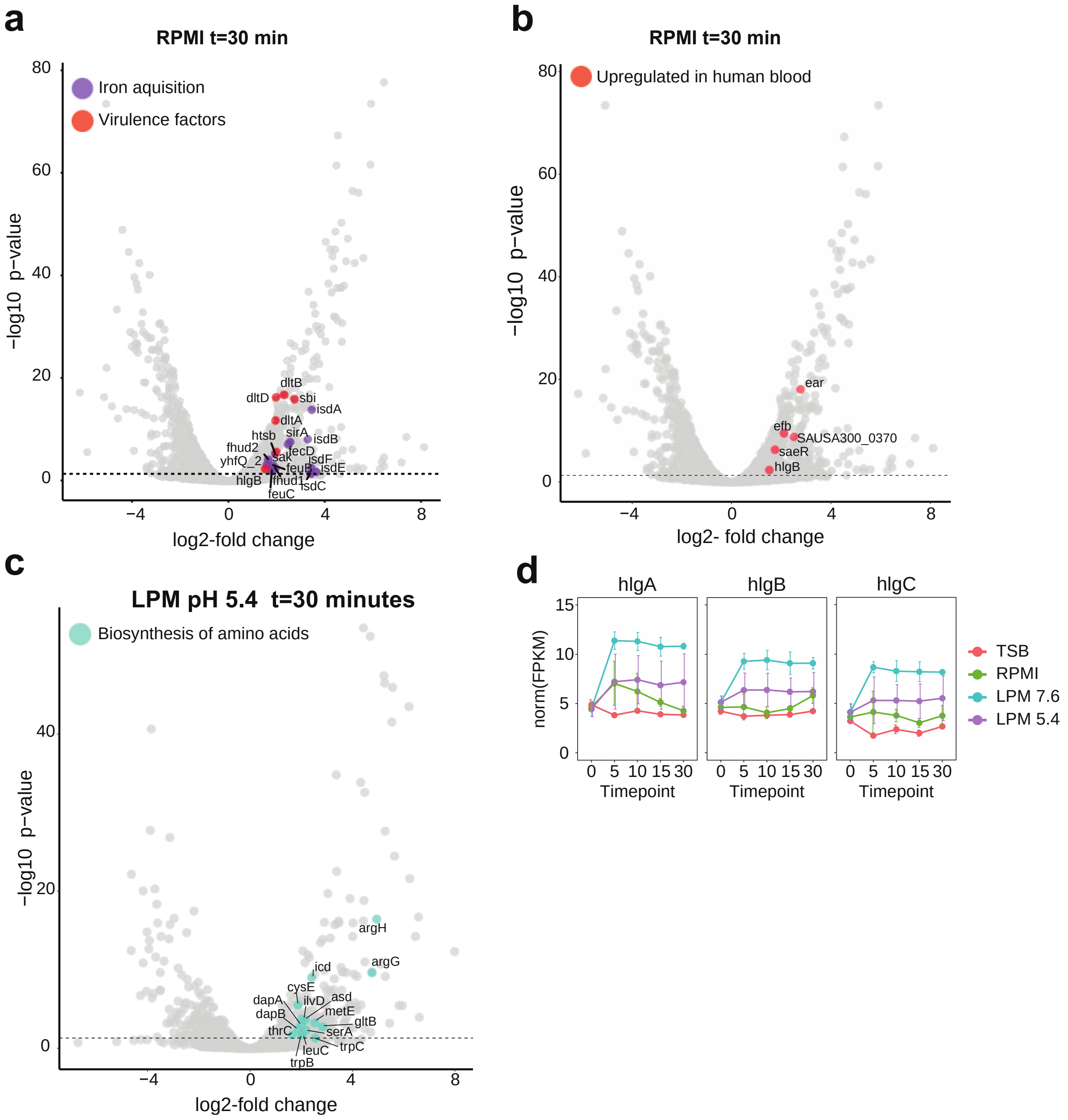

Supplementary Figure 4. Gene expression dynamics after the shift to RPMI and LPM.

(a) Volcano plot showing the upregulation of genes involved in virulence and iron-acquisition, many of which have also been demonstrated to be upregulated during growth in human plasma[19]. Fold-changes and p-values were calculated using DESeq2[20].

(b) Volcano plot showing genes upregulated in RPMI in this study that were previously observed to be upregulated in human blood[21].

(c) Volcano plot showing upregulation of genes involved in amino acid biosynthesis after shifting to LPM pH 5.4 medium. (d) Expression of haemolysin gamma subunits after stress.

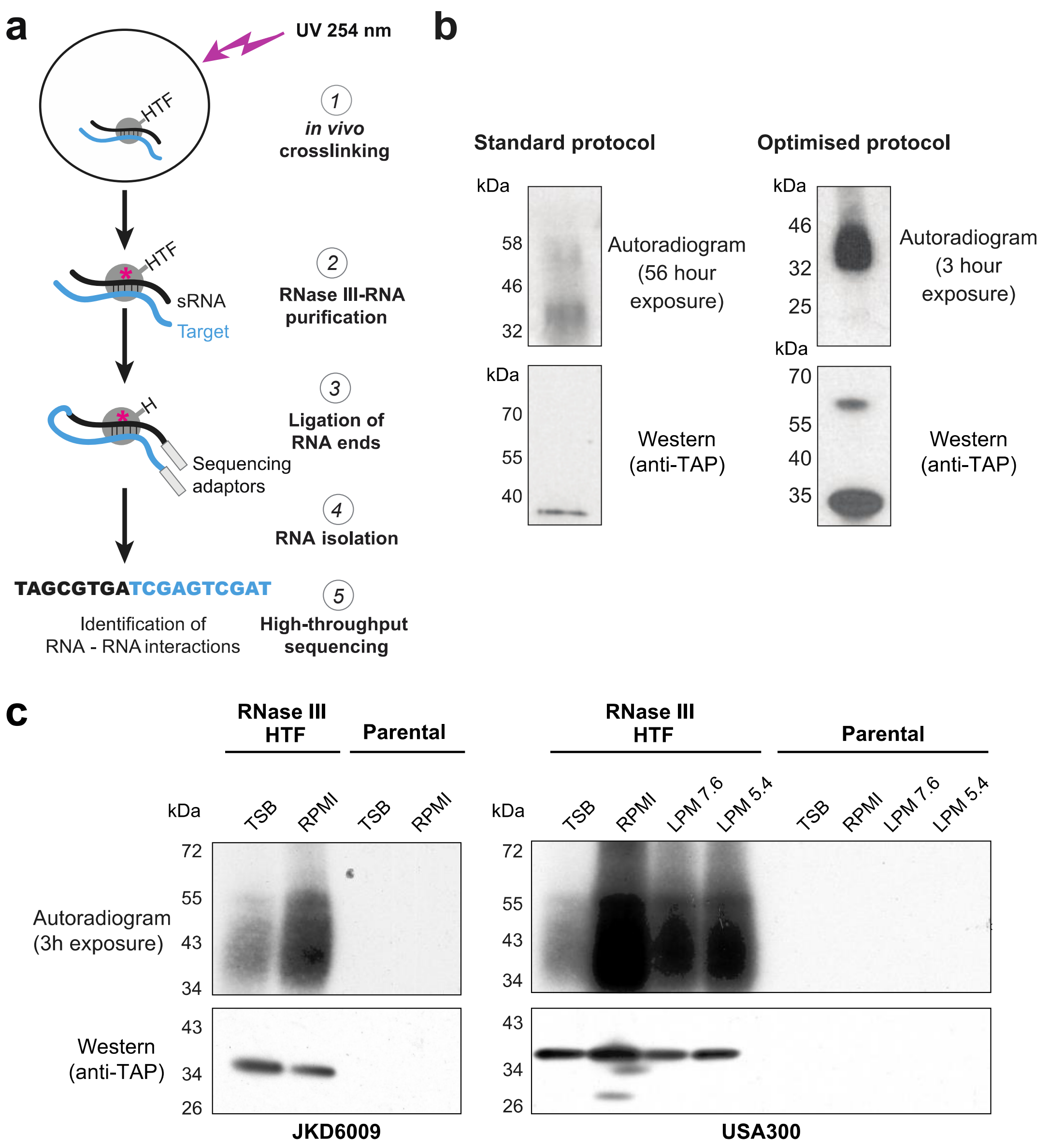

Supplementary Figure 5. Optimisation of CLASH for *S. aureus*.

(a) Schematic representation of the CLASH methodology[1]. HTF: HIS6-TEV cleavage site-3xFLAG tag.

(b) Autoradiogram showing amount of captured RNA when using the original, published CLASH protocol for *E. coli*[4] (left) and after optimisation (right) using RNase III tagged with HTF as bait. RNase III protein was detected using the anti-TAP antibody (see Methods for details) that recognizes the spacer between the HIS6 tag and the TEV protease cleavage site of the RNase III-HTF protein (bottom).

(c) Degree of cross-linked RNA capture following RNase III CLASH in TSB and after the shift to RPMI, LPM7 or LPM5 medium. The untagged parental strain was used as a negative control. RNase III was detected by Western blot using the anti-TAP antibody. Images used to generate figures (b) and (c) are provided in the Source Data file.

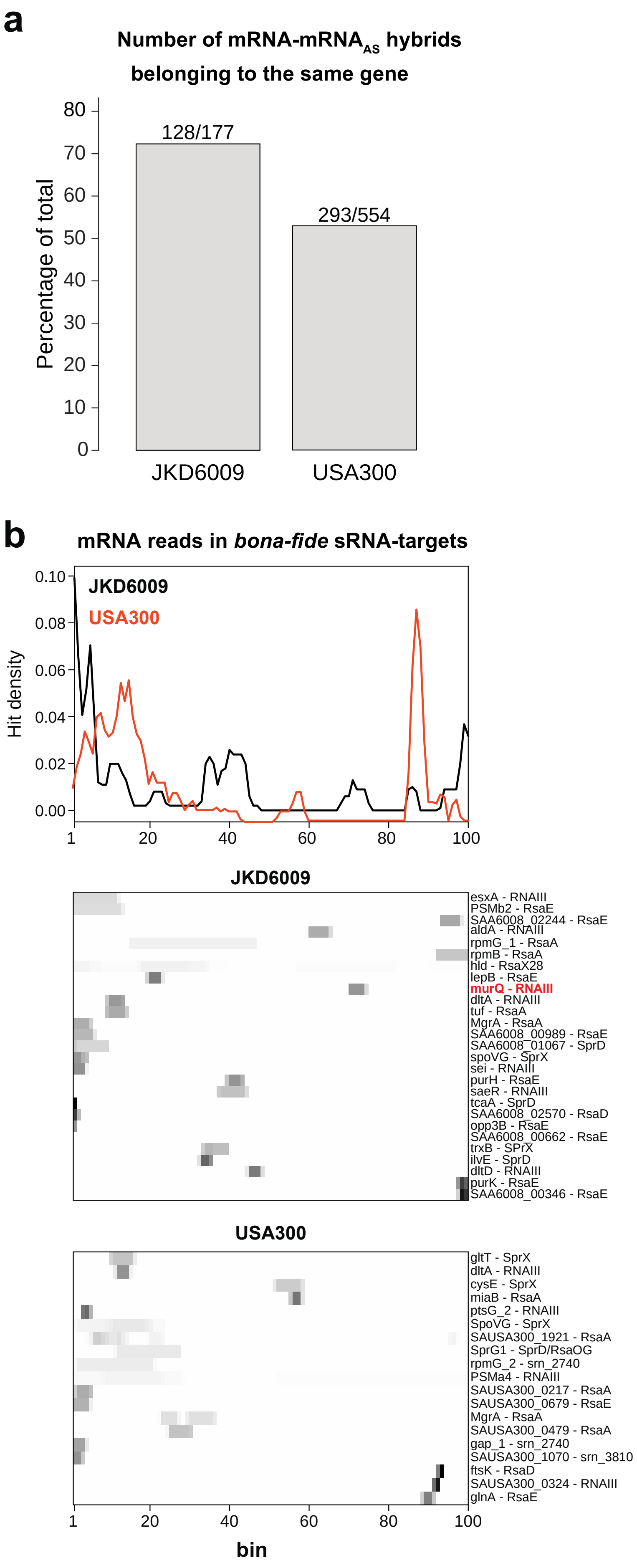
a
Number of mRNA-mRNA$_{AS}$ hybrids belonging to the same gene
Percentage of total
80
70
60
50
40
30
20
10
0
128/177
293/554
JKD6009
USA300
b
mRNA reads in *bona-fide* sRNA-targets
Hit density
0.10
0.08
0.06
0.04
0.02
0.00
JKD6009
USA300
1
20
40
60
80
100
JKD6009
esxA - RNAIII
PSMb2 - RsaE
SAA6008_02244 - RsaE
aldA - RNAIII
rpmG_1 - RsaA
rpmB - RsaA
hld - RsaX28
lepB - RsaE
murQ - RNAIII
dltA - RNAIII
tuf - RsaA
MgrA - RsaA
SAA6008_00989 - RsaE
SAA6008_01067 - SprD
spoVG - SprX
sei - RNAIII
purH - RsaE
saeR - RNAIII
tcaA - SprD
SAA6008_02570 - RsaD
opp3B - RsaE
SAA6008_00662 - RsaE
trxB - SPrX
ilvE - SprD
dltD - RNAIII
purK - RsaE
SAA6008_00346 - RsaE
USA300
gltT - SprX
dltA - RNAIII
cysE - SprX
miaB - RsaA
ptsG_2 - RNAIII
SpoVG - SprX
SAUSA300_1921 - RsaA
SprG1 - SprD/RsaOG
rpmG_2 - srn_2740
PSMa4 - RNAIII
SAUSA300_0217 - RsaA
SAUSA300_0679 - RsaE
MgrA - RsaA
SAUSA300_0479 - RsaA
gap_1 - srn_2740
SAUSA300_1070 - srn_3810
ftsK - RsaD
SAUSA300_0324 - RNAIII
glnA - RsaE
1
20
40
60
80
100
bin

Supplementary Figure 6. Sense-anti-sense mRNA interactions and distribution of mRNA fragments in protein-coding genes.

(a) Number of mRNA-mRNA$_{AS}$ fragments that contain cognate mRNAs in JKD6009 and USA300 compared to total number of mRNA-mRNA$_{AS}$ chimeras identified in the CLASH data.
(b) Top: Distribution of the mRNA fragments over coding sequences in bona fide sRNA-mRNA interactions. Target mRNAs were separated into 100 equally sized bins and reads were mapped relative to each bin. Bottom: heatmaps showing the read distribution for each individual interaction. The darker the colour, the higher number of chimeras that were detected. The interaction highlighted in red was validated in an accompanying manuscript by Mediati *et a*l.

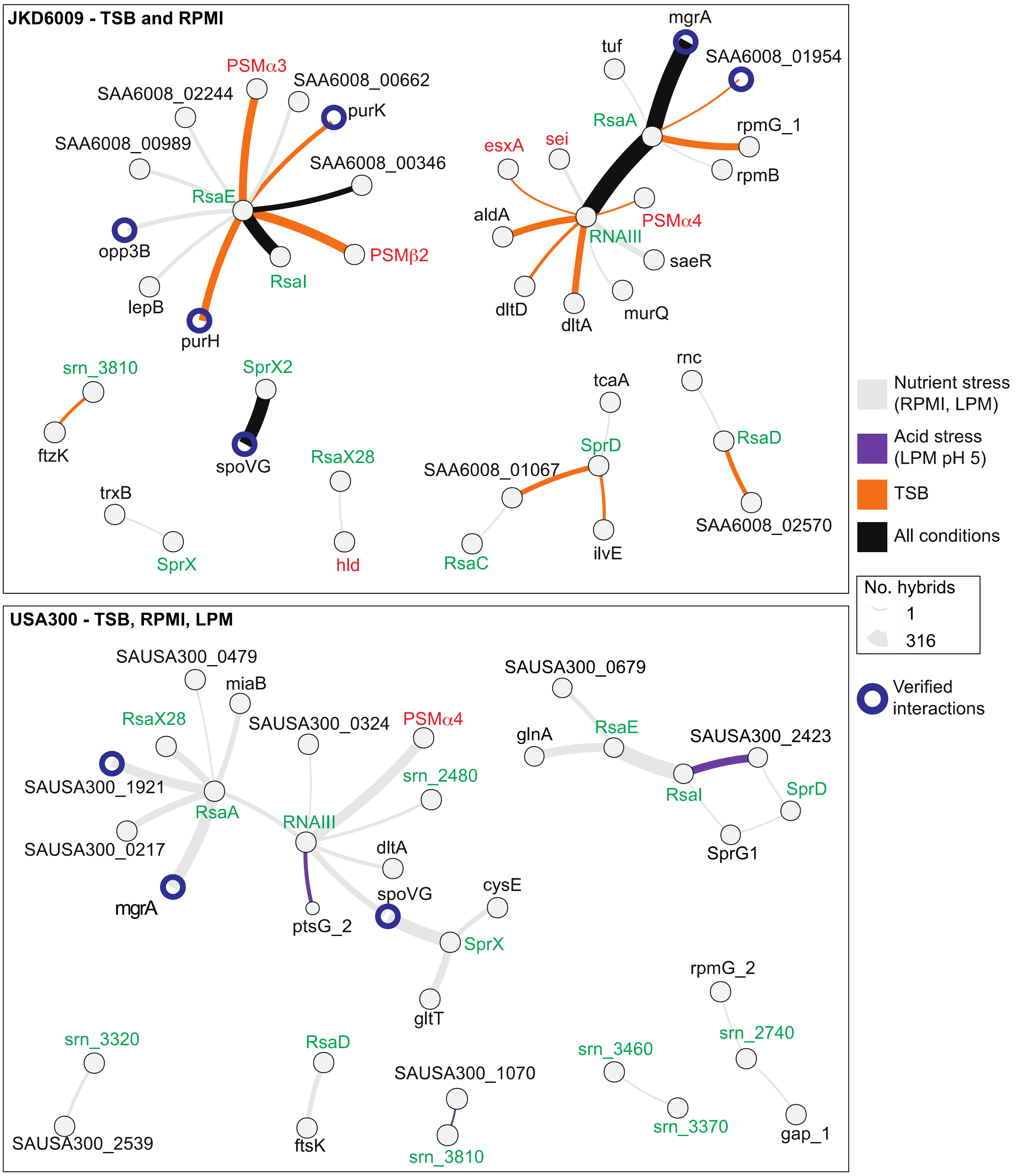

Supplementary Figure 7. CLASH identifies many novel *bona fide* sRNA-target interactions. sRNA interactome plots were generated after filtering the data for statistical significance, presence of a *bona fide* sRNA and MFE < -10 kcal/mol. sRNAs are coloured in green and toxins in red. Interactions which have already been identified are marked with a blue spoke. The thickness of the edges is proportional to the $\log_2$ hybrid count, and the colour represents the condition in which an interaction was found (minimum 1, maximum 316). Note that some previously described interactions are not shown, e.g. between $SprA1_{AS}$-*sprA1*, as the sRNAs are not trans-acting, independent transcriptional units[22].

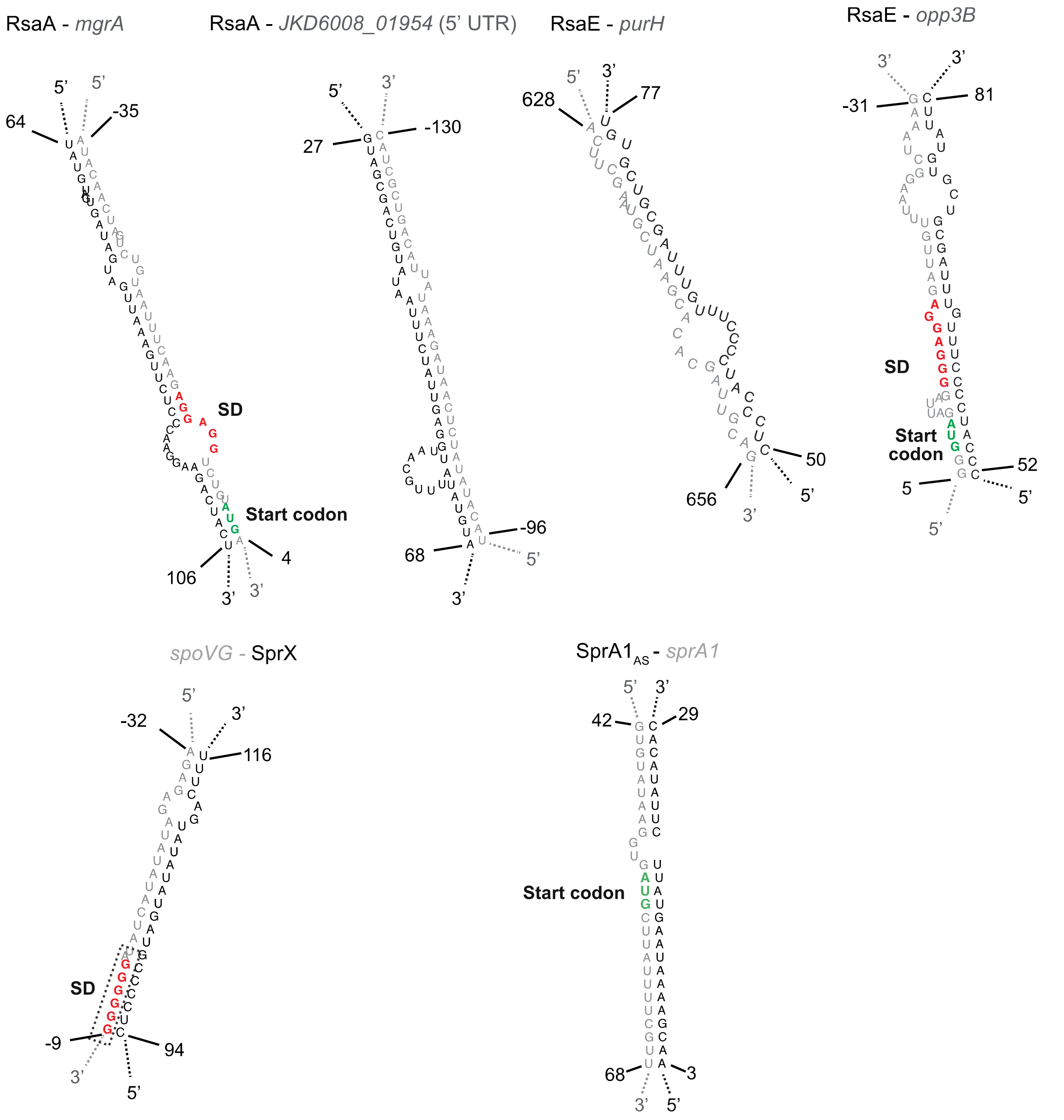

Supplementary Figure 8: Experimentally verified sRNA-mRNA interactions uncovered through CLASH. CLASH reads were folded *in silico* through RNAcofold[23] and the interacting regions extracted. The sRNAs are shown in grey and the mRNAs in black. Where appropriate, Shine Dalgarno sequences (SD; red) and translational start codons (AUG; green) of mRNAs are indicated.

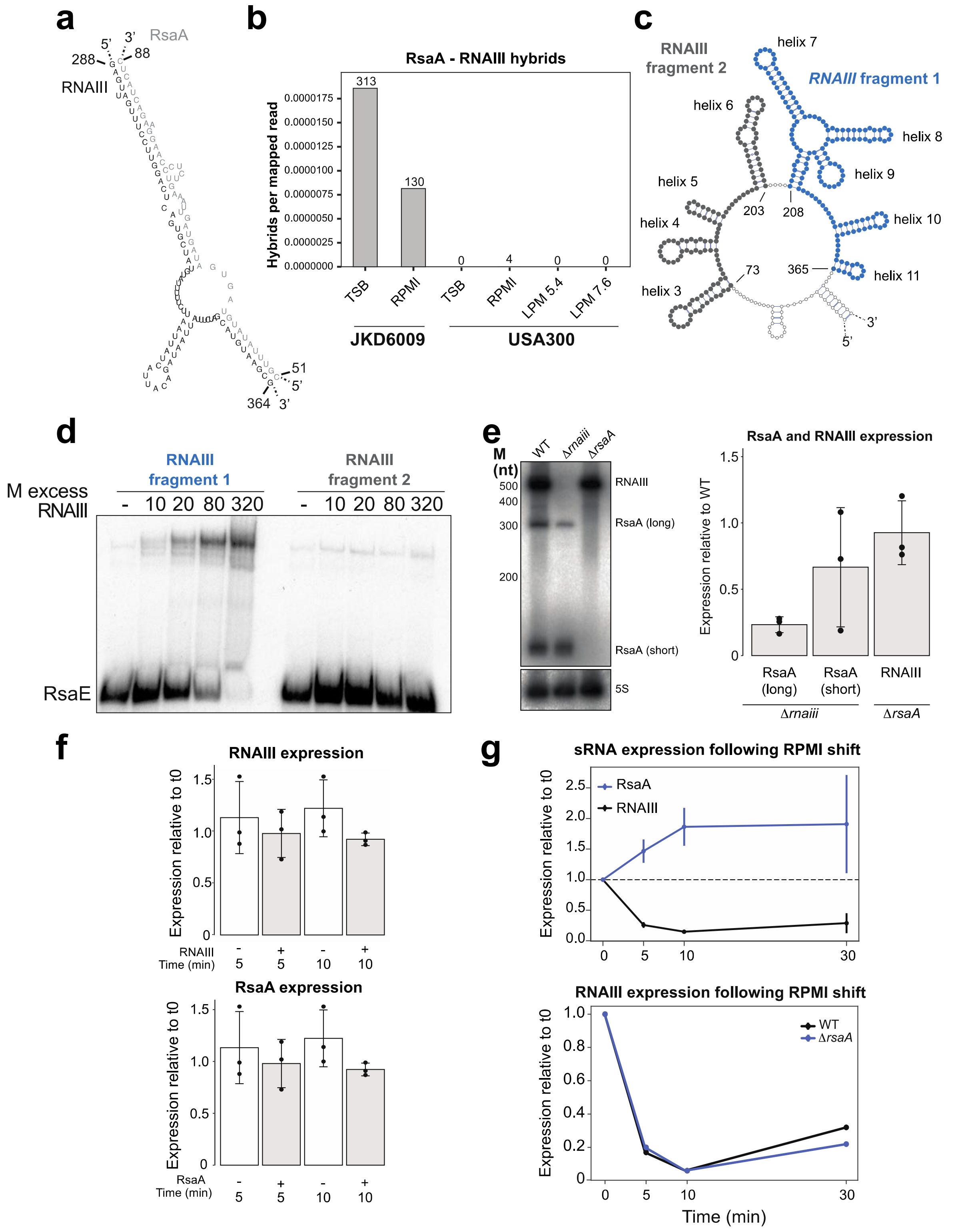

Supplementary Figure 9. RsaA and RNAIII interact *in vivo*.

(a) *In silico* predicted structure of RsaA-RNAIII as determined from the CLASH data. The CLASH reads were folded using RNAcofold[23].

(b) Overview of the number of unique RsaA-RNAIII chimeras identified in the JKD6009 and USA300 CLASH data.

(c) Secondary structure of the region containing the two RNAIII fragments 1 (blue) and 2 (grey) used for *in vitro* binding assays.

(d) EMSA between radiolabelled RsaA, RNAIII fragment 1 (containing helix 9) and RNAIII fragment 2.

(e) Northern blot analysis of RNAII and RsaA expression in USA300 RNAIII and RsaA knock-out strains, respectively (left) and quantification of the results (right). The bar chart shows average signals and standard deviations generated from Northern blot results obtained from three independent experiments.

(f) Over-expression of RNAIII or RsaA does not impact the expression levels of each other. Examination of RNAIII (top) and RsaA (bottom) expression after RsaA and RNAIII overexpression. Each sRNA was placed under a pTetO promoter[18] and induced through addition of 1 µg/mL of anhydrotetracycline at OD_{600} ~3. Samples were taken after 5 and 10 minutes and the expression of each sRNA was measured using qPCR. Data represents results from three independent biological replicates.

(g) Top line plot: expression of RsaA and RNAIII after shifting to RPMI as measured by qPCR. Data represents results from three independent biological replicates. Bottom line plot: expression of RNAIII after the shift to RPMI in WT and an Δ*rsaA* mutant.

Images and raw data used to generate figures (d-g) are provided in the Source Data file.

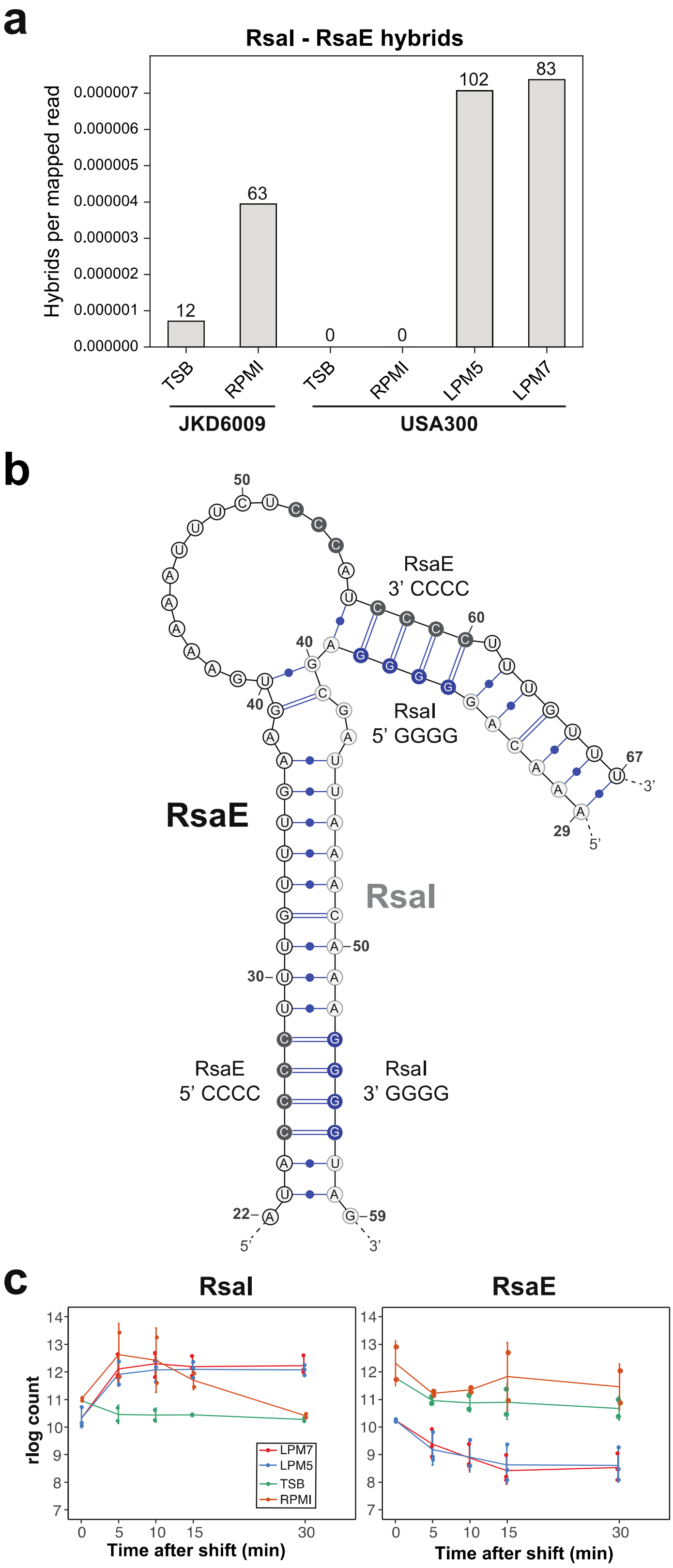

a
RsaI - RsaE hybrids
Hybrids per mapped read
0.000007
0.000006
0.000005
0.000004
0.000003
0.000002
0.000001
0.000000
12
63
0
0
102
83
TSB
RPMI
TSB
RPMI
LPM5
LPM7
JKD6009
USA300
b
RsaE
3' CCCC
RsaI
5' GGGG
RsaE
RsaI
RsaE
5' CCCC
RsaI
3' GGGG
c
RsaI
RsaE
rlog count
LPM7
LPM5
TSB
RPMI
Time after shift (min)
Time after shift (min)

Supplementary Figure 10. RsaE and RsaI interactions are abundantly detected under acid and nutrient stress conditions.

(a) Number of hybrids identified for RsaE-RsaI (left) and RsaA-RNAIII (right) across tested experimental conditions. The number above each bar is the total number of identified interactions for that condition.

(b) The RNADuplex predicted interaction between RsaI and RsaE based on the CLASH data The figure was generated using VARNA[24].

(c) Expression of RsaI and RsaE after shifting the cells to RPMI, LPM medium or back to TSB as measured by RNAseq. Samples were harvested before the shift (0) and 5, 10, 15 and 30 minutes after the shift. The results that are plotted are averages and standard deviations from three independent biological replicates for the LPMs and two independent biological replicates for RPMI and TSB data. The raw data used to generate these plots is provided in the Source Data file.

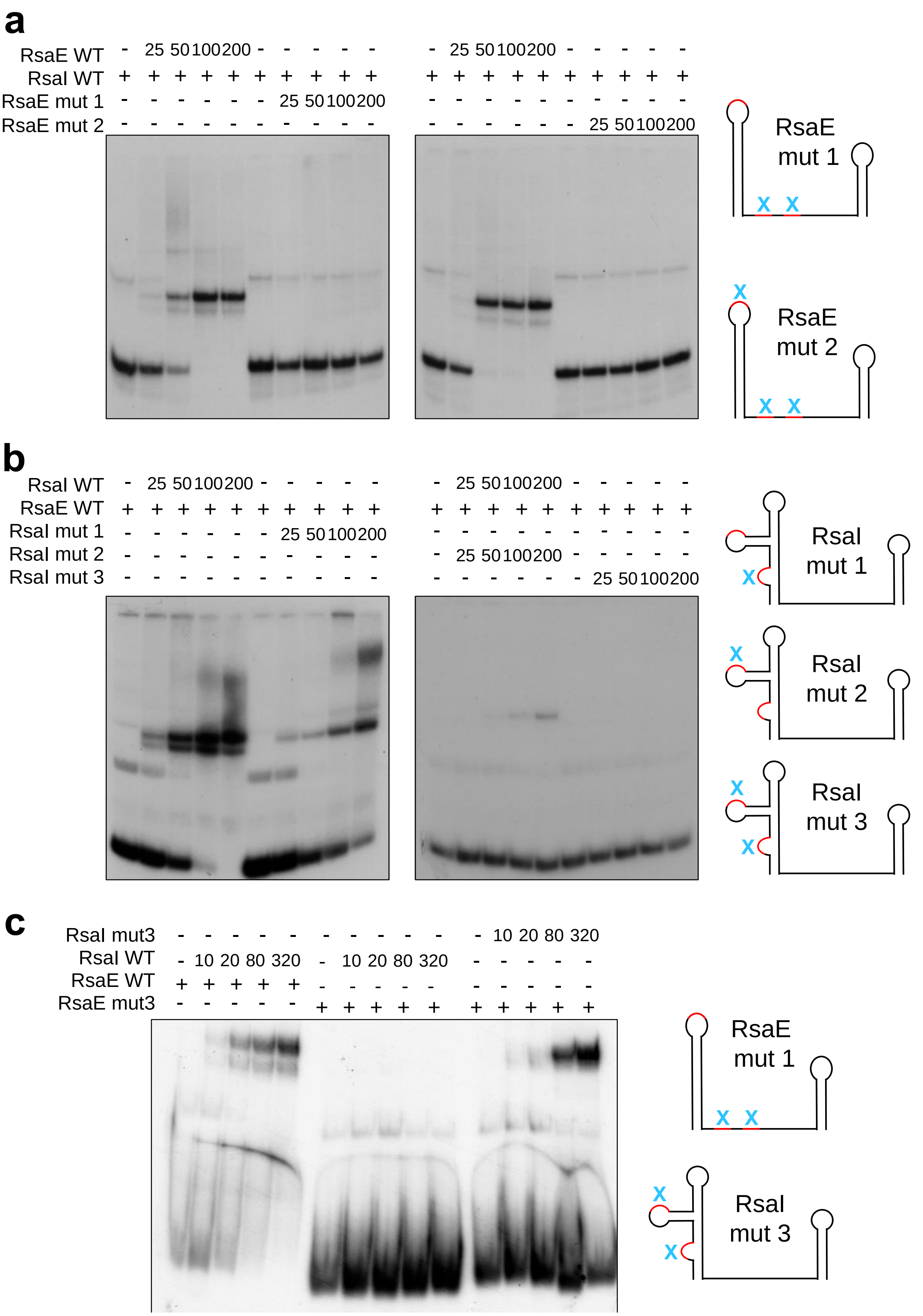
a
RsaE WT - 25 50 100 200 - - - - -
RsaI WT + + + + + + + + + +
RsaE mut 1 - - - - - - 25 50 100 200
RsaE mut 2 - - - - - - - - - -
- 25 50 100 200 - - - - -
+ + + + + + + + + +
- - - - - - - - - -
- - - - - - 25 50 100 200
RsaE mut 1
RsaE mut 2
b
RsaI WT - 25 50 100 200 - - - - -
RsaE WT + + + + + + + + + +
RsaI mut 1 - - - - - - 25 50 100 200
RsaI mut 2 - - - - - - - - - -
RsaI mut 3 - - - - - - - - - -
- 25 50 100 200 - - - - -
+ + + + + + + + + +
- - - - - - - - - -
- 25 50 100 200 - - - - -
- - - - - - 25 50 100 200
RsaI mut 1
RsaI mut 2
RsaI mut 3
c
RsaI mut3 - - - - - - - - - - - 10 20 80 320
RsaI WT - 10 20 80 320 - 10 20 80 320 - - - - -
RsaE WT + + + + + - - - - - - - - - -
RsaE mut3 - - - - - + + + + + + + + + +
RsaE mut 1
RsaI mut 3

Supplementary Figure 11: RsaE and RsaI physically interact in vitro using C- and G-rich motifs.

(a) EMSAs using radiolabelled RsaI and increasing amount of RsaE (mutant) transcripts. The location of the mutations in the RsaE mutants is indicated with a blue X in the secondary structures of RsaE on the right side of the EMSA results. The red regions in the secondary structures indicate the RsaE UCCCC motifs. RsaE values represent molar excess.

(b) EMSAs using radiolabelled RsaE and increasing amount of RsaI (mutant) transcripts. The location of the mutations in the RsaI mutants is indicated with a blue X in the secondary structures of RsaI on the right side of the EMSA results. The red regions in the secondary structures indicate the RsaI G-rich motifs. RsaI values represent molar excess.

(c) Mutations in RsaI that block base-pairing with RsaE can be restored by compensatory mutations in the 3'-UCCCC motifs of RsaE. Shown are of EMSAs using radiolabelled RsaI mut3 transcript (with both G-rich motifs mutated) and increasing amount of RsaE mut3, which has the C's in the 3'UCCCC motifs mutated to G's. RsaI values represent molar excess.

For all the results the uncropped images are shown.

a

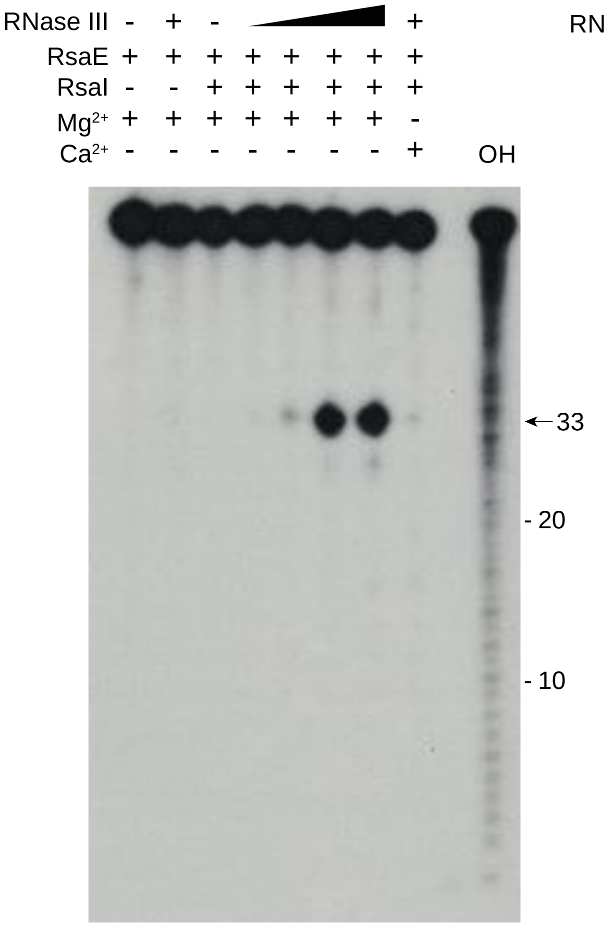

RNase III - + - +
RsaE + + + + + + + +
RsaI - - + + + + + +
Mg2+ + + + + + + + -
Ca2+ - - - - - - - +
OH
33
- 20
- 10

b

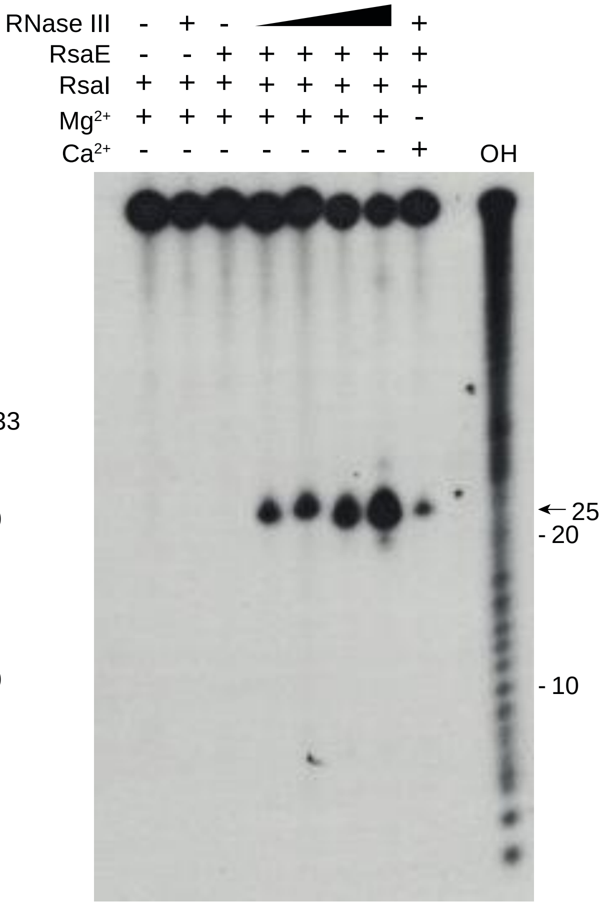

RNase III - + - +
RsaE - - + + + + + +
RsaI + + + + + + + +
Mg2+ + + + + + + + -
Ca2+ - - - - - - - +
OH
25
- 20
- 10

c

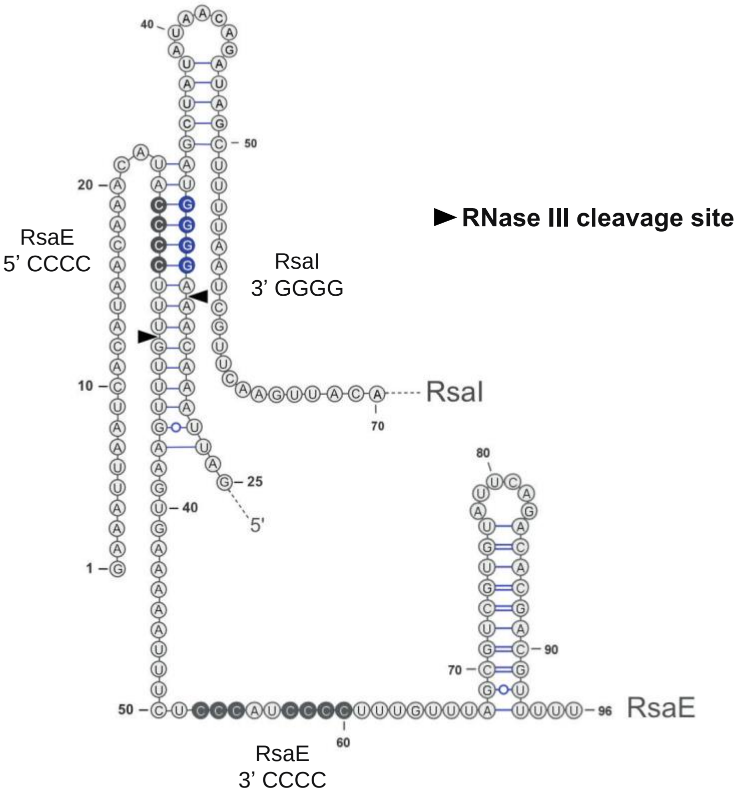

RNase III cleavage site
RsaE
5' CCCC
RsaI
3' GGGG
RsaI
RsaE
RsaE
3' CCCC
5'

Supplementary Figure 12: RNase III cleaves the RsaI-RsaE duplex in vitro.

(a) In vitro cleavage assay performed using ^{32}P-RsaE, RsaI and ^{32}P-RsaE-RsaI duplex in the presence or absence of recombinant RNase III. As RNase III does not cleave dsRNA substrates in the presence of Ca2+, this divalent ion was used as a negative control for non-specific cleavage of the RNA-duplex. An uncropped image is shown.

(b) Same as in (a) but now using ^{32}P-labeled RsaI. An uncropped image is shown.

(c) Secondary structure model of the RsaI-RsaE duplex that is cleaved by RNase III in vitro. The triangles indicate the RNase III cleavage sites in the duplex. The RsaE CCCC and RsaI GGGG motifs are also indicated.

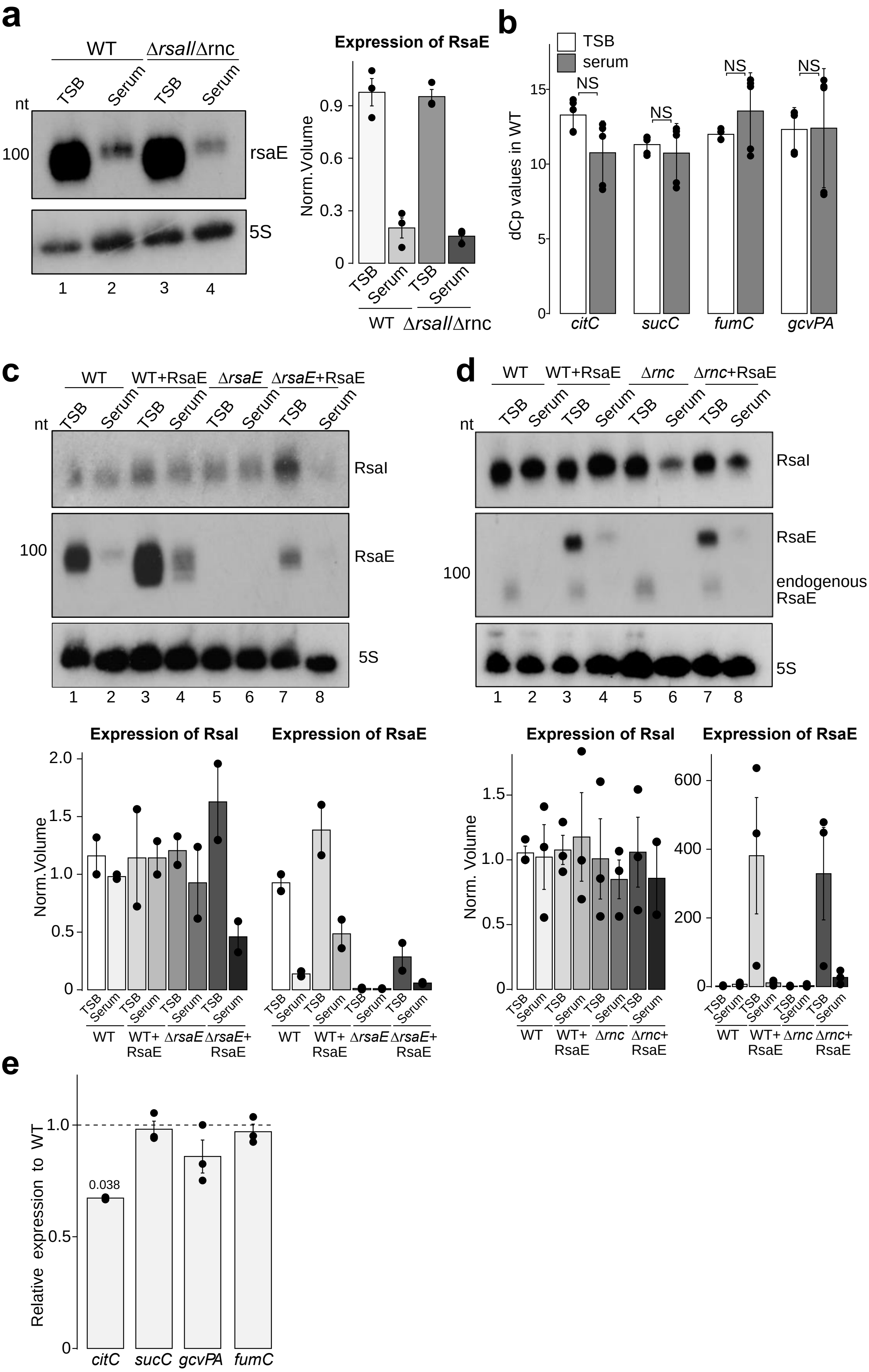
a
WT
Δrsal/Δrnc
nt
TSB
Serum
TSB
Serum
100
rsaE
5S
1
2
3
4
Expression of RsaE
Norm.Volume
0
0.3
0.6
0.9
TSB
Serum
TSB
Serum
WT
Δrsal/Δrnc
b
TSB
serum
dCp values in WT
0
5
10
15
NS
NS
NS
NS
citC
sucC
fumC
gcvPA
c
WT
WT+RsaE
ΔrsaE
ΔrsaE+RsaE
nt
TSB
Serum
TSB
Serum
TSB
Serum
TSB
Serum
RsaI
100
RsaE
5S
1
2
3
4
5
6
7
8
d
WT
WT+RsaE
Δrnc
Δrnc+RsaE
nt
TSB
Serum
TSB
Serum
TSB
Serum
TSB
Serum
RsaI
RsaE
100
endogenous
RsaE
5S
1
2
3
4
5
6
7
8
Expression of RsaI
Expression of RsaE
Norm.Volume
0
0.5
1.0
1.5
2.0
TSB
Serum
WT
WT+
RsaE
ΔrsaE
ΔrsaE+
RsaE
Expression of RsaI
Expression of RsaE
Norm. Volume
0
0.5
1.0
1.5
0
200
400
600
WT
WT+
RsaE
Δrnc
Δrnc+
RsaE
e
Relative expression to WT
0
0.5
1.0
0.038
citC
sucC
gcvPA
fumC

Supplementary Figure 13. Complementary analysis of RsaI on RsaE interaction.

(a) Northern blot analysis of USA300 parental (WT) and *ΔrsaI/Δrnc* double mutant grown in TSB and human serum. Cells were grown as previously described to exponential phase in TSB and then diluted to OD_{600} 0.05 in TSB or human serum and grown for another three hours. Three independent biological replicate experiments were performed, with a representative experiment shown here. The right panel shows the expression of RsaE as measured by ImageQuant software from the autoradiography signal of the Northern blot. The y-axis represents the volume values normalised to the first sample on the blot.

(b) Growth in human serum does not change the expression of metabolic genes targets of RsaE. Shown is the dCp value which represents Cp values normalised by Cp value of 5S used as a reference gene. 'NS' indicates no significant difference between samples (Student's unpaired, two-tailed t-test). For the qPCR analyses RNA from five independent biological replicate experiments was analysed.

(c) Northern blot analysis of USA300 parental (WT), WT with pICS3::RsaE expression under the pAmiA promoter, *rsaE* and *rsaE* with pICS3::RsaE grown in TSB and human serum. Cells were grown as in (a). Two independent replicate experiments were performed, with a representative experiment shown here. Below are shown the expression of RsaI and RsaE overexpression from pICS3 vector as quantified from the above Northern blot.

(d) Northern blot analysis of USA300 parental (WT), WT with pRMC2::RsaE expression under the $P_{xyl/tetO}$ promoter, *rnc* and *rnc* with pRMC2::RsaE grown in TSB and human serum. Cells were grown as in (a) and RsaE expression from the plasmid was induced by addition of anhydrotetracycline (aTc,1µM) to the culture media for 15 minutes. Three independent replicate experiments were performed, with a representative experiment shown here. Below are shown the expression of RsaI and RsaE overexpression from pRMC2 as quantified from the above Northern blot. No significant difference was detected between samples in the quantification of Northern blots (a), (c) and (d).

(e) Expression of RsaE targets in TSB in the WT strain with induced expression of RsaE from pRMC2 as described in (d) relative to the parental USA300 (WT) strain and measured by qPCR. The mean and standard deviation were calculated from three independent experimental replicates (shown as black dots) and three technical replicates. The value in the plot displays a p-value (RsaE over-expression vs WT), obtained using Student's unpaired, two-tailed t-test.

Images and raw data used to generate figures (a-e) are provided in the Source Data file.

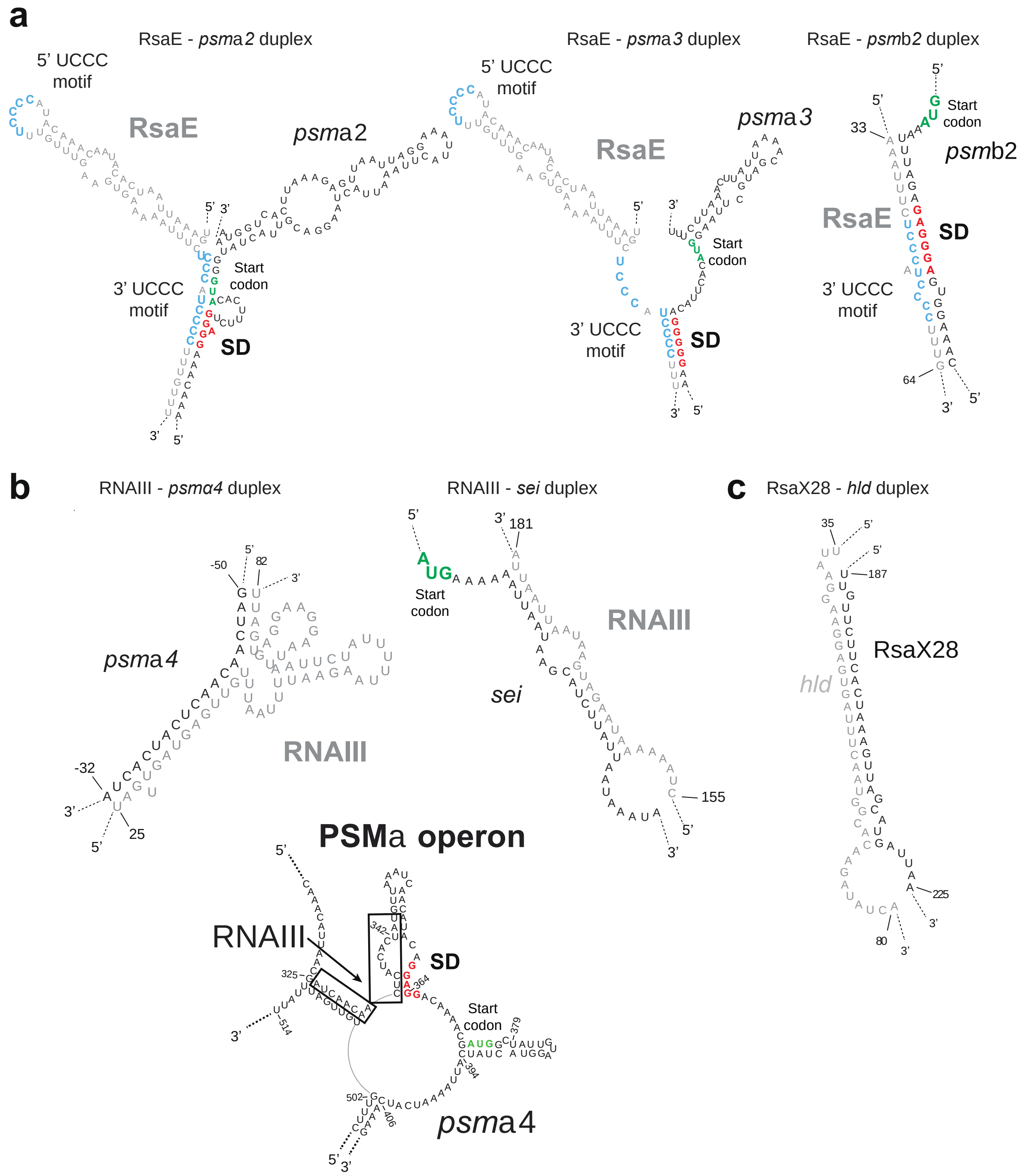

Supplementary Figure 14: The sRNA-toxin interactions identified with RNase III CLASH.

(a) *In silico* predicted interactions between RsaE and α/βPSM transcripts. Chimeras identified in the CLASH data were folded using RNADuplex[23]. The UCCC motifs indicate the known seed sequences of RsaE. The red coloured nucleotides in *psm* mRNA transcripts are the Shine-Dalgarno sequences. The translational start codon is highlighted in green.

(b) As in (a), but now for RNAIII interactions with toxin mRNAs. The bottom structure shows where RNAIII base-pairs in the PSMα operon.

(c) Interaction between RsaX28 and the haemolysin δ mRNA based predicted by the CLASH data.

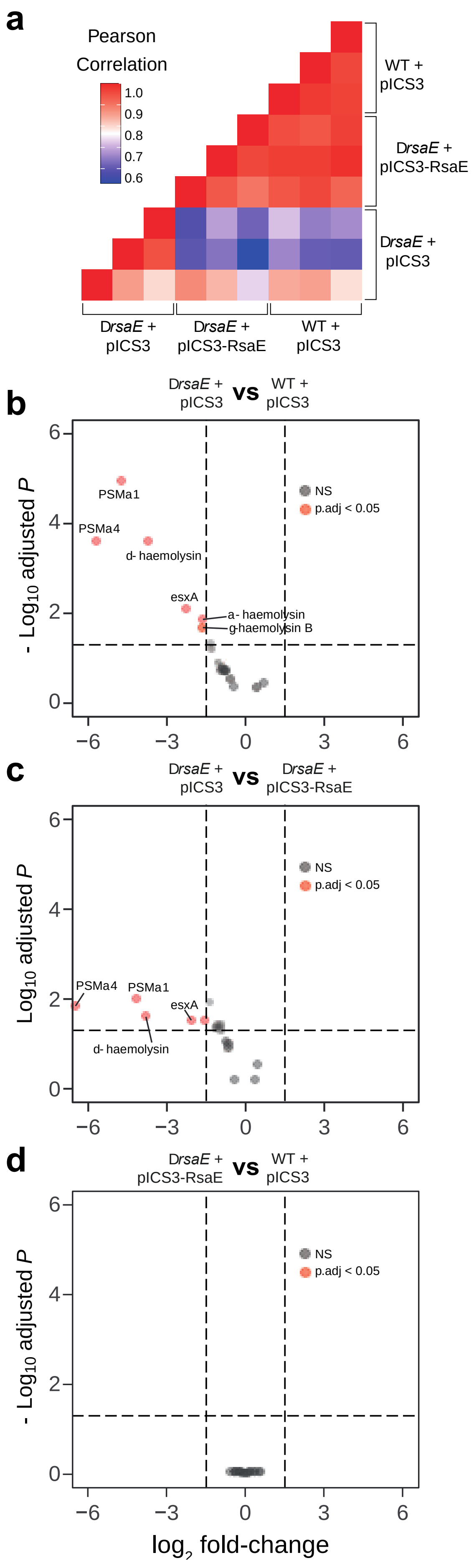
a
Pearson Correlation
1.0
0.9
0.8
0.7
0.6
WT + pICS3
DrsaE + pICS3-RsaE
DrsaE + pICS3
DrsaE + pICS3
DrsaE + pICS3-RsaE
WT + pICS3
b
DrsaE + pICS3 vs WT + pICS3
- Log10 adjusted P
NS
p.adj < 0.05
PSMa1
PSMa4
d- haemolysin
esxA
a- haemolysin
g-haemolysin B
−6
−3
0
3
6
c
DrsaE + pICS3 vs DrsaE + pICS3-RsaE
Log10 adjusted P
NS
p.adj < 0.05
PSMa4
PSMa1
esxA
d- haemolysin
d
DrsaE + pICS3-RsaE vs WT + pICS3
- Log10 adjusted P
NS
p.adj < 0.05
log2 fold-change

Supplementary Figure 15. Mass spectroscopy of culture supernatants from WT, *ΔrsaE* and *ΔrsaE* complemented with pICS3-RsaE.

(a) Pearson correlation coefficients between experiments and biological replicates. Note the strong correlation between individual replicates of one experiment, indicating reproducibility. When RsaE is deleted, the correlation with WT decreases substantially. This effect is reversed when RsaE expression is restored with a plasmid.

(b) Protein levels of indicated toxins in the Δ*rsaE* strain versus the WT. Note the decreased levels of PSMα1 and PSMα4 upon RsaE deletion.

(c) As in (b). but for Δ*rsaE* versus Δ*rsaE* pICS3:RsaE. Note how PSMα1 and PSMα4 levels are largely restored.

(d) As in (b). but for Δ*rsaE* pICS3:RsaE versus the WT. Note that there are no significant changes in protein expression, indicating that RsaE expression is effectively restored by the plasmid. The p-values were generated by empirical Bayes moderated t-test in limma and adjusted by Benjamini-Hochberg method. Only proteins with a log-fold change > 1.5 and $\log_{10}$ adjusted p-values of 1.3 or higher (indicated with dashed lines) were considered.

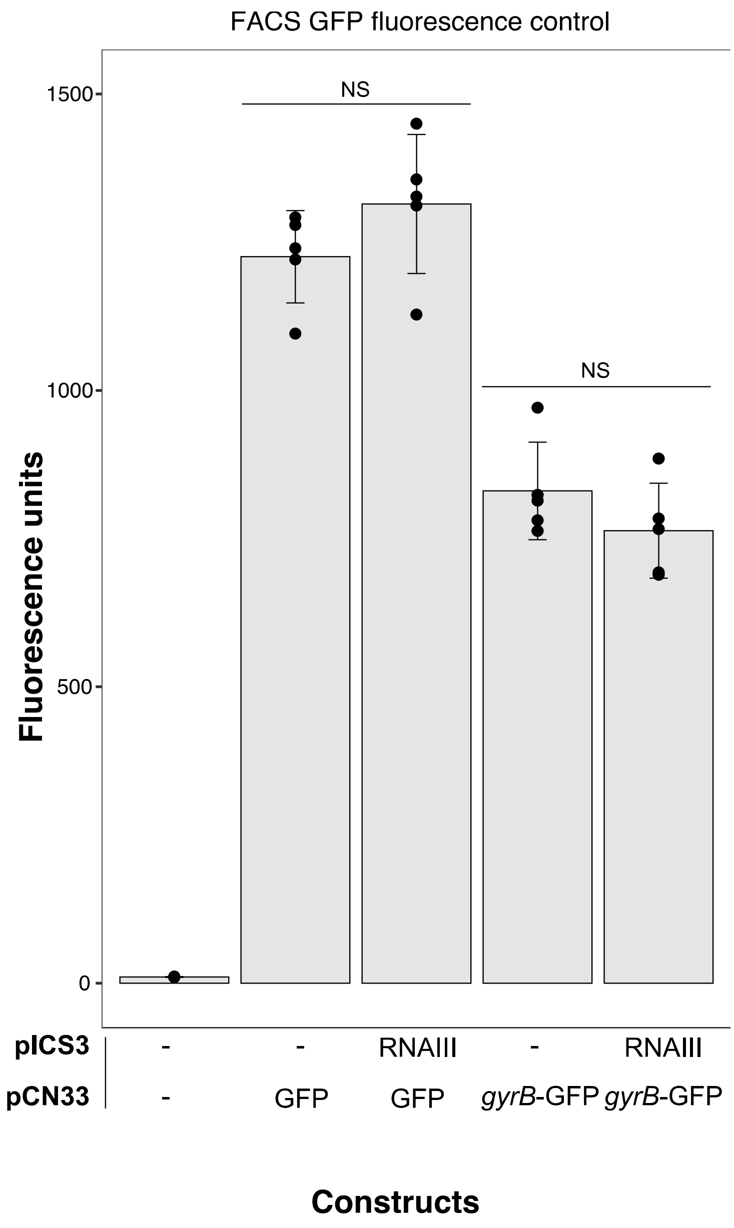

Supplementary Figure 16. Control for FACS analysis. GFP fluorescence is unaffected by RNAIII expression and RNAIII control of *esxA*-GFP is specific, as RNAIII has no effect on an unrelated mRNA-GFP fusion, *gyrB*-GFP. Significant differences between the samples were calculated using a student's two-tailed, unpaired t-test. 'NS' indicates no significant difference (p-value > 0.05). The raw data and representative FACS images are provided in the Source Data File (Figure 7d).

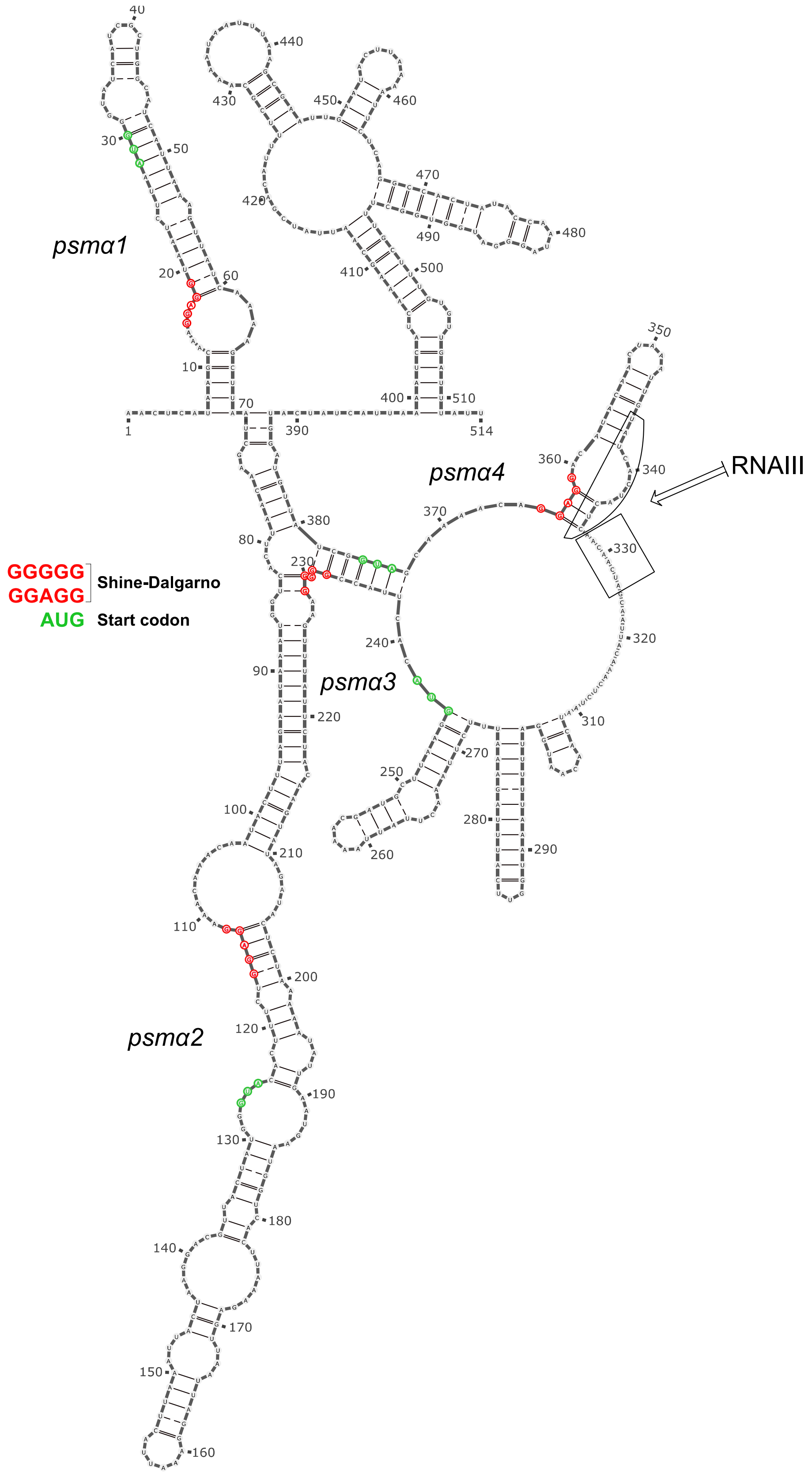

Supplementary Figure 17. Predicted secondary structure of the *psma* operon transcript. This structure was generated using RNAfold[23]. The red nucleotides indicate Shine-Dalgarno (SD) sequences, whereas the green nucleotides indicate translation start codons.

References

1. Kudla, G., Granneman, S., Hahn, D., Beggs, J. D. & Tollervey, D. Cross-linking, ligation, and sequencing of hybrids reveals RNA-RNA interactions in yeast. *Proc. Natl. Acad. Sci. U. S. A.* **108**, 10010–10015 (2011).
2. Helwak, A., Kudla, G., Dudnakova, T. & Tollervey, D. Mapping the human miRNA interactome by CLASH reveals frequent noncanonical binding. *Cell* **153**, 654–665 (2013).
3. Waters, S. A. *et al.* Small RNA interactome of pathogenic E. coli revealed through crosslinking of RN ase E . *EMBO J.* **36**, 374–387 (2017).
4. Iosub, I. A. *et al.* Hfq CLASH uncovers sRNA-target interaction networks linked to nutrient availability adaptation. *eLife* **9**, 1–33 (2020).
5. Romilly, C. *et al.* A Non-Coding RNA Promotes Bacterial Persistence and Decreases Virulence by Regulating a Regulator in Staphylococcus aureus. *PLoS Pathog.* **10**, e1003979 (2014).
6. Tomasini, A. *et al.* The RNA targetome of Staphylococcus aureus non-coding RNA RsaA: Impact on cell surface properties and defense mechanisms. *Nucleic Acids Res.* **45**, 6746–6760 (2017).
7. Geissmann, T. *et al.* A search for small noncoding RNAs in Staphylococcus aureus reveals a conserved sequence motif for regulation. *Nucleic Acids Res.* **37**, 7239–7257 (2009).
8. Rochat, T. *et al.* The conserved regulatory RNA RsaE down-regulates the arginine degradation pathway in Staphylococcus aureus. *Nucleic Acids Res.* **46**, 8803–8816 (2018).
9. Bohn, C. *et al.* Experimental discovery of small RNAs in Staphylococcus aureus reveals a riboregulator of central metabolism. *Nucleic Acids Res.* **38**, 6620–6636 (2010).
10. Eyraud, A., Tattevin, P., Chabelskaya, S. & Felden, B. A small RNA controls a protein regulator involved in antibiotic resistance in Staphylococcus aureus. *Nucleic Acids Res.* **42**, 4892–4905 (2014).

11. Sayed, N., Jousselin, A. & Felden, B. A cis-antisense RNA acts in trans in Staphylococcus aureus to control translation of a human cytolytic peptide. *Nat. Struct. Mol. Biol.* **19**, 105–113 (2012).
12. Geiger, T., Goerke, C., Mainiero, M., Kraus, D. & Wolz, C. The virulence regulator sae of Staphylococcus aureus: Promoter activities and response to phagocytosis-related signals. *J. Bacteriol.* **190**, 3419–3428 (2008).
13. Liu, Q., Yeo, W. S. & Bae, T. The SaeRS two-component system of Staphylococcus aureus. *Genes* vol. 7 (2016).
14. Queck, S. Y. *et al.* RNAIII-Independent Target Gene Control by the agr Quorum-Sensing System: Insight into the Evolution of Virulence Regulation in Staphylococcus aureus. *Mol. Cell* **32**, 150–158 (2008).
15. Norvick, R. P. & Jiang, D. The staphylococcal saeRS system coordinates environmental signals agr quorum sensing. *Microbiology* **149**, 2709–2717 (2003).
16. Lloyd, C. R., Park, S., Fei, J. & Vanderpool, C. K. The Small Protein SgrT Controls Transport Activity of the Glucose-Specific Phosphotransferase System. *J. Bacteriol.* **199**, e00869-16 (2017).
17. Bischoff, M. *et al.* Microarray-based analysis of the Staphylococcus aureus σB regulon. *J. Bacteriol.* **186**, 4085–4099 (2004).
18. Corrigan, R. M. & Foster, T. J. An improved tetracycline-inducible expression vector for Staphylococcus aureus. *Plasmid* **61**, 126–129 (2009).
19. Mäder, U. *et al.* Staphylococcus aureus Transcriptome Architecture: From Laboratory to Infection-Mimicking Conditions. *PLoS Genet.* **12**, e1005962 (2016).
20. Love, M. I., Huber, W. & Anders, S. Moderated estimation of fold change and dispersion for RNA-seq data with DESeq2. *Genome Biol.* **15**, 550 (2014).
21. Malachowa, N. *et al.* Global changes in staphylococcus aureus gene expression in human blood. *PLoS ONE* **6**, e18617 (2011).
22. Liu, W. *et al.* Assessment of bona fide sRNAs in Staphylococcus aureus. *Front. Microbiol.* **9**, 228 (2018).

23. Lorenz, R. *et al.* ViennaRNA Package 2.0. *Algorithms Mol. Biol.* **6**, 26 (2011).

24. Darty, K., Denise, A. & Ponty, Y. VARNA: Interactive drawing and editing of the RNA secondary structure. *Bioinformatics* **25**, 1974–1975 (2009).
