# Supplementary material for: RNase III CLASH in MRSA uncovers sRNA regulatory networks coupling metabolism to toxin expression
Source: Nat Commun. 2022 Jun 22;13:3560. doi: 10.1038/s41467-022-31173-y (PMC9217828; doi:10.1038/s41467-022-31173-y)
Supplement: Supplementary file 3 — Description of Additional Supplementary Information [file 41467_2022_31173_MOESM3_ESM.pdf]

Description of Additional Supplementary Files

Title: Supplementary Data 1

Description: Strains used in this study.

Title: Supplementary Data 2

Description: Oligonucleotides used in this study.

Title: Supplementary Data 3 and 4

Description: We include all our identified interactions in Supplementary Tables 3 (JKD6009) and 4 (USA300). These were filtered for statistical significance (see Methods for details; binomial test and Benjamini-Hochberg adjusted p-value <= 0.05). The 'total' sheet lists all interactions; 'bfsRNA-targets' lists all interactions including a bf sRNA; 'bfsRNA-bfsRNA' lists all interactions where each half of the hybrid is a bf sRNA; 'mRNA-mRNA' lists all interactions where each half of the hybrid is an mRNA; and 'mRNA-mRNA(AS)' lists all interactions between mRNAs and RNAs mapping antisense to mRNAs. Note that this final sheet lists mRNA-mRNA(AS) interactions involving cognate and non-cognate antisense RNAs.

Row name	Purpose
name	Annotated gene name of first RNA fragment from hybrid
rna_class	RNA class (e.g. protein coding, small RNA…) of first RNA fragment from hybrid. Note that the prefix "as_" denotes antisense RNA
firstseq	Sequence of the first RNA fragment from hybrid
start	Genomic position of the first nucleotide of first RNA fragment from hybrid
end	Genomic position of the last nucleotide of first RNA fragment from hybrid
name.1	Annotated gene name of second RNA fragment from hybrid
rna_class.1	RNA class of second RNA fragment from hybrid
secondseq	Sequence of the second RNA fragment from hybrid
start.1	Genomic position of the first nucleotide of second RNA fragment from hybrid
end.1	Genomic position of the last nucleotide of second RNA fragment from hybrid
MFE	Multiple folding energy in kcal/mol of the interacting nucleotides within the hybrid as calculated by RNAduplex
bh_adj_p_value	Benjamini-Hochberg adjusted p value
hyb_count	Number of hybrids identified for given interaction
TSB_hybs	Number of hybrids found in TSB-grown cells

RPMI_hybs	Number of hybrids found in RPMI-shifted cells
LPM7_hybs (Supp. Table 4 only)	Number of hybrids found in LPM pH7-shifted cells
LPM5_hybs (Supp. Table 4 only)	Number of hybrids found in LPM pH5-shifted cells
condition	Conditions in which the interaction was found
intermolecular base-pairs	Sequence from each half of the hybrid predicted to interact through RNAduplex. Each half is separated by '&'
intermolecular_structure	RNA secondary structure of interacting nucleotides expressed in dot-bracket notation, as predicted by RNAduplex

Title: Supplementary Data 5.

Description: Growth curves performed wild-type JKD6009, wild-type USA300 and the two RNase III-HTF strains used in this study.
