# Supplementary material for: RNase III CLASH in MRSA uncovers sRNA regulatory networks coupling metabolism to toxin expression
Source: Nat Commun. 2022 Jun 22;13:3560. doi: 10.1038/s41467-022-31173-y (PMC9217828; doi:10.1038/s41467-022-31173-y)
Supplement: Supplementary file 9 — Reporting Summary [file 41467_2022_31173_MOESM9_ESM.pdf]

nature research

Corresponding author(s): Sander Granneman

Last updated by author(s): May 23, 2022

Reporting Summary

Nature Research wishes to improve the reproducibility of the work that we publish. This form provides structure for consistency and transparency in reporting. For further information on Nature Research policies, see our Editorial Policies and the Editorial Policy Checklist.

Statistics

For all statistical analyses, confirm that the following items are present in the figure legend, table legend, main text, or Methods section.

n/a	Confirmed	
☐	☒	The exact sample size (n) for each experimental group/condition, given as a discrete number and unit of measurement
☐	☒	A statement on whether measurements were taken from distinct samples or whether the same sample was measured repeatedly
☐	☒	The statistical test(s) used AND whether they are one- or two-sided<br>*Only common tests should be described solely by name; describe more complex techniques in the Methods section.*
☒	☐	A description of all covariates tested
☒	☐	A description of any assumptions or corrections, such as tests of normality and adjustment for multiple comparisons
☐	☒	A full description of the statistical parameters including central tendency (e.g. means) or other basic estimates (e.g. regression coefficient) AND variation (e.g. standard deviation) or associated estimates of uncertainty (e.g. confidence intervals)
☐	☒	For null hypothesis testing, the test statistic (e.g. F, t, r) with confidence intervals, effect sizes, degrees of freedom and P value noted<br>*Give P values as exact values whenever suitable.*
☒	☐	For Bayesian analysis, information on the choice of priors and Markov chain Monte Carlo settings
☒	☐	For hierarchical and complex designs, identification of the appropriate level for tests and full reporting of outcomes
☐	☒	Estimates of effect sizes (e.g. Cohen's d, Pearson's r), indicating how they were calculated

Our web collection on statistics for biologists contains articles on many of the points above.

Software and code

Policy information about availability of computer code

Data collection	MaxQuant version 1.6.2.10/ 1.6.10.4351 was used to process the mass-spectrometry data.<br>Rockhoper (version 2.0.3) was used to generate a GFF annotation file from our RNA-seq data.
Data analysis	A description of the data analyses steps performed is described in detail in the Methods section.<br>In addition, test data and test code (in the form of Jupyter notebooks) is available from the repositories showing how to process the data (https://git.ecdf.ed.ac.uk/sgrannem).<br><br>Mapping sequencing reads to the genome:<br>Novoalign (www.novocraft.com ; version 2.07)<br><br>Nanopore sequencing data analyses:<br>pychopper (version 2.5.0; https://github.com/nanoporetech/pychopper.git)<br>minimap2 (version 2.24; https://github.com/lh3/minimap2)<br><br>Time-series data analyses:<br>STEM (version 1.3.13; https://www.cs.cmu.edu/~jernst/stem/)<br><br>Identifying CRE motifs in the Genome:<br>fuzznuc (EMBOSS version 6.6.0.0; https://github.com/google/emboss)<br><br>The scripts for statistical analysis of hyb data is available from https://bitbucket.org/jaitree/hyb_stats/.<br>The FLASH2 algorithm (version 1.2.11) for merging paired reads is available from https://github.com/dstreett/FLASH2.

The hyb pipeline (version 0.0) used for identifying chimeric reads is available from https://github.com/gkudla/hyb.

Samtools (version 1.9; http://www.htslib.org)
bedtools (version 2.27.1; https://github.com/arq5x/bedtools2)
flexbar (version 3.5.0; https://github.com/seqan/flexbar)
vienna2 (version 2.5.0; https://www.tbi.univie.ac.at/RNA/)

Python code and Python pipelines used:
pyCRAC (version 1.5.1; https://git.ecdf.ed.ac.uk/sgrannem/pycrac; https://pypi.org/user/g_ronimo/)
GenomeBrowser packages (version 1.6.3; https://git.ecdf.ed.ac.uk/sgrannem/)
CRAC_pipeline_PE (version 0.6.1; https://git.ecdf.ed.ac.uk/sgrannem/crac_pipelines)
CLASH Pipelines (version 0.0.1; https://git.ecdf.ed.ac.uk/sgrannem/clash-pipelines)
iGraph (version 0.8.0; https://igraph.org/2020/02/14/igraph-0.8.0-python.html)

R code used:
R (4.0.3) (R Core Team, 2020)
https://www.r-project.org/
RStudio (1.4.1103) (Team, 2015)
https://www.rstudio.com/
DESeq2 (Love et al., 2014)
https://bioconductor.org/packages/release/bioc/html/DESeq2.html

For manuscripts utilizing custom algorithms or software that are central to the research but not yet described in published literature, software must be made available to editors and reviewers. We strongly encourage code deposition in a community repository (e.g. GitHub). See the Nature Research guidelines for submitting code & software for further information.

Data

Policy information about availability of data

All manuscripts must include a data availability statement. This statement should provide the following information, where applicable:
- Accession codes, unique identifiers, or web links for publicly available datasets
- A list of figures that have associated raw data
- A description of any restrictions on data availability

The next generation sequencing data have been deposited on the NCBI Gene Expression Omnibus (GEO) with accession number GSE166151. The mass spectrometry proteomics data have been deposited to the ProteomeXchange Consortium via the PRIDE partner repository with the dataset identifier PXD025122.

We also provide a scan of the full images of Northern and Western blots for those cases where we displayed data as cropped images in the Source Data file. Raw data for each plot is also provided. A complete overview of all the CLASH analyses are provided in Supplementary Data files.

Field-specific reporting

Please select the one below that is the best fit for your research. If you are not sure, read the appropriate sections before making your selection.

☒ Life sciences ☐ Behavioural & social sciences ☐ Ecological, evolutionary & environmental sciences

For a reference copy of the document with all sections, see nature.com/documents/nr-reporting-summary-flat.pdf

Life sciences study design

All studies must disclose on these points even when the disclosure is negative.

Sample size	All experiments were performed using biological replicates (2-3). For the Mass-spectrometry, RNA-seq and CLASH analyses at least two independent replicate experiments were performed. Experiments were performed using independent biological replicates (2-3). For the Mass-spectrometry, qPCR, RNA-seq and CLASH analyses at least two experimental replicates and/or three technical replicates were performed. No sample size calculation was performed. Sample sizes were chosen based on the minimal number of samples required to obtain robust statistics (RNA-seq: n>=3; label free mass-spectrometry n>=6; CRAC datasets n>=2)
Data exclusions	No data was excluded.
Replication	Experiments were performed at least twice and often by two independent researchers. To quantify reprodicibility, we calculated Pearson correlations between experimental replicates. Where possible, results from each experimental replicate were displayed in either the main figures or the Extended Figures to illustrate reproducibility. We have also uploaded source files that show the Northern, Western and qPCR results for all experimental replicates.
Randomization	Not applicable as this study did not involve patients or clinical trials.
Blinding	Not applicable as this study did not involve patients or clinical trials.

Reporting for specific materials, systems and methods

We require information from authors about some types of materials, experimental systems and methods used in many studies. Here, indicate whether each material, system or method listed is relevant to your study. If you are not sure if a list item applies to your research, read the appropriate section before selecting a response.

Materials & experimental systems

n/a	Involved in the study
☐	☒ Antibodies
☒	☐ Eukaryotic cell lines
☒	☐ Palaeontology and archaeology
☒	☐ Animals and other organisms
☒	☐ Human research participants
☒	☐ Clinical data
☒	☐ Dual use research of concern

Methods

n/a	Involved in the study
☒	☐ ChIP-seq
☒	☐ Flow cytometry
☒	☐ MRI-based neuroimaging

Antibodies

Antibodies used

anti-FLAG-HRP antibody (clone M2 Sigma-Aldrich A8592; 1:5000)
anti-FLAG M2 Magnetic Beads (Sigma-Aldrich M8823)
anti-TAP Tag polyclonal antibody (ThermoFisher; CAB1001; 1:5000)
goat anti-Rabbit IgG (Invitrogen; A16096; 1:10000)
anti-EsxA (kind Gift from Tracy palmer; https://www.ncl.ac.uk/medical-sciences/people/profile/tracypalmer.html, Newcastle University; 1:1000)

Validation

Anti-FLAG antibodies:
https://www.sigmaaldrich.com/catalog/product/sigma/m8823?lang=en&region=GB

EsxA antibody:
https://www.nature.com/articles/nmicrobiol2016183?WT.feed_name=subjects_bacterial-secretion

Anti-TAP antibody:
https://www.thermofisher.com/antibody/product/TAP-Tag-Antibody-Polyclonal/CAB1001
