# Supplementary material for: RNase III CLASH in MRSA uncovers sRNA regulatory networks coupling metabolism to toxin expression
Source: Nat Commun. 2022 Jun 22;13:3560. doi: 10.1038/s41467-022-31173-y (PMC9217828; doi:10.1038/s41467-022-31173-y)
Supplement: Supplementary file 10 — Source Code [file 41467_2022_31173_MOESM10_ESM.zip › Source_Code/Repositories/pycrac-master.zip!/pycrac-master/The_pyCRAC_Manual.pdf]

The pyCRAC Manual

version 1.3.2

Shaun Webb, Grzegorz Kudla and Sander Granneman

Sander Granneman
sgrannem@.ed.ac.uk

07-05-2018

Contents

1 Overview **1**

1.1 pyCRAC publication 1

1.2 Background 1

1.3 Summary of the available tools 2

1.4 Why pyCRAC? 2

1.5 License and availability 4

1.6 Contributors to pyCRAC 4

2 Installation requirements **7**

3 Quick start guide **9**

3.1 How to install pyCRAC 9

3.2 PyCRAC test data 9

3.3 Checking your GTF annotation file 10

4 General usage information **11**

4.1 pyCRAC tools options documentation 11

4.2 Genes and transcripts 11

4.3 Aligning reads to the genomic reference sequence 12

4.4 Supported file formats 13

4.4.1 Input files must be tab-delimited 13

4.4.2 Processing and manipulating GTF feature files 14

4.4.3 Support for other tabular annotation formats 14

4.4.4 Chromosomal sequence files also need to be in tab-delimited format 15

4.4.5 Novoalign and BAM/SAM formats 16

4.4.6 Handling paired-end data sets 16

4.5 Data processing fundamentals 16

4.5.1 Common options 16

4.5.2 Read sequence correction 18

4.5.3 Calculating overlap between reads and genomic features 19
4.5.4 Alignment qualities and alignment scores 19
4.5.5 How to deal with untranslated (UTRs) and flanking regions and how manually set their coordinates 20
4.5.6 Reads, cDNAs, blocks, clusters and multiple alignment locations . 22
4.5.7 Using genomic and coding sequences as reference 24
4.5.8 Filtering the data for reads with mutations 24
4.5.9 Additional common options 24
4.5.10 File handling options 25

5 The pyCRAC tools 26

5.1 pyBarcodeFilter 26
5.1.1 Usage and option summary 27
5.1.2 Output files 29
5.2 pyReadCounters 29
5.2.1 Usage and option summary 29
5.2.2 Default behaviour 29
5.2.3 Command line examples 30
5.2.4 Output files 33
5.3 pyClusterReads 36
5.3.1 Default behaviour 36
5.3.2 Output files 37
5.3.3 Command line examples 39
5.4 pyPileup and pyReadAligner 40
5.4.1 Usage and option summary 41
5.4.2 Default behaviour 41
5.4.3 Output files 42
5.4.4 Command line examples 42
5.5 pyMotif 44
5.5.1 Motif search algorithm 44
5.5.2 Usage and option summary 45
5.5.3 Default behaviour 45
5.5.4 pyMotif-specific options 46
5.5.5 Output files 46
5.5.6 Command line examples 47
5.6 pyBinCollector 49
5.6.1 Usage and option summary 49

5.6.2 Default behaviour . . . 49
5.6.3 Output files . . . 51
5.6.4 Command line examples . . . 51
5.7 pyCalculateFDRs . . . 56
5.7.1 Input and output files . . . 58
5.7.2 Selecting significant clusters using pyCalculateFDRs and bedtools 59
5.8 pyCalculateMutationFrequencies . . . 61
5.8.1 Command line examples . . . 61

6 The pyCRAC scripts 62
6.1 Utilities . . . 62
6.1.1 pyAlignment2Tab.py . . . 62
6.1.2 pyFasta2Tab.py . . . 63
6.1.3 pyCalculateChromosomeLengths.py . . . 63
6.2 Processing fastq and fasta formatted data . . . 64
6.2.1 Removing PCR duplicates by collapsing the data . . . 64
6.2.2 Removing PCR duplicates using random nucleotide information . 66
6.3 GTF file manipulation tools . . . 67
6.3.1 pyCheckGTFfile.py . . . 67
6.3.2 pyExtractLinesFromGTF.py . . . 69
6.3.3 pyGTF2bed . . . 69
6.3.4 pyGTF2bedGraph . . . 70
6.3.5 pyGTF2sgr.py . . . 72
6.3.6 pyGetGTFSources.py . . . 74
6.3.7 pyGetGeneNamesFromGTF.py . . . 75
6.3.8 pySelectMotifsFromGTF.py . . . 76
6.3.9 pyNormalizeIntervalLengths.py . . . 77

List of Figures

4.1 Schematic representation of how pyCRAC tools calculate chromosomal mapping positions using mutation information stored in novo or SAM/BAM files 13

4.2 Sequence correction and highlighting in pyCRAC 18

4.3 Calculating overlap between read mapping positions and genomic features. An example showing the effects of changing the overlap setting in pyCRAC tools. 19

4.4 Example showing how to add UTR coordinates to GTF annotation files. UTR coordinates are indicated as exons or UTRs 21

4.5 The GTF2 parser calculates 5 and 3 UTR coordinates by comparing start and end positions of "exon" and "CDS" features. **NOTE** that the stop codon is not included in CDS features. UTR coordinates can also be included as separate features, indicated as 5UTR and 3UTR, respectively. TSS indicates the transcriptional start site, whereas pA indicates the polyadenylation site. Red arrows indicate 300 nucleotide long 5 and 3 UTR sequences. 21

4.6 Examples showing removal of putative PCR duplicates (blocks) and cluster generation. Shown is a schematic representation of a gene (*YFG1*) containing two exons and one intron. Reads and clusters are indicated as thick black lines. Mutations are indicated as asterisks. (A) All reads that mapped to *YFG1* are displayed. (B) PCR duplicates or "blocks" are condensed into one cDNA sequence using pyFastqDuplicateRemover. Note that positions of mutations are considered when removing duplicates, however, reads with the same coordinates are still counted as a single cDNA during cluster formation. (C) Clusters generated from at least two overlapping cDNA sequences using pyClusterReads. This step removes the reads forming the large block in the second exon of the gene. (D) Clusters generated from at least five unique cDNAs. This removes the cDNA sequences mapped to the 5' region of the gene. 23

5.1 Example of a header after splitting randomly-barcoded data with pyBarcodeFilter. If the barcode sequence file indicates barcodes with random nucleotides, the tool will remove the barcode and attach the random barcode sequence (red) to the header with two hashes (blue). 27
5.2 Example plots indicating high-and low-complexity datasets 34
5.3 Example of a pyReadCounters hittable output file 34
5.4 Example of a pyReadCounters cDNAs GTF output file 35
5.5 A few lines from a pyClusterReads GTF output file 38
5.6 The pyPileup -s coding flag can be used to remove intron sequences. Shown are two plots displaying the read distribution over a gene called *YFG1*. In the right panel, only the hits that mapped to exons are displayed. 44
5.7 A Few lines from a k-mer_Z_scores.txt file generated by pyMotif. The first column shows the k-mer sequence, the second column the Z-score for that motif and the third column shows the mutation frequency, which indicates the percentage of motifs that have at least one mutation in the sequence. 47
5.8 A Few lines from a pyMotif 'top_k-mers_in_features' GTF file. 48
5.9 Shown is a section of a pyBinCollector pileup file generated using the --outputall flag. Gene names are listed in the first column and each following column shows the read densities for each bin. 52
5.10 Section of a pyBinCollector pileup file generated by including the --outputall flag. Gene names are listed in the first column and each following column shows the read densities for each bin. 52
5.11 Distribution of deletions in and around the CUUG motif identified in Nab3 CRAC data 55
5.12 A few lines from a pyCalculateFDRs.py GTF output file. 58
5.13 A few lines from a pyCalculateFDRs.py log file. 59

6.1 PyAlignment2tab can generate colourful tab formatted alignments in the terminal. The example here shows a handful of reads from PAR-CLIP and CRAC data aligned to the yeast *SUP19* tRNA gene. The plot above the alignment shows the corresponding pyPileup result. The gaps in the sequence show deletions, whereas substitutions are indicated in lower case. 63
6.2 An example of a pyCalculateChromosomeLenghts.py output file. 64

6.3 pyFastqDuplicateRemover scans the header for the presence of two hashes near the end (blue) and assumes that the sequence following these hashes (red) is the random barcode sequence. If it encounters two identical (paired-) sequences with the same random barcode sequences, it assumes they are PCR duplicates and collapse them into one sequence. The orange characters indicate that this header originates from the forward sequencing reaction. The green characters indicate an Illumina indexing sequence. . 67

6.4 Example showing what happens to the data during pyBarcodeFilter and pyFastqDuplicateRemover processing steps. The random barcode sequence is indicated in red, where as the barcode for the experiment is indicated in blue. If the barcode list file contains random nucleotide (see Table 5.1) then the pyBarcodeFilter tool will attach two hashes followed by the random barcode sequence to the header and remove the barcode from the sequence. The pyFastqDuplicateRemover tool then collapses the data and converts the fastq entry into the fasta format and included the random nucleotide sequence (red) and the number of identical sequences it found in the raw data (orange) 68

List of Tables

1.1 Overview of main tools in the pyCRAC 3
1.2 Overview of additional tools packaged with pyCRAC. All scripts have help menus, which can be accessed using the -h or --help flag. 6

4.1 Example entries from a *Saccharomyces cerevisiae* GTF feature file. Each column indicates a separate field in the gtf file 14
4.2 Meaning of each individual column in GTF files 15
4.3 Overview and description of frequently used pyCRAC options. The "command" column indicates the string that needs to be added to the command line in order to use the option. 17
4.4 Explanation of the meaning of the terms reads, cDNAs, blocks and clusters 22

5.1 Example of a barcode text file 27
5.2 Overview of output files generated by pyMotif 46

Chapter 1

Overview

1.1 pyCRAC publication

We have recently published a manuscript in Genome Biology (see Web et al, Genome Biology 2014) where we used the pyCRAC tools to analyse Nrd1 and Nab3 binding sites in yeast. This manuscript should give you a good idea of what you can do with the pyCRAC scripts.

1.2 Background

The development of the CLIP and CRAC UV cross-linking and cDNA cloning techniques allowed the identification of sites of direct protein-RNA interaction in vivo. These methods have greatly improved our understanding of function of many RNA binding proteins in RNA metabolism and the assembly of macromolecular ribonucleoprotein complexes.The combination of CLIP or CRAC with high-throughput sequencing (e.g. HITS-CLIP) has substantially increased the sensitivity of the methodology and provided an unparalleled capability to identify protein-RNA interactions transcriptome-wide. However, the analysis of such high-throughput datasets can be daunting and often demands more than a basic knowledge in bioinformatics and computer programming. When we started doing CRAC experiments in David Tollerveys lab most of us had little or no experience with programming, or with using software from a terminal. We managed to get a significant amount of work done using existing tools, including SAM tools and programs on our Galaxy web server, however more thorough analyses of the CRAC or CLIP high-throughput sequencing data required different options and adding these to existing programs was not always straightforward. Eventually we wrote most of the programs ourselves because this was often faster than trying to change existing ones. At some point almost everybody in the Tollervey lab started applying the CRAC technique to his or her favourite protein. Many

wanted to be able to analyse their own CRAC data but few had experience in data analysis or programming. There was a need for relatively simple but also flexible tools that allowed users with little programming experience to do some analyses on CRAC of CLIP data. For this purpose, we have compiled a set of user-friendly Python software tools, called pyCRAC, that should simplify basic analyses of CLIP/CRAC high-throughput sequence data.

CLIP/CRAC cDNA preparation protocols generate directional cDNA libraries and the sequencing data will contain strand information. PyCRAC was therefore designed to specifically tackle directional libraries and reports sense and anti-sense hits. Some features in pyCRAC might be useful for the analysis of data generated by other transcriptome wide sequencing applications. **NOTE!!** If you want to analyse ChIP or RIPseq data you need to use the --ignorestrand flag with pyCRAC tools. All reads that map to genomic features, including reads that map anti-sense, will be counted as 'sense' reads.

This manual was written to describe the functionality of the pyCRAC tools by using illustrations and focuses on the use of pyCRAC tools via command line. However, we have also made most of the pyCRAC tools compatible with the Galaxy web-based interface and this package can be downloaded from the Galaxy tool-shed at `http://toolshed.g2.bx.psu.edu/`. The Galaxy pyCRAC package has the same functionalities as the command line version and the information in this manual is also applicable to the Galaxy version.

We hope that this document will be sufficient to get you started with pyCRAC and give you a sense of the usefulness of the pyCRAC tools. If you have any further questions, please contact me by e-mail sgrannem@staffmail.ed.ac.uk

1.3 Summary of the available tools

PyCRAC supports a large number of operations for analysing CRAC/CLIP high-throughput data sets. Table 1.1 summarises the main pyCRAC tools and provides a brief description of their functionality. The functionality of these tools is discussed in detail in Chapter 5. In addition, we have also included a small number of useful scripts that can convert output file formats or extract information from various files (see Table 1.2). The main tools are discussed in Chapter 5, whereas the scripts are discussed in Chapter 6.

1.4 Why pyCRAC?

A large number of excellent tools have emerged in recent years, designed to process data from various high-throughput sequencing applications. Several of these tools have

Table 1.1: Overview of main tools in the pyCRAC

Utility	Description
pyBarcodeFilter	Takes raw FASTQ data and a list of barcodes and splits the data based on barcode sequences at the 5' ends of reads. Also produces barcode statistics file.
pyReadCounters	Produces a gene/transcript hittable file, including a table describing hits in UTRs and introns, .sgr and GTF files to visualise the reads in genome browsers. Finally, the program produces a read statistics file, which provides information about the complexity of the dataset.
pyClusterReads.py	Takes a GTF data file and generates clusters from read or other interval coordinates. Produces a GTF output file with cluster intervals and overlapping genomic features.
pyPileup	Produces pileups containing the number of hits, substitutions and deletions for each nucleotide covered by reads in specific genes or genomic regions
pyReadAligner	Generates multiple sequence alignments for reads mapped to individual genes or genomic regions. Produces a fasta output file.
pyMotif	Looks for enriched sequence motifs in high-throughput sequencing data. Produces a GTF type output file with coordinates and Z-scores for enriched motifs. The GTF file can be visualised in genome browsers.
pyBinCollector	Allows the user to generate genome-wide coverage plots. Normalises gene lengths by dividing genes into a fixed number of bins and then calculates the hit density in each bin. The program also allows the user to input specific bin numbers to extract blocks/clusters present in these bins.
pyCalculateFDRs.py	Takes interval information in GTF or bed format and calculates False Discovery Rates (FDRs).
pyCalculateMutationFrequencies.py	Takes an interval file and a pyReadCounters GTF file and calculates substitution and deletion frequencies fore each interval.

similar functionalities as some of the pyCRAC tools and in some areas are more advanced. Why another set of tools? After having analysed numerous high-throughput sequencing datasets, we realised that CRAC data were different in many aspects from other high-throughput sequencing data and we had to use different approaches to tackle the data. We needed options that were not available with existing programs and in many cases it was not immediately clear how to add new features. The development of pyCRAC was driven by a need for flexible, user-friendly and coherent set of tools tailored more specifically to handle CRAC/CLIP data. We believe that one of the strengths of pyCRAC package is the high degree of standardisation. Many of the tools have the same options that work in the exact same way and we hope that using human readable GTF output files will

stimulate data sharing between groups. Moreover, we have tried to make pyCRAC as user-friendly as possible, so that laboratories that are planning on doing CRAC or CLIP experiments but do not have a lot of experience in processing the high-throughput data, have the opportunity to do basic analyses on their data. In addition, we believe that for experienced python programmers it is relatively easy to add new options, making it possible to adapt pyCRAC to their specific needs. PyCRAC was built on top of a collection of python modules, several of which were written specifically for pyCRAC. These could serve as a foundation for the improvement of pyCRAC or rapid development of novel high-throughput sequencing programs. For those that are interested in using the pyCRAC modules for programming purposes, we will provide more documentation in the near future on our bitbucket website: `http://bitbucket.org/sgrann/pycrac/wiki/Home`

We also highly recommend python programmers to look at HTseq, an excellent toolbox that offers a comprehensive python programming framework specifically designed to tackle high-throughput sequencing data `http://www-huber.embl.de/users/anders/HTSeq/doc/overview.html`.

1.5 License and availability

This software is provided 'as-is', without any express or implied warranty. In no event will the authors be held liable for any damages arising from the use of this software. Permission is granted to anyone to use this software for any purpose, including commercial applications, and to alter it and redistribute it freely, subject to the following restrictions:

1. The origin of this software must not be misrepresented; you must not claim that you wrote the original software. If you use this software in a product, an acknowledgment in the product documentation would be appreciated but is not required.

2. Altered source versions must be plainly marked as such, and must not be misrepresented as being the original software.

3. This notice may not be removed or altered from any source distribution.

1.6 Contributors to pyCRAC

We would like to thank Rebecca Holmes, Louise McGibbon, Jai Tree and Alex Tuck for testing the pyCRAC tools on their own CRAC data, for helpful suggestions and help with debugging the scripts. Special thanks to Stamatina Fragkogianni for profiling some of the

code and for her help with parallelising some of the pyCRAC modules (to be included in future pyCRAC versions). We would also like to thank Christos Josephides for helpful suggestions on using numpy. Finally, a million thanks to David Tollervey for his support during the development of pyCRAC and the many beta testers outside our university who helped us improve the package. We welcome pyCRAC users to make suggestions/ provide constructive criticism and encourage any contributions, as we are keen to further improve the pyCRAC tools. In particular, we are looking for developers interested in helping us to improve the speed of the tools by implementing more Cython code and we are in the process of parallelising some of the python scripts.

Table 1.2: Overview of additional tools packaged with pyCRAC. All scripts have help menus, which can be accessed using the -h or --help flag.

**Utility**	**Description**
pyAlignment2Tab.py	Converts fasta to the tabular format.
pyFasta2tab.py	Generates multiple sequence alignments for reads mapped to individual genes or genomic regions. Produces a fasta output file.
pyCalculateChromosomeLengths.py	Takes a genome sequence in fasta or tab format and generates a tab-delimited file showing chromosome name and chromosome length.
**Processing of fastq and fasta-formatted data:**	
pyFastqJoiner	Joins two paired-end fasta or fastq files.
pyFastqSplitter	Splits joined paired-fastq or fasta files.
pyFastqDuplicateRemover	Removes identical sequences from fastq and fasta files and returns a fasta file with collapsed data. Can also process paired-end data.
**GTF file manipulation tools:**	
pyGTF2bed.py	Converts GTF files to the bed 6 format. Gene names present in the GTF file will be included in the bed file.
pyGTF2bedGraph.py	Generates bedgraph files for each strand. An homage to bedtools genomecov. Takes a pyReadCounters GTF file as input file. Can also output bedGraph files for substitutions and deletions.
pybed2GTF.py	Does the opposite of pyGTF2bed. Requires a GTF annotation file and adds gene_names and gene_ids to the output file.
pyCheckGTFfile.py	Renames duplicated gene_names in your GTF annotation file.
pyExtractLinesFromGTF.py	Extracts lines from a GTF file that contain gene names of interest.
pyGetGTFSources.py	Extracts source names from the second column in a GTF file.
pyGetGeneNamesFromGTF.py	Extracts and counts all gene names from a GTF file.
pyGTF2sgr.py	Takes a pyReadCounters of pyMotif GTF file and converts it into a sgr files for each strand. Another homage to bedtools genomcov but has a few more options and provides some more flexibility.
pyNormalizeIntervalLengths.py	Allows you to extend the interval length to a specific value or to set a minimum length for an interval. Useful if you want to extend the size of intervals reported by pyCRAC programs.
pySelectMotifsFromGTF.py	Extracts your favourite k-mer sequence from pyMotif GTF output files.

Chapter 2

Installation requirements

The pyCRAC tools were intended to run from the terminal on Unix/OS X and Linux based operating systems; however, we are in the process of making pyCRAC compatible with the Galaxy web-based interface, allowing users not familiar with the terminal to analyse their data in a web browser. These tools will be made available at the Galaxy tool-shed (`http://toolshed.g2.bx.psu.edu/`). PyCRAC tools may run on Windows operating systems but this has not been tested and will not be actively supported. PyCRAC requires Python 2.7 or higher, but is not yet compatible with Python 3.x. PyCRAC requires Cython, numpy (1.5.1 and up), pysam and bpython, which should be automatically downloaded and installed during the pyCRAC installation process. OS X users will also need to install Xcode developer tools, which can be downloaded from the App store or found on the OS X installation disk. Users running OSX Mountain Lion need to manually download the 'Command line tools' via Xcode. Go to preferences, click on Downloads and click on the 'install' button for the Command line tools. PyCRAC GTF and sgr output files can be visualised in the bioviz integrated genome browser (IGB, `http://bioviz.org/igb/`). We also routinely use the UCSC genome browser to visualise data from GTF files (`http://genome.ucsc.edu`). For OS X users that have python 2.6 or lower on their system, we would recommend downloading the Enthought Canopy python distribution (`https://www.enthought.com/products/canopy/`), which is freely available to academics. This package installs python 2.7, the latest version of numpy and various other modules required to run pyCRAC. Linux/Unix users can update python using distribution specific repositories. With some OSX versions we have experienced some problems with the pysam module during the installation. This usually was because Cython or pysam was not correctly installed. If you experience problems with this, please contact the authors. To run pyCRAC, a computer with 4 GB of RAM is often sufficient when working with bacterial or yeast genomes; however, to analyse CRAC data from higher eukaryotes (i.e. human, mouse) 16 GB or more is rec-

ommended, particularly when dealing with large and complex datasets. In some of the examples we use BEDTools to process data. BEDTools is a suite of flexible programs that allow comparison of large sets of genomic features and can be very useful to manipulate GTF output files generated by pyCRAC tools. BEDTools can be downloaded from `http://code.google.com/p/bedtools`.

Chapter 3

Quick start guide

3.1 How to install pyCRAC

To install pyCRAC, go to the terminal and to the directory containing the setup.py installation file. If you downloaded the pyCRAC package to your desktop, change the directory to the pyCRAC folder in the terminal and use the following command to install pyCRAC:

```
sudo python setup.py install
```

If this command does not work, try running sudo python setup.py build first and then re-run the install command. The installer will generate a pyCRAC folder in the */user/local/* directory and installs various annotation files and genomic sequences in the "db" folder. On some linux distributions python easy_install and python development (python dev) files need to be installed prior to installing pyCRAC.

```
pyReadCounters -h
```

This should display a detailed pyReadCounters.py help menu. To run pyCRAC scripts you basically need to provide the name of the script, locations to various input files and you can add numerous options. An example is shown below. Additional usage examples and detailed explanations are described in Chapters 4 and 5 of this manual.

3.2 PyCRAC test data

The pyCRAC package contains some test data that the program uses during installation to check if all the tools are installed properly. By default the test data is placed in the

/usr/local/pyCRAC/tests folder and contains a test.novo file. This novo file was generated from *Saccharomyces cerevisiae* CRAC data and only contains mapped reads and can be used to test the various settings in pyCRAC. After installation, you can find a folder named "db" in the */usr/local/pyCRAC* directory, which contains a *Saccharomyces cerevisiae* FASTA and a GTF annotation file.

```
pyReadCounters.py -f SolexaData.sam --file_type=sam --gtf=yeast.gtf
```

This command runs the pyReadCounters.py program using default settings. This reports overlap between read sequences and genomic features, using the SolexaData.sam file and yeast GTF feature file that contains chromosomal coordinates of genomic features.

3.3 Checking your GTF annotation file

All the pyCRAC tools heavily rely on GTF annotation files. These can be obtained from many sources (such as UCSC or ENSEMBL) and can contain mistakes. These include duplicated gene_name and/or gene_id features. The GTF parser included in pyCRAC stores both gene_id and gene_name annotations and sometimes the same gene_name annotations are used for different gene_id numbers. This can lead to errors in the data processing and it is critical that you make sure that this is not the case for your GTF file. This can be a problem in recent mouse and human GTF annotation files from ENSEMBL. Before you start, we would recommend using the pyCheckGTFfile.py script to check your GTF annotation file for any duplicate names.

```
pyCheckGTFfile.py --gtf=myfavhumangtf.gtf -o mycorrectedfavgtf.gtf
```

IMPORTANT! We strongly recommend downloading the ENSEMBL igenomes from the Illumina website:

`https://support.illumina.com/sequencing/sequencing_software/igenome.ilmn`

Chapter 4

General usage information

4.1 pyCRAC tools options documentation

Some pyCRAC tools have a large number of options. To make pyCRAC programs as user-friendly as possible, we have included help menus with detailed instructions for each option present in the pyCRAC programs. More detailed explanation on how to use these options is discussed in section 4.5. To access the help menu, use the "-h: or "--help" option in the command line. An example is shown on the next page. The options are divided into common options used by many pyCRAC tools, tool-specific options and file input options.

4.2 Genes and transcripts

In this documentation we quite regularly use the terms *genes* and *transcripts* and it is important to understand their definition, which are based on the GTF2.2 specification. In higher eukaryotes, transcription of genes rarely generates a single transcript. Alternative splicing and transcription initiation at different sites within a gene can give rise to numerous different transcripts. When we use the term *gene*, we refer to **ALL** the transcribed sequences within that gene. This includes 5' and 3' untranslated regions (UTRs), exons and introns, from transcription start site to the poly-adenylation sites (see Figure 4.5). Hence when we refer to the genomic sequence of a gene, the sequence includes all of these features. When we use the terms *transcript coordinates* we refer to chromosomal coordinates for individual RNA transcripts encoded within a gene.

4.3 Aligning reads to the genomic reference sequence

The pyCRAC package has specifically been designed to tackle reads that have been aligned to genomic reference sequences. Hence, pyCRAC will **NOT** work on data mapped to cDNA sequences. This may be included in later versions if there is sufficient demand for it.

To make the CRAC cDNA libraries, the cross-linked protein has to be removed by proteinase K treatment. This presumably does not remove UV cross-linked amino-acid(s). Others and we have noticed that during cDNA synthesis the reverse transcriptase can jump over the cross-linked amino acid, frequently introducing deletions in the cDNA at specific positions. In many cases these deletions appear to highlight the UV cross-linking site(s) and identification of these sites is an important part of the data analyses. Because is not uncommon to find reads containing gaps of several nucleotides it is important to use a program that can accurately align reads with deletions to the reference sequence. A list of sequence alignment tools can be found on the following website: `http://en.wikipedia.org/wiki/List_of_sequence_alignment_software`. We frequently use the Novoalign gapped reference sequence aligner (`www.novocraft.com`), because it can accurately align reads with a high number of nucleotide mismatches. Another advantage of Novoalign is that it has the option to remove trailing adapter sequences. Read lengths in CRAC high-throughput data are in the range of 15 to 100nt, and therefore adapter sequences quite often contaminate the data sets. Adapter trimming can also be performed using the fastx toolkit from the Hannon lab (`http://hannonlab.cshl.edu/fastx_toolkit/`), which is an excellent set of tools for post processing of FASTQ data. We would also recommend trying flexbar (`http://sourceforge.net/p/flexbar/wiki/Manual/`).The pyCRAC tools fully support the native Novoalign output format. The pyCRAC tools can also process datasets in the BAM or SAM format (`http://samtools.sourceforge.net/SAM1.pdf`), which is supported by many popular sequence aligners and is now a standard in the field.
Parsing BAM and SAM was, in part, implemented using the pysam module, a Python wrapper for the csamtools interface (`http://code.google.com/p/pysam/`). The following command line shows some of the frequently used flags with Novoalign that we use to map reads from CRAC experiments to the *Saccharomyces cerevisiae* genome:

```
novoalign -f solexadata.gz -d yeast.novoindex -r Random > solexadata.novo
```

In this example we used Novoalign version 2.0.5 and this command produces an output file in the native Novoalign format. Novoalign can handle gzip compressed files, which is

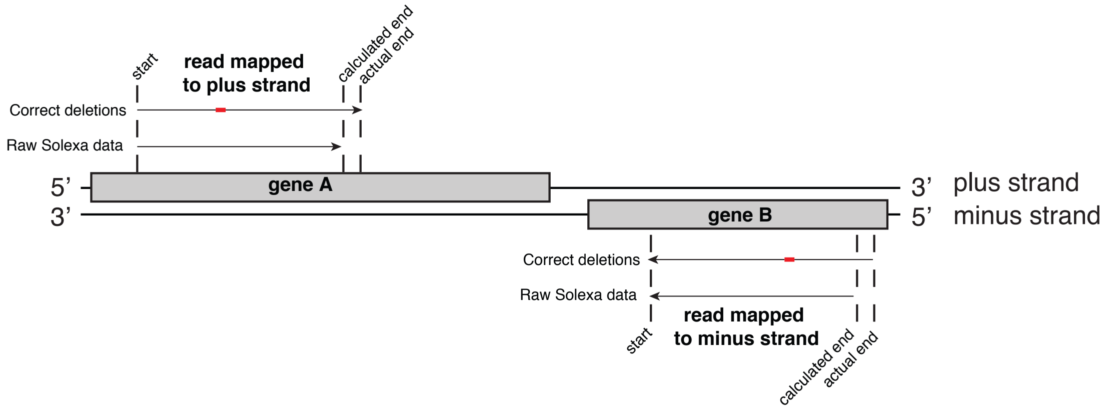

Figure 4.1: Schematic representation of how pyCRAC tools calculate chromosomal mapping positions using mutation information stored in novo or SAM/BAM files

a very useful feature. The novoindex file contains the indexed yeast genome. Reads that are mapped to multiple chromosomal positions will be randomly distributed over each position (-r Random flag). Because the pyCRAC Novoalign parser does not properly process soft-clipped reads, we would recommend not using the -o option. More detailed documentation on Novoalign and the Novoalign native output format can be found on the Novocraft website (www.novocraft.com).

```
novoalign -f solexadata.gz -d yeast.novoindex -r Random -o SAM > solexadata.sam
```

If you want Novoalign to output a SAM formatted file:

```
novoalign -f solexadata.gz -d yeast.novoindex -r Random -o SAM > solexadata.sam &
```

Note that in these examples we are only using a few default options and usually some tweaking is required to get optimal results.

4.4 Supported file formats

4.4.1 Input files must be tab-delimited

All of the input files, including the GTF feature files and the files containing the genomic sequences should be in tab-delimited format. If you would open any of the input files in a spreadsheet program, words or characters that are separated by tabs should be in separate columns, whereas characters separated by spaces should end up in a single column. Hence each tab separator essentially indicates the start of a new column. Do not edit these files

in spreadsheet programs as this can modify the file and cause problems with file parsing.

4.4.2 Processing and manipulating GTF feature files

The pyCRAC tools make heavy use of the Gene Transfer Format (GTF), a standardised tab-delimited text format describing genes and other chromosomal features. For more detailed information about GTF, see `http://mblab.wustl.edu/GTF22.html`, Table 4.1 and the explanation in Table 4.2. To process GTF feature files pyCRAC tools use the GTF2 parser, which is located in the pyCRAC installation folder. GTF2 reads the GTF file line by line and stores information into memory in a database-like format. For the smaller genomes, (i.e. bacterial and yeast), parsing only takes a few seconds, whereas the human GTF file takes about a minute and requires around 1GB of memory

Table 4.1: Example entries from a *Saccharomyces cerevisiae* GTF feature file. Each column indicates a separate field in the gtf file

seqname	source	feature	start	end	score	strand	frame	attributes
chrI	protein_coding	exon	83335	84474	.	-	.	gene_id "YAL032C"; transcript_name "YAL032C"; exon_number "1"; gene_name "PRP45"; transcript_name "PRP45";
chrI	protein_coding	CDS	83338	84474	.	-	0	gene_id "YAL032C"; transcript_name "YAL032C"; exon_number "1"; gene_name "PRP45"; transcript_name "PRP45"; protein_id "YAL032C";
chrI	protein_coding	start_codon	84472	84474	.	-	0	gene_id "YAL032C"; transcript_name "YAL032C"; exon_number "1"; gene_name "PRP45"; transcript_name "PRP45";
chrI	protein_coding	stop_codon	83335	83337	.	-	0	gene_id "YAL032C"; transcript_name "YAL032C"; exon_number "1"; gene_name "PRP45"; transcript_name "PRP45";

4.4.3 Support for other tabular annotation formats

The pyReadAligner.py and pyPileup.py tools also support a simpler tab-delimited text format that offers the users more flexibility. This is particularly useful if you want to quickly generate a multiple sequence alignment or pileup of a region that is not annotated. A line from such a tab-delimited input file is shown below:

```
RDN37-1 chrXII 451576 458433 -
```

Column 1 indicates the name of the feature, in this case a gene name, column 2 indicates the chromosome on which the feature is located, columns 3 and 4 indicate the start and end position of the feature, respectively and column five indicates on which strand the feature is located. If you want to generate a similar file, make sure that you do this in a simple text editor or in the terminal and that the entries are separated with tabs.

Table 4.2: Meaning of each individual column in GTF files

Column	Name	Explanation
1	$< seqname >$	Indicates the name of the chromosome/sequence on which the feature is located.
2	$< source >$	Indicates where the annotations came from. Can be an experiment the name of a program or database. Other source names are ”ncRNA”, ”snRNA” and ”rRNA”. PyCRAC tools refer to the entries in this column as ”annotations”.
3	$< feature >$	”CDS” indicates coding sequence coordinates. Exon coordinates include stop codons and often also UTR coordinates. ”CDS”, ”start_codon”, ”stop_codon” are essential features. ”exon”, ”5UTR” and ”3UTR” features are optional. Most GTF files from ENSEMBL do not contain UTR information. Exons are broadly defined as ANY transcribed exon and exon boundaries can include transcription start sites and splice sites. Hence UTRs can also be included in exon coordinates.
4	$< start >$	Indicates the start position of the feature on the chromosome (1-based).
5	$< end >$	Indicates the end position of the feature on the chromosome.
6	$< score >$	At this position you would normally find a score indicating the degree of confidence in the presence of the feature at the indicated positions. The GTF2 parser ignores this column.
7	$< frame >$	Indicates the frame in which the first nucleotide of a codon is present. ”0” means that the first nucleotide of the sequence is the first nucleotide of the codon.
8	$< strand >$	Indicates the strand; ”+”is the top strand, whereas ”-” indicates the bottom strand.
9	$< attributes >$	Indicates the attributes of the feature, separated by a space. The ”gene_name” attribute is essential and should be included in every file. The GTF2 parser requires both the ”gene_name” and the ”transcript_name”.

4.4.4 Chromosomal sequence files also need to be in tab-delimited format

PyCRAC tools load all the genomic annotations and the entire genomic sequence in memory. We found that this was the fastest way to analyse large datasets. When using the human genome, this requires several GB of RAM. Make sure that you have at least 8GB of RAM when doing analyses with human sequences or genomes of similar size. The file containing genomic sequences also need to be in tab-delimited format. In this format genomic sequences appear to be loaded much faster into memory compared to the standard fasta format. Genomic sequences in fasta format can be obtained from UCSC (`http://genome.ucsc.edu/`) or ENSEMBL (`http://www.ensembl.org/info/`

`data/ftp/index.html`). To convert the fasta file to the tab-delimited format you can use the pyFasta2tab.py script supplied with the pyCRAC package (see section 6.1.2) or FASTA-to-Tabular converter in Galaxy.

IMPORTANT! Make sure that the genomic sequence file has same version number as your GTF feature file and that the chromosome names in the .tab file are identical to the chromosome names in the GTF file!!! Otherwise the programs will not find any overlap between reads and genomic features. If the .tab and GTF files were obtained from the same source, then this is usually not a problem.

4.4.5 Novoalign and BAM/SAM formats

Novoalign native and SAM are tab-delimited text formats used for storing read mapping data (see `http://samtools.sourceforge.net/SAM1.pdf` and `www.novocraft.com` for more details). BAM files are compressed SAM files. The pyCRAC tools fully support these formats and use two Python modules called Novoalign.py and SAM.py to process them.

4.4.6 Handling paired-end data sets

Both single-end (the cDNA was sequenced from 5' end) and paired-end (the cDNA was sequenced from both ends) Novoalign and BAM/SAM data can be processed with pyCRAC. PyCRAC calculates start and end positions of cDNAs using mapping coordinates from paired reads or a single read . **NOTE** the tools do not take into consideration exon junctions. By default, paired reads that map to the same chromosome more than a 1000 nucleotides apart from each other will be ignored, however, this setting can be changed using the --distance flag.

4.5 Data processing fundamentals

4.5.1 Common options

PyCRAC programs contain many common options; several file input options and tool-specific options. Table 4.3 summarises (command line) options used by many pyCRAC tools and provides a brief description of what they do. More details for some of these options will be provided below and in Chapter 5

Table 4.3: Overview and description of frequently used pyCRAC options. The "command" column indicates the string that needs to be added to the command line in order to use the option.

Utility	Command	Description
Set the number of mapped reads you want to have analysed	-m<br>--max	If you add -m 1000 to the command line only a 1000 mapped reads will be analysed
Filter by alignment/mapping quality	--alignmentquality	Novoalign format: The alignment quality is calculated as 10log10(1 P(Ai— R, G)), where P(Ai—R, G) is the probability of the alignment given the read and the genome.SAM format: MAPQ: MAPping Quality, phred-scaled posterior probability that the mapping position of this read is incorrect).
Filter by alignment score (Novoalign)	--mappingquality<br>--alignmentscore	The alignment score is 10log10(P(R—Ai)) where P(R — Ai) is the probability of the read sequence given the alignment location i.
5' and 3' UTR coordinates	-r<br>--range	Including -r or --range flag followed by an integer the user can manually set the length of UTR sequences.
Removing possible PCR duplicates	--blocks	All the pyCRAC tools have the option to remove read blocks, defined as reads with identical sequences that have the same genomic coordinates. Essentially the same as collapsing the data but provides some more flexibility when it comes to mutations.
Remove reads with multiple alignment locations	--unique	To select only reads that map to a single genomic location.
Filter by read length	-l<br>--length	Shorter reads often provide a higher resolution of the RNA binding sites. This option allows you to remove reads longer than the input value.
Minimal overlap between read and feature	--overlap	Overlap is defined as the number of nucleotides the read sequence needs to overlap with a feature or gene in the genome. Default is 1 nucleotide.
Filter mutations in reads	--mutations	Mutations are frequently highlighted by the presence of deletions and, occasionally, substitutions. Use this option to filter the reads for a specific type of mutations. Deletions for CRAC and T-C mutations for PAR-CLIP.

Continued on next page

```
GGTGGAGAGAGCCTAGGTGATCGTCAGAT      Raw Solexa data
GGTGGAGAGAG--cCTAGGTGATCGTCAGAT    Highlight mutations
GGTGGAGAGAGtcgCTAGGTGATCGTCAGAT    Correct mutations
```

Figure 4.2: Sequence correction and highlighting in pyCRAC

Table 4.3 – *Continued from previous page*

Utility	Command	Description
Genomic or coding reference sequence?	-s<br>--sequence	To specify if you want introns included in the pileups or multiple sequence alignments. Useful when studying RNA binding proteins that do not interact with introns.
Store the reads the programs discard in a text file	--discarded	Prints the reads that were filtered out by the programs into a separate text file, the name of which should be supplied after the --discarded option. Useful for quality control purposes.
Compress all output file into a zip file	--zip	You need to supply a file name after the --zip option with a .zip extension.

4.5.2 Read sequence correction

The Novoalign and BAM/SAM formats define the start position as the leftmost mapping of the first matching base of the read sequence on a chromosome or reference sequence. The pyCRAC tools calculate the end of the chromosomal mapping position of the read by adding the length of the read to the start position coordinate. However, CRAC data very frequently have deletions and therefore to obtain the correct end mapping position the number of nucleotides that are deleted must be included when calculating the mapping positions of the read (see Figure 4.1). The Novoalign and BAM/SAM format report locations of mismatches in mapped reads and pyCRAC tools automatically use this information to correct the read length and the read sequence. When using pyReadAligner to generate multiple sequence alignments, dashes are inserted at positions were deletions were found (see Figure 4.2) and substitutions are always highlighted as lowercase letters. RNA binding motifs extracted from CRAC/CLIP data frequently contain mutations. Therefore, it is very important to correct the sequence of reads with deletions when searching for enriched motifs in the data. The pyCRAC motif search program pyMotif.py does this automatically

Figure 4.3: Calculating overlap between read mapping positions and genomic features. An example showing the effects of changing the overlap setting in pyCRAC tools.

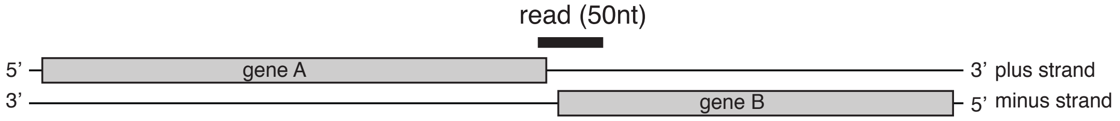

**Overlap setting:(nt)**	gene A	gene B
1	sense	anti-sense
25	-	anti-sense
50	-	-

4.5.3 Calculating overlap between reads and genomic features

CLIP/CRAC cDNA library preparation protocols generate directional libraries and therefore sequencing data will contain strand information. PyCRAC was designed to specifically tackle directional libraries and reports whether reads are sense and anti-sense to genomic features (Figure 4.3). By default, a read only has to overlap one nucleotide with a feature to be considered a hit. This setting can be changed in many pyCRAC tools by using the --overlap flag. Unless specified, feature chromosomal coordinates are calculated from exon coordinates (Figure 4.5). For an example of how the overlap function works, see Figure 4.3 and the table below it. In this example we have a 50 nucleotide read that was mapped to the top strand of a chromosome. This read partly overlaps with gene A on the plus strand and gene B on the bottom strand. When using the default settings, pyCRAC will include this read as a sense hit to gene A and an anti-sense hit to gene B. When overlap is set to 25 nucleotides, pyCRAC tools will only report an anti-sense hit to gene B. If overlap is set to 50 nucleotides (i.e. 100 percent overlap is required), the read will not be considered a hit to either gene.

PyReadCounters, pyPileup and pyReadAligner also have an --ignorestrand flag that allow users to analyse datasets that do not contain strand information, such as ChIP-seq data.

4.5.4 Alignment qualities and alignment scores

A read can be aligned to single or multiple locations in the genome and this information is used to calculate an alignment quality (Novoalign) or mapping quality (MAPQ in the SAM format). Dealing with alignment qualities can get quite complicated as the actual value depends on the algorithm used by the sequence aligner. The pyCRAC tools allow you to remove reads with poor alignment quality/mapping quality by setting a threshold. Reads with qualities lower than the threshold will be ignored. By default, pyCRAC tools do not take alignment/mapping qualities into consideration and we would

recommend using the default settings if you just started using pyCRAC to analyse a dataset. More detailed information about alignment and mapping quality calculations can be found in the Novoalign and SAM documentation (`www.novocraft.com`, `http://samtools.sourceforge.net/SAM1.pdf`). Novoalign also uses an alignment score, which according to the Novoalign documentation is defined as: *10log10(P(R—Ai)) where P(R — Ai) is the probability of the read sequence given the alignment location i. A threshold of 75 would allow for alignment of reads with two mismatches at high quality base positions plus one or two mismatches at low quality positions or to ambiguous characters in the reference sequence.*

PyCRAC tools also allow you to set a threshold for the alignment scores. Reads with alignment scores lower than the threshold will be ignored.

4.5.5 How to deal with untranslated (UTRs) and flanking regions and how manually set their coordinates

The GTF "exon" feature is broadly defined as "all transcribed regions" and exon boundaries can include transcription start sites, splice sites and poly-adenylation sites (see Figure 4.5). Therefore, exon features can also contain 5' and 3' untranslated regions (UTRs). "CDS" features refer to translated nucleotide sequences, or coding sequences, which includes the start codon. The GTF2 parser automatically extracts any UTR information from GTF files by comparing "exon" and "CDS" coordinates, as outlined in Figure 4.5 and includes this information when looking for overlap between reads and genomic features. Many GTF files, however, do not contain information about transcription start sites and/or poly-adenylation sites. Coordinates for untranslated regions are available from BioMart (ENSEMBL) and/or the UCSC Table browser. The *Saccharomyces cerevisiae* database (SGD) also stores GFF files containing UTR coordinates. These, however, need to be converted to the GTF format in order to work with pyCRAC.

The simplest way to include new UTR coordinates in pyCRAC data analyses is to add them to the GTF feature files, either as new entries flanking exon features or by indicating them as separate UTR features. When including UTR coordinates as exon features, pyCRAC tools will automatically assume these are UTR sequences if accurate CDS coordinates are also present. These will automatically be included in the data analyses. **NOTE**: if no CDS coordinates exist for the gene of interest then no UTR coordinates will be calculated. In these cases it is best to add the UTRs as separate features. An example of how to add UTR coordinates to GTF annotation files is shown in Table 4.4. In the command line example shown below the pyReadCounters.py program looks for overlap between yeast genomic genes/transcript coordinates from the GTF file and read

Figure 4.4: Example showing how to add UTR coordinates to GTF annotation files. UTR coordinates are indicated as exons or UTRs

```
chrXVI  protein_coding  CDS  802355  804076  .  +  0   gene_id "YPR137W"; gene_name "YPR137W";
chrXVI  protein_coding  exon  802355  804076  .  +  0   gene_id "YPR137W"; gene_name "YPR137W";
chrXVI  protein_coding  stop_codon  804074  804076  .  +  0   gene_id "YPR137W"; gene_name "YPR137W";
chrXVI  protein_coding  exon  802310  802354  .  +  0   gene_id "YPR137W"; gene_name "YPR137W";
chrXVI  protein_coding  exon  804074  804254  .  +  0   gene_id "YPR137W"; gene_name "YPR137W";
        OR:

chrXVI  protein_coding  5UTR  802310  802354  .  +  0   gene_id "YPR137W"; gene_name "YPR137W";
chrXVI  protein_coding  3UTR  804074  804252  .  +  0   gene_id "YPR137W"; gene_name "YPR137W";
```

coordinates in the SolexaData.sam file. The program generates several output files (detailed in Chapter 5) including a table containing a list of genes and a hittable for each genomic feature in the GTF file. Using the -r or --range flag it is possible add sequences to the 5' and 3' ends of genes to determine if proteins interact with flanking regions. For protein coding genes this option can be used to manually set a fixed length for both 5' and 3' UTR coordinates for all genes/transcripts but also to add flanking regions to non-coding RNAs (see Figure 4.5).

```
pyReadCounters.py -f SolexaData.sam --file_type=sam --gtf=yeast.gtf -r 300
```

Figure 4.5: The GTF2 parser calculates 5 and 3 UTR coordinates by comparing start and end positions of "exon" and "CDS" features. **NOTE** that the stop codon is not included in CDS features. UTR coordinates can also be included as separate features, indicated as 5UTR and 3UTR, respectively. TSS indicates the transcriptional start site, whereas pA indicates the poly-adenylation site. Red arrows indicate 300 nucleotide long 5 and 3 UTR sequences.

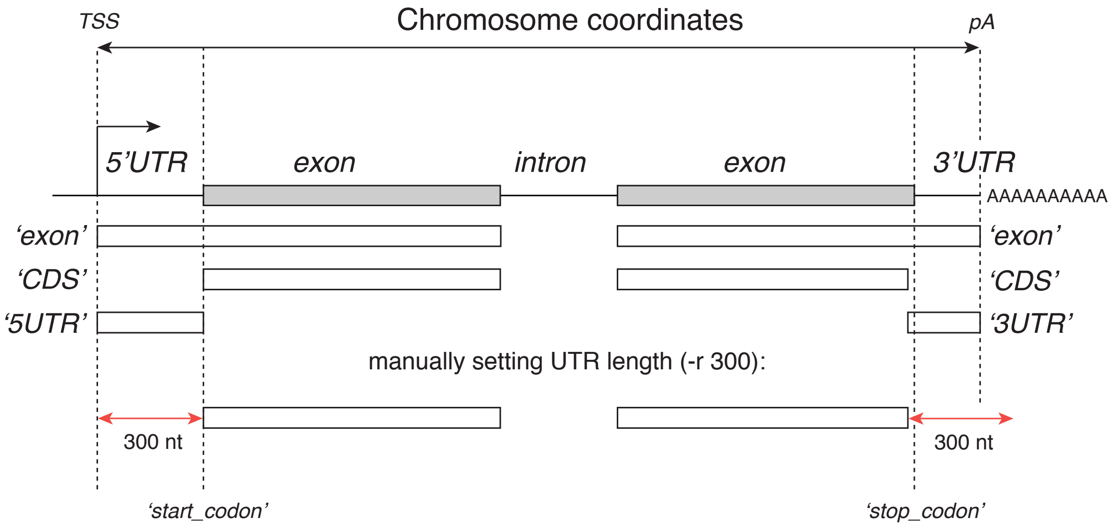

Table 4.4: Explanation of the meaning of the terms reads, cDNAs, blocks and clusters

Term	Explanation
reads	Reads are ALL the sequences in a FASTQ or Novoalign/BAM/SAM data file.
blocks	Reads with identical nucleotide sequences and chromosomal mapping positions.
cDNAs	Indicate unique sequences. A library can contain 50000 unique cDNA sequences but hundreds of reads can have the same cDNA sequence.
clusters	Assemblies of at least two overlapping cDNA sequences. Blocks are treated as a single cDNA during cluster analysis.

4.5.6 Reads, cDNAs, blocks, clusters and multiple alignment locations

Because UV cross-linking is very inefficient, we often have very little RNA to work with when generating cDNA libraries. Hence, CRAC cDNA libraries are frequently of low complexity, sometimes containing less than fifty-thousand unique cDNA sequences. Therefore, it is very likely that cDNAs are amplified many times during the PCR step and some sequences could be preferentially amplified. This bias gives a false positive impression of the actual number of hits for a particular feature or gene. When visualising CRAC data in genome browsers, these potential PCR duplicates often appear as large "blocks" or "read stacks" (see Figure 4.6 and Table 4.4). We now routinely incorporate 3-6 random nucleotides in adapter barcode sequences to assess the degree of PCR duplication. Barcoded raw data is first demultiplexed using pyBarcodeFilter.py and then collapsed using pyFastqDuplicateRemover.py. This is described in more detail in section 5.1
The --blocks flag in pyCRAC can also be used to collapse your data, however, this function works on Novo, BAM and SAM files. The --blocks filter uses both read chromosomal mapping positions and mutations to determine if a read is a duplicate: reads that have the same chromosomal mapping coordinates but have deletions/substitutions at different positions will **NOT** be considered PCR duplicates.
We have also included a tool that generates clusters from read data (pyClusterReads). Clusters are defined as an assembly of at least two overlapping reads and can provide an indication of the number of RNA binding sites present in an RNA. For more details about clusters, see section 5.3.1.
To use the blocks option simply add --blocks to the command line:

```
pyReadCounters.py -f SolexaData.sam --file_type=sam --gtf=yeast.gtf --blocks
```

Figure 4.6: Examples showing removal of putative PCR duplicates (blocks) and cluster generation. Shown is a schematic representation of a gene (*YFG1*) containing two exons and one intron. Reads and clusters are indicated as thick black lines. Mutations are indicated as asterisks. (A) All reads that mapped to *YFG1* are displayed. (B) PCR duplicates or "blocks" are condensed into one cDNA sequence using pyFastqDuplicateRemover. Note that positions of mutations are considered when removing duplicates, however, reads with the same coordinates are still counted as a single cDNA during cluster formation. (C) Clusters generated from at least two overlapping cDNA sequences using pyClusterReads. This step removes the reads forming the large block in the second exon of the gene. (D) Clusters generated from at least five unique cDNAs. This removes the cDNA sequences mapped to the 5' region of the gene.

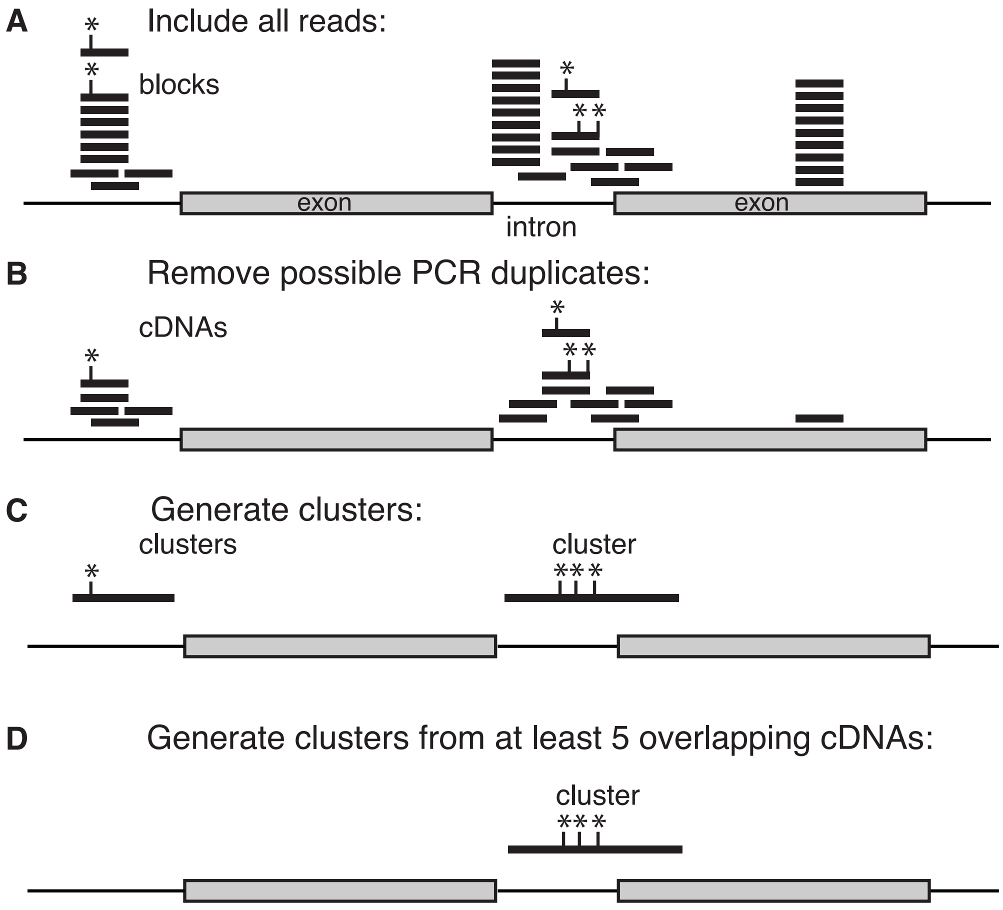

4.5.7 Using genomic and coding sequences as reference

The pyCRAC package contains three tools, pyPileup, pyReadAligner and pyBinCollector, that allow you to look at distribution of reads on genomic features. If you are performing CLIP/CRAC on a protein that does not interact with introns then it is sometimes useful to be able to exclude intron sequences from data analyses, particularly when analysing sequencing data from experiments performed in higher eukaryotes. Human genes, for example, generally have relatively short exons and very long introns. When -s coding or --sequence=coding is included in the command line, pileups or alignments will be generated using only coding sequences (CDS). For more details about this option, see the sections in Chapter 5 discussing pyPileup.py, pyReadAligner.py and pyBinCollector.

4.5.8 Filtering the data for reads with mutations

Because deletions in pyCRAC data frequently highlight the protein cross-linking site they play very important role in the data analyses. Many pyCRAC tools have an option called --mutations, which allows you to filter the mutations present in reads or clusters (see section 5.3.1). By default the program keeps track of all the mutations but it can be instructed to only consider deletions (--mutations=delsonly), reads that have substitutions (--mutations=subsonly). Finally, one can also select for specific nucleotide substitutions. Cross-linking sites in PAR-CLIP data are often indicated by T-C conversions. By using the --mutations=TC only T-C mutations are stored. This option can be used in combination with almost any other option.

Usage example: We want pyReadCounters.py only to analyse reads mapped to a single genomic locations and only deletions are reported:

```
pyReadCounters.py -f SolexaData.sam --file_type=sam --gtf=yeast.gtf --unique
    --mutations=delsonly
```

4.5.9 Additional common options

The -m or --max options allow you to specify how many mapped reads you want to have analysed. The programs automatically ignore reads that failed quality control (QC) or unmapped (NM). This option is useful for normalising your data to compare different datasets. The -l or --length flags allow you to set a maximum read length threshold. For example, when you add -l 20 to the command line then reads longer than 20 nucleotides will be ignored. Shorter reads give rise to narrower peaks and therefore higher-resolution

RNA binding sites. To set the maximum number of base-pairs allowed between two non-overlapping paired reads we included the -d or --distance flag, which can only be used when analysing paired-end data. The default setting is 1000 nucleotides. At this setting paired reads mapped to the same chromosome but separated by more than 1000 nucleotides apart will be discarded. The --discarded option prints the discarded reads to an output file. An output file name needs to be entered after this flag. This will allow you to determine how stringent your filtering settings are.

4.5.10 File handling options

The most common file handling flags are -f or --input_file for loading read mapping data (Novoalign or BAM/SAM format (for pyReadCounters and pyPileup tools)), the --gtf flag for GTF feature files and the --tab flag for handling tab-delimited genomic sequence files. Chapter 5 also introduces the -g and --chr flags for indicating gene lists and other tab-delimited files. **NOTE,** by default, all pyCRAC tools use the *Saccharomyces cerevisiae* GTF feature and genomic sequence tab files in the */usr/local/pyCRAC/db* folder. Hence, if the --gtf and/or --tab flags are not included in the command line, the programs assumed the read sequences are derived from yeast.

Chapter 5

The pyCRAC tools

This Chapter describes the functionality of the main pyCRAC tools. To better convey the purpose of the tool we in some cases included several example figures. We also discuss frequently used option and provide numerous command line examples.

5.1 pyBarcodeFilter

The throughput of sequencing applications has increased tremendously over the past few years. At the time of writing, a single lane on an Illumina HiSeq machine routinely generated up to 180 million single-end reads. For most CRAC experiments involving small genomes a few million reads is often sufficient. We multiplex our samples using barcoded 5' adapters that contain random nucleotides. This allows a better assessment of number of PCR duplicates. There are numerous programs available that can demultiplex samples, however, to our knowledge there isn't a publicly available tool that can tackle random barcodes. PyBarcodeFilter.py tool can process barcodes containing random nucleotides and also barcodes of different lengths. Other unique features are that the tool can process paired end data and gzip-compressed input files. It can also compress output files. The tool looks for barcodes in 5' ends of reads and generates separate data files for each barcode in a minimal FASTQ format. Once the tool identifies a barcode, the barcode sequence and corresponding quality characters are removed from the raw data and placed in a separate output file. Reads without recognisable barcodes are also reported. The program also generates output files containing some statistics about barcodes and random nucleotides, when present. Recently I made a significant update to pyBarcodeFilter and now it can also handle barcodes with random nucleotides at multiple positions within the barcodes sequence.

When pyBarcodeFilter encounters a barcode with random nucleotide sequences it will

Figure 5.1: Example of a header after splitting randomly-barcoded data with pyBarcodeFilter. If the barcode sequence file indicates barcodes with random nucleotides, the tool will remove the barcode and attach the random barcode sequence (red) to the header with two hashes (blue).

```
FCC0TU2ACXX:4:1101:1968:2135#ACAGTGAT1##GTTCTC
```

Table 5.1: Example of a barcode text file

NNNCGCTTAGCNN	mutant2
NNNGCGCAGCNN	mutant1
NNNATTAGNN	control
NNNTAAGCNN	myfavprotein

store the random nucleotide sequence in the header of the read, as shown in figure ??.
NOTE After demultiplexing the data with pyBarcodeFilter we generally collapse the data to remove any potential PCR duplicates. This can be done using the fastx_collapser tool from the fastx toolkit (`http://hannonlab.cshl.edu/fastx_toolkit/`); however we have also included additional scripts, including pyFastqDuplicateRemover, that can deal with random barcodes. This is discussed in detail in section 6.2.1.

5.1.1 Usage and option summary

To get the help menu for pyBarcodeFilter type pyBarcodeFilter.py -h in the terminal. This tool requires FASTA or FASTQ input files containing the raw data and a text file containing barcode information. To process paired end data, use -f and the -r flags to indicate the path to the forward and reverse sequencing reactions, respectively. The barcodes file should two columns separated by a tab (see Table 5.1). The first column should contain the barcode nucleotide sequences. The second column should contain an identifier, for example, the name of the barcode or the name of the experiment. The 'N' in the barcode sequence indicates a random nucleotide. Make sure to use a simple text editor like TextEdit (MacOS X), gedit (Linux/Unix) or use a text editor in the terminal. The program is case sensitive: all the nucleotide sequences should be upper case. You can freely combine different barcodes but if you are mixing samples containing random nucleotide barcodes and normal barcodes, make sure to place the regular barcode sequence below the sequence with random nucleotides and make sure the shortest sequence is **ALWAYS** at the bottom in the column, as shown in Table 5.1.

NOTE! pyBarcodeFilter always expects the random barcode sequence to be at the 5' end and or 3' end of the barcode sequence and it does not allow mixing of adapter sequences

with random barcodes of different lengths! In this case it is better to do multiple sequential demultiplexing runs. For example: in the past only the following would be accepted:

NNNATGC

The new program can now deal with three types of in-read barcodes:

NNNATGC
ATGCNNN
NNNNATGCNN

NOTE! It is essential that you do **NOT** mix these different type of in-read barcodes and the stretches of random nucleotides should always be of the same length!

Accepted:

NNNATGCNN sample1
NNNTGACNN sample2
NNNAGATNN sample3

A barcode file with the following sequence will produce errors as they are mixed:

NNNATGCNN sample1
NTGCANNN sample2
TGCANNN sample3

The -m or --mismatches option sets the number of allowed mismatches in a barcode. The maximum is one mismatch. We use RNA barcode sequences in our linkers and sometimes these are trimmed at the 3 ends by one or two nucleotides. Setting the number of allowed mismatches to 1 may reveal more barcoded sequences. **Use with caution!!** When using short barcodes (as shown in Table 5.1) we would recommend using default settings for allowed number of mismatches (0).
Usage example for processing paired-end data:

```
pyBarcodeFilter.py -f data_1.fastq -r data_2.fastq b barcodes.txt
```

pyBarcodeFilter can also process gzip compressed input files:

```
pyBarcodeFilter.py --file_type=fasta.gz -f data_1.fasta.gz -b barcodes.txt
```

And compress output files using gzip (-9 compression):

```
pyBarcodeFilter.py --file_type=fasta.gz -f data_1.fasta.gz -b barcodes.txt --gzip
```

5.1.2 Output files

Besides generating separate FASTQ or FASTA files for barcoded reads it also produces text files containing useful barcode and random nucleotide statistics.

5.2 pyReadCounters

After aligning the reads to the reference genome the first thing we routinely do is generate hit tables describing the number of reads that mapped to genes or transcripts and to which features they were mapped (UTRs, introns, exons, CDS, etc). We also want to visualise our data in genome browsers to look at the hit distribution on chromosomes. How many unique cDNAs do you really have in the data? How many reads map anti-sense to genes? This basically summarises what pyReadCounters.py does. The program takes the Novoalign or BAM/SAM file and asks which reads overlap with genomic features from a GTF file.

5.2.1 Usage and option summary

To access the pyReadCounters help menu, type pyReadCounters.py -h in the terminal. This tool supports a large number of the common options. These are discussed in detail in Chapter 4. The file input and pyReadCounters specific options will be discussed below.

5.2.2 Default behaviour

CRAC data are usually not very complex, particularly when the bait protein has only few RNA binding sites or RNA substrates. Because of this, pyCRAC tools quite often first collapse the data by counting the reads that contain the exact same cDNA sequence. From this, the smallest possible number of unique cDNA sequences is determined, and only cDNA mapping coordinates are compared to genomic features in the GTF feature files. If a cDNA overlaps with a feature then the number of hits for that feature is incremented by the number of reads with the same mapping position. This makes downstream

manipulations, such finding overlap with genomic features and sorting of the intervals, very easy. However, the analysis of very large and complex datasets (10 GB or higher) may require a lot of RAM memory.

By default pyReadCounters produces three output files: (1) A text file containing information about the complexity of the sequencing data, (2) a GTF file containing all the genomic mapping positions of cDNA sequences and overlapping genomic features (both sense and anti-sense) and (3) a hit table text file that shows for each genomic feature the number of intervals that mapped sense or anti-sense. Counts for biotypes (such as protein_coding, tRNA, snRNA, etc) are also included. PyReadCounters supports novoalign native format, SAM/BAM and also GTF files. One can run pyReadCounters to generate a read data GTF file, modify the GTF file and re-run pyReadCounters on the modified GTF file to generate a new hit table. PyReadCounters also requires a GTF feature file. Use the -f option to indicate the path to the Novoalign, BAM/SAM or GTF interval file. Use the --gtf option to indicate the path to the GTF feature file.

5.2.3 Command line examples

Running pyReadCounters using default settings:

```
pyReadCounters.py -f SolexaData.novo --gtf=yeast.gtf
```

In the last example all the required files were in the working directory. To use files located in a different folder you need to include the entire file path. For example:

```
pyReadCounters.py -f /usr/SeqData/210211/SolexaData.novo
    --gtf=/usr/local/pyCRAC/db/yeast.gtf
```

By default, all pyCRAC programs assume that the file containing the aligned reads is in the native Novoalign format (i.e. --file_type="novo"). To use a BAM or SAM file as input file you need to specify the file type (case sensitive) in the command line:

```
pyReadCounters.py -f SolexaData.bam --file_type=sam --gtf=yeast.gtf
```

One can also use a pyReadCounters GTF file as input file, in case you need to re-count the number of hits for features after modifying the GTF file. To use a GTF file as input

file the --file_type needs to be used:

```
pyReadCounters.py -f modifiedreadcountersdata.gtf --file_type=gtf --gtf=yeast.gtf
```

Or if you want to count overlap between clusters and genomic features:

```
pyReadCounters.py -f myclusters.gtf --file_type=gtf --gtf=yeast.gtf
```

To remove reads with poor alignment quality and print out the discarded reads:

```
pyReadCounters.py -f SolexaData.novo --gtf=yeast.gtf --align_quality=50
    --discarded=discarded_reads.txt
```

Note that this only works with novo or SAM/BAM files.
Use the --ignorestrand flag to analyse ChIPseq or RIPseq data:

```
pyReadCounters.py -f SolexaData.novo --gtf=yeast.gtf -c 1 --ignorestrand
```

To compress all the output files into a single zip archive use the --zip flag followed by a file name with a .zip extension:

```
pyReadCounters.py -f SolexaData.novo --gtf=yeast.gtf --align_quality=50
    --discarded=discarded_reads.txt --zip=outputs.zip
```

By default, pyReadCounters reports all substitutions and deletions in the GTF output files it generates. However, if you are analysing PAR-CLIP data, you may only be interested in looking at T to C conversions. If you have CLIP or CRAC data, you may only be interested in deletions. Using the –mutations flag you can instruct pyReadCounters to report only specific mutations.
For example:

```
pyReadCounters.py -f SolexaData.novo --gtf=yeast.gtf --mutations=delsonly
pyReadCounters.py -f MyPAR_ClipData.novo --gtf=yeast.gtf --mutations=TC
```

Using the --rpkm flag, pyReadCounters will also calculate for the reads that mapped sense to each genomic feature the RPKM, which is defined as the number of reads per kilobase transcript per million mapped reads. If you are analysing paired-end data, pyReadCounters will count fragments, not individual reads. Hence the reported RPKM values are actually FPKMs (Fragments per kilobase transcript per million mapped reads)
For example:

```
pyReadCounters.py -f SolexaData.novo --gtf=yeast.gtf --rpkm
```

The pyCRAC package has received a number of major updates since version 1.2. We have introduced a large number of new features. By default pyReadCounters considers genomic features that map both sense and antisense to reads/cDNAs. However, using the --sense and --anti_sense flags, users can now instruct pyReadCounters to consider only sense or antisense overlapping features. For example:

```
pyReadCounters.py -f SolexaData.novo --gtf=yeast.gtf --sense
pyReadCounters.py -f SolexaData.novo --gtf=yeast.gtf --anti_sense
```

This command instructs pyReadCounters to only count reads/cDNAs that overlap 'sense' with genomic features.
Since version 1.2.2 you can also ask pyReadCounters to count reads mapped to introns, exons, 5'UTRs or 3'UTRs using the -s or --sequence flag. This only works with novo or SAM-BAM files. For example:

```
pyReadCounters.py -f SolexaData.novo --gtf=yeast.gtf --sense -s intron --rpkm
pyReadCounters.py -f SolexaData.novo --gtf=yeast.gtf -s exon --rpkm
pyReadCounters.py -f SolexaData.novo --gtf=yeast.gtf -s 5UTR --rpkm
```

Using the -a or --annotation flag you can instruct pyReadCounters to only count specific features such as protein_coding or tRNAs.

For example:

```
pyReadCounters.py -f SolexaData.novo --gtf=yeast.gtf --sense -s exon --unique
    --rpkm -a protein_coding
```

PyReadCounters will then only count reads or fragments that overlap with exons, they must be unique cDNA sequences and map to protein_coding genes. RPKM values will also be calculated.
NOTE! This will only work if "protein_coding" is used in the "source" column to annotate protein coding genes (see Table 4.1).

Last but not least, a more complicated example that demonstrates the flexibility of pyReadCounters: For example:

```
pyReadCounters.py -f SolexaData.novo --gtf=yeast.gtf --sense -s exon --unique
    --rpkm -a protein_coding --mutations=delsonly --align_quality=50
```

This command generates output files in which unique cDNAs that map sense to exons of protein_coding genes are reported. RPKMs are included in the hit table and the GTF output files will only show positions of deletions within the cDNAs.

5.2.4 Output files

The file_statistics.txt file provides the cumulative number of reads for each unique cDNA sequence in the data. When plotting the file_statistics.txt data one would hope to see more or less a straight line (see Figure 5.2), indicating a strong linear relationship between the cumulative number of reads and the number of unique cDNA sequences. In this situation, most cDNA sequences are generally equally represented in the sequencing data. Very high complexity cDNA libraries are characterised not only by a high number of unique cDNA sequences, but also each cDNA sequence is represented by only a few reads. If the line resembles a logarithmic curve then this indicates that some cDNA sequences are highly overrepresented. These libraries are frequently of low complexity. The hit tables provide an overview of the genomic features and the number of reads that were mapped to each feature. Because CRAC data contains strand information, pyReadCounters calculates the number of reads mapped sense and anti-sense to each feature. Figure 5.3 shows a few lines from a pyReadCounters hittable file or RNASeq data.

To generate the hit table pyReadCounters searches for overlap between read/clusters and genes in GTF annotation files, which include all transcribed sequences (see section 4.2 for more details). Because a single read can overlap with multiple features, the total number of hits can exceed the total number of reads. The hit table also shows how many

Figure 5.2: Example plots indicating high-and low-complexity datasets

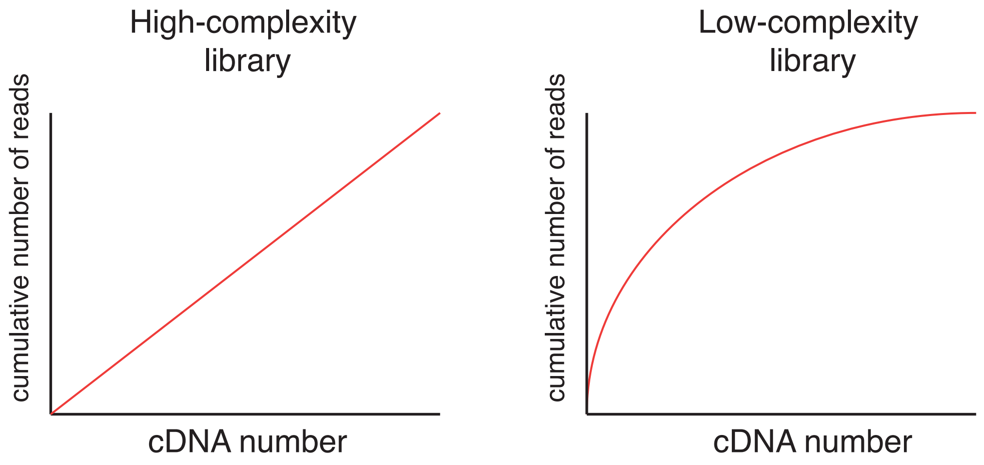

Figure 5.3: Example of a pyReadCounters hittable output file

```
# generated by pyReadCounters version 1.1.0, Mon Apr 16 20:34:22 2012
# /usr/local/bin/pyReadCounters.py -f RNAseq_data.novo -c 1 --unique
# total number of reads 12534556
# total number of paired reads  10947376
# total number of single reads  483095
# total number of mapped reads: 11430471
# total number of overlapping genomic features  7019550
#       sense   5960669
#       anti-sense      1058881
# feature       sense_overlap anti-sense_overlap  number of reads

## protein_coding       3190701
YEF3        49930       3629        24221
PMA1        32621       2650        21776
COX1        24559       1037        15174
TFP1        21539       1689        13506
HSC82       21177       1458        12729
ADH1        20245       1467        11351
AI5_ALPHA   20022       918         13101
AI4         19390       886         12638
AI3         17823       798         11473
AI2         17590       790         11297
RPL10       16822       1113        8797
ENO2        16336       1125        8913
TEF1        15578       1333        5450
```

Figure 5.4: Example of a pyReadCounters cDNAs GTF output file

```
##gff-version 2
# generated by Counters version 1.2.0, Tue Jan  8 22:47:29 2013
# pyReadCounters.py -f PAR_CLIP_unique.novo --mutations=TC -v
# total number of reads: 2455251
# total number of paired reads: 0
# total number of single reads: 2455251
# total number of mapped reads: 2455251
# total number of overlapping genomic features: 5153943
# sense: 2640600
# anti-sense: 2513343
chrXIV reads exon 661572 661605 2 + .
gene_id "INT_0_6716,YNR016C"; gene_name "INT_0_6716,ACC1"; # 661596S;
chrXIV reads exon 661720 661738 1 + .
gene_id "INT_0_6716,YNR016C"; gene_name "INT_0_6716,ACC1"; # 661726S;
chrXIV reads exon 661839 661878 4 + .
gene_id "INT_0_6716,YNR016C"; gene_name "INT_0_6716,ACC1"; # 661875S;
```

were mapped to biotypes or annotations (column 2 in the GTF feature file), which are indicated by two hashes in the hittable file. In this example, over three million reads were mapped to protein_coding genes. The first column shows the feature names, which are either the "gene_name" or "gene_id" names from the GTF feature file. The second and third columns show the number of sense and anti-sense reads, respectively, that overlapped with the gene. The fourth column shows the number of unique cDNA sequences that overlap with the feature.

PyReadCounters also generates a GTF formatted output file containing all the extracted sequence intervals and overlapping genomic features. Using the GTF format makes pyCRAC compatible with many existing tools, such as BEDTools and genome browsers. An example of a pyReadCounters GTF output file: The pyReadCounters GTF format differs slightly from the GTF2 format specifications (see section 4.4.2) and is sorted by chromosome, strand and then read start position. Column 2 in pyReadCounters file contains "reads" or "cDNAs" if the --blocks option was used. Column 6, or the "score" column in pyReadCounters GTF files indicates the number of reads mapped to chromosomal start and end positions shown in columns 4 and 5, respectively. The "gene_id" and "gene_name" attributes in column 9 shows the GTF file features that overlap with cDNA coordinates. Multiple overlapping features are shown as single "gene_id" and "gene_name" entries and are separated by commas. **NOTE!** These include sense AND anti-sense overlapping features. A "no matches" indicates that the cDNA did not overlap with annotated features. Furthermore, pyReadCounters GTF files also indicate 0-based chromosomal mapping positions of mutations in cDNAs which are indicated with a hash.

For example, "# 661596S" indicates that in the cDNA sequence there is a substitution at chromosomal position 661596.

PyReadCounters also produces a GTF file for reads that overlap (sense) to annotated UTR and intron coordinates. The results are printed in the intron_and_UTR_overlap.gtf file. **IMPORTANT!** 5' and 3' UTR sequences in GTF feature files can be included as 'exon' features (see Table 4.4). In these cases reads that map to UTR regions will also be counted as exon hits.

The pyReadCounters GTF output file can be used with the bedtools genomecoverageBed or the pyGTF2bedGraph.py script to generate bedGraph files, a format that is frequently used to display read coverage over chromosomes. For more details about making bedGraph files, please see section 6.3.4. We have also included a script in pyCRAC that converts the GTF output into bed6 files (see section 6.3.3).

Sometimes you need to rerun pyReadCounters and you don't want to wait until it has generated all three output files. Since version 1.2 pyReadCounters now has three additional flags (--hittable, --stats and --gtffile) that allows you to select which output file you want the tool to generate:

```
pyReadCounters.py -f Solexadata.novo --gtf=yeast.gtf --stats
pyReadCounters.py -f Solexadata.novo --gtf=yeast.gtf --hittable
pyReadCounters.py -f Solexadata.novo --gtf=yeast.gtf --gtffile
```

5.3 pyClusterReads

5.3.1 Default behaviour

We routinely generate read clusters from the sequencing data. Clusters are defined as assemblies of at least two overlapping cDNA sequences. **NOTE!** that the term cDNA sequences should not be confused with reads (see Figure 4.6 and Table 4.4). For example, a block consists of a single cDNA sequence but can include hundreds of reads. To generate clusters, pyClusterReads will look for read entries that overlap by at least one nucleotide. One can manually set the maximum cluster length, the minimal required overlap between a read and a cluster and the data can also be filtered for total number of overlapping reads or the maximum "height" of the cluster. Clustering your data essentially flattens the data by assembling overlapping cDNAs into a single nucleotide sequence. Cluster-

ing removes biases in sequence representation caused by PCR and abundantly expressed sequences that have a high read coverage. It also removes noise or spikes generated by isolated reads or read blocks (see Figure 4.6). Clusters assembled from a large number of overlapping cDNAs likely represent *bona fide* RNA binding sites, and hence the number of clusters overlapping a gene/transcript provides an indication of the number of RNA binding sites. We also use cluster data to get an impression of the reproducibility of CRAC experiments, by looking at overlap between clusters in different data sets. This clustering feature is in many aspects similar to the mergeBed tool provided in BEDTools, which combines overlapping features into a single feature. One of the advantages is that it is a very flexible tool, with several cluster filtering steps that are very straightforward to use.

NOTE! for pyClusterReads to work properly, the GTF input file has to be sorted by chromosome, then by strand and then by start position. The pyReadCounters tool sorts the read intervals for you so if you did not modify the file in any way it should work without any problems. If you are not sure whether your file is correctly sorted, you can run the following sort command line on your pyReadCounters GTF file:

```
sort -k1,1 -k7,7 -k4,4n reads.gtf > reads_sorted.gtf
```

We have also included a script that calculates False Discovery Rates for genomic regions (pyCalculateFDRs.py, see section 5.7). This tool produces a GTF output file that provides a good starting point for generating clusters over significantly enriched regions.

5.3.2 Output files

This tool expects pyReadCounters GTF files as input files and generates a GTF output file. All cluster intervals are also annotated with overlapping genomic features. Mutation frequencies for cluster nucleotides are included as comments in the GTF output file. This should make it simpler to isolate clusters that have nucleotides with high mutation frequencies. If no overlapping features were found, gene_names and gene_ids will be "no_matches". Figure 5.5 shows a few lines from a pyClusterReads GTF output file.

The pyClusterReads GTF output file essentially has the same layout as other pyCRAC GTF output files. The maximum height of the cluster is indicated in the "frame" column (8). The hash character at the end of each line (#) shows chromosomal coordinates of mutated nucleotides within the cluster interval and their mutation frequencies. For example, # 114099S100.0 indicates that 100% of the nucleotides in position 114099 were

Figure 5.5: A few lines from a pyClusterReads GTF output file

```
##gff-version 2
# generated by pyClusterReads.py version 0.0.1, Fri Jan 18 11:59:42 2013
# pyClusterReads.py -f count_output_reads.gtf -o count_output_clusters.gtf -v
# chromosome    feature source  start   end     cDNAs   strand  height  attributes
chrI    cluster exon    112583  112643  6       -       5
gene_id "INT_0_114,YAL021C"; gene_name "INT_0_114,CCR4"; # 112612S75.0;
chrI    cluster exon    113176  113232  3       -       3
gene_id "INT_0_114,YAL021C"; gene_name "INT_0_114,CCR4"; # 113184S100.0;
chrI    cluster exon    113334  113386  2       -       2
gene_id "INT_0_114,YAL021C"; gene_name "INT_0_114,CCR4"; # 113349S50.0,113379S100.0;
chrI    cluster exon    113534  113564  3       -       3
gene_id "INT_0_119,INT_0_114"; gene_name "INT_0_119,INT_0_114";
# 113554S33.3,113556S33.3,113557S33.3;
chrI    cluster exon    113644  113691  5       -       4
gene_id "YAL020C,INT_0_114"; gene_name "ATS1,INT_0_114";
# 113649S50.0,113657S33.3,113679S25.0
chrI    cluster exon    113912  113958  2       -       2
gene_id "YAL020C,INT_0_114"; gene_name "ATS1,INT_0_114";
# 113932S50.0,113946S50.0;
chrI    cluster exon    113966  114066  5       -       3
gene_id "YAL020C,INT_0_114"; gene_name "ATS1,INT_0_114";
# 113987S50.0,114033S33.3,114039S33.3;
chrI    cluster exon    114067  114130  3       -       3
gene_id "YAL020C,INT_0_114"; gene_name "ATS1,INT_0_114"; # 114099S100.0;
```

substituted. By default, the tool prints to the standard output in the terminal.

5.3.3 Command line examples

Running pyClusterReads at default settings:

```
pyClusterReads.py -f SolexaData_reads.gtf --gtf=yeast.gtf -o
    SolexaData_clusters.gtf
```

In case you only want to generate clusters for protein coding genes:

```
pyClusterReads.py -f SolexaData_reads.gtf --gtf=yeast.gtf -a protein_coding -o
    SolexaData_mRNA_clusters.gtf
```

NOTE! This will only work if "protein_coding" is used in the "source" column to annotate protein coding genes (see Table 4.1).

What if you want to look at clusters that map to sequences flanking genes? Or your GTF annotation file does not contain any UTR information and you are interested in determining if there are any clusters just up or downstream of coding sequences? For these things you can use the -r or --range flag, which adds flanking sequences to all annotated features. An example:

```
pyClusterReads.py -f SolexaData_reads.gtf --gtf=yeast.gtf -a protein_coding -r
    200 -o SolexaData_mRNA_clusters_flank200.gtf
```

This adds 200 nucleotides to 5' and 3' end of all protein coding sequences (CDS) or exons if CDS coordinates are not available.

PyClusterReads also allows the user to set a minimal number of overlapping cDNA sequences in clusters using the --cic or --cdnasinclusters flag (see Table 4.3).
For example:

```
pyClusterReads.py -f SolexaData_reads.gtf --gtf=yeast.gtf --cic=5 -o
    SolexaData_clusters.gtf
```

At default clustering settings, a single nucleotide overlap is sufficient to form a cluster or to join a cluster. This setting can be changed using the cluster overlap option (--co or --clusteroverlap). This allows you to select for clusters with sharp peaks. An example:

```
pyClusterReads.py -f SolexaData_reads.gtf --gtf=yeast.gtf --cic=5 --co=10 -o
    SolexaData_clusters.gtf
```

You can also set a minimum peak hight for a cluster using the --ch or --clusterheight flag:

```
pyClusterReads.py -f SolexaData_reads.gtf --gtf=yeast.gtf --ch=10 -o
    SolexaData_clusters.gtf
```

By default the maximum length of a cluster is set to 100 nucleotides. This setting can be changed using the --cl or --clusterlength flag. Once it reaches the maximum allowed length, it simply starts a new cluster. An example:

```
pyClusterReads.py -f SolexaData_reads.gtf --gtf=yeast.gtf --ch=10 --cl=50 -o
    SolexaData_clusters.gtf
```

You can also instruct the tool to only report nucleotide positions that have a specific mutation frequency:

```
pyClusterReads.py -f SolexaData_reads.gtf --gtf=yeast.gtf --cic=5 --co=10
    --mutsfreq=20 -o SolexaData_clusters.gtf
```

In this example only nucleotide positions that are mutated in at least 20 percent of the clustered reads are reported.

5.4 pyPileup and pyReadAligner

The genome browser is a very useful tool to visualise read distribution on chromosomes; however, often it is more convenient to generate separate pileup files or multiple sequence alignments for individual genes or chromosomal regions. Many tools exist that can generate pileups and alignments, including HTSeq and samtools; however, pyPileup

and pyReadAligner offer features that are not available in these packages, including the option to select reads based on the presence of mutations and making coverage plots for exons only. Both tools can be used for analysing non-directional data, such as ChIPseq using the --ignorestrand flag and pyPileup can also process GTF formatted files.

5.4.1 Usage and option summary

PyPileup and pyReadAligner have almost identical options. As with all other pyCRAC tools, to view the help menu with pyPileup or pyReadAligner, use the -h flag.

5.4.2 Default behaviour

PyPileup and pyReadAligner require four input files: (1) a file containing the mapping data (-f option), (2) a GTF feature file (--gtf flag), (3) a genomic reference sequence file in the tab format (--tab) and (4) a text file containing information about the genes/-transcripts or chromosomal regions of interest (-g or --chr options). If the --gtf or --tab options are not provided; the tools will use the default yeast annotation and genomic sequence files. For BAM/SAM/GTF input files, the file type also needs to be included in the command line (i.e. --file_type=sam or --file_type=gtf). Two types of gene/transcripts files are supported. The first type contains a single column with gene names or transcript names, or if these are not available, gene ids or transcript ids. Both programs automatically detect gene or transcript names. Use the -g flag to input single column gene/transcript list files in pyPileup and pyReadAligner. **NOTE!** The names provided in a gene list file should be identical to the ones provided in the GTF file and no additional spaces or empty lines should be present in the file. By default pyPileup and pyReadAligner analyse **ALL** of the mapped reads in your data and this can take some time; however, for multiple sequence alignments it is rarely necessary to more than a few thousand reads, particularly when a large number of reads mapped to your genes of interest. For pyPileup, a hundred thousand to one million reads is usually more than enough (-m 100000). For pyReadAligner several thousand is usually sufficient (-m 1000). Both tools also provide a --limit flag that allows you to set a maximum for the number cDNAs mapped to gene/transcript you want to have reported.
Example of a gene/transcript list file:

```
RDN37-1
SNR17A
YOR078C
RPL7A
```

Both programs also accept the tab-delimited format described in section 4.4.3 in combination with the --chr flag. Example:

```
RDN37-1    chrXII    451576    458433    -
```

NOTE! We strongly advise users to use a simple text editor like TextEdit (OS X), gedit (Linux) or Notepad (Windows) or command line text editors such as VIM and nano to make these files. Avoid using Microsoft office or equivalent as these programs tend to introduce unwanted new line characters.

5.4.3 Output files

The pyPileup output format normally contains six columns in which each line consists of the gene or feature name, the 1-based coordinate, the reference base, the number of reads that cover that base, the number of times the reference base was substituted in reads and the number of times the reference base was deleted in reads. PyPileup generates separate output files for sense and anti-sense reads. PyReadAligner reports multiple sequence alignments in a fasta format that can be viewed by programs like SeaView or BioEdit. We have also included the pyAlignment2Tab.py script to convert pyReadAligner fasta files into a tab-delimited file (see section 6.1.1).

5.4.4 Command line examples

Many of the common pyCRAC options (see section 4.5.1) can be used with pyReadAligner and pyPileup. A few of these are discussed below and command line examples are provided.
Usage examples:

```
pyPileup.py -f SolexaData.novo --gtf=yeast.gtf --tab=yeast.tab -g geneslist.txt
    --limit=5000
```

```
pyReadAligner.py -f SolexaData.novo --tab=yeast.tab --limit=5000
    --chr=featurelist.txt
```

Note that in the second example a gtf annotation file is not required because the coordinates for the genes of interest were included in the featurelist.txt file.
Two major changes have been introduced in version 1.2.2. First, pyPileup and pyReadAligner

now only report reads that overlap sense with genes of interest. To generate plots or alignments for reads mapped anti-sense to features, you need to include the --anti_sense flag:

```
pyPileup.py -f SolexaData.novo --gtf=yeast.gtf --tab=yeast.tab -g geneslist.txt
    --anti_sense -o anti_sense_hits
pyReadAligner.py -f SolexaData.novo --gtf=yeast.gtf --tab=yeast.tab -g
    geneslist.txt --anti_sense -o anti_sense_alignment
```

pyPileup can also report counts for 5' or 3' ends of reads (--5end and --3end flags). This can be useful if you are analysing iCLIP data (Note that versions 1.2.2 and above no longer have --iCLIP flag!)
For example:

```
pyPileup.py -f SolexaData.novo --gtf=yeast.gtf --tab=yeast.tab -g geneslist.txt
    --5end
pyPileup.py -f SolexaData.novo --gtf=yeast.gtf --tab=yeast.tab -g geneslist.txt
    --3end
```

To make multiple sequence alignments containing 1000 reads with deletions for each gene/transcript in the gene list:

```
pyReadAligner.py -f SolexaData.novo --gtf=yeast.gtf --tab=yeast.tab -g
    geneslist.txt --limit=1000 --mutations=delsonly
```

To find cross-linking sites in PAR-CLIP data you can choose to make an alignment only from cDNAs that have T-C conversions:

```
pyReadAligner.py -f PAR-CLIP_data.bam --file_type=sam --gtf=yeast.gtf
    --tab=yeast.tab -g geneslist.txt --limit=1000 --mutations=TC
```

To include 100 bp of sequence upstream and downstream of gene/transcript chromosomal coordinates use the -r option. **NOTE!** When using the -r flag any 5UTR and 3UTR entries in the GTF annotation file will be ignored. An example:

```
pyReadAligner.py -f SolexaData.novo --gtf=yeast.gtf --tab=yeast.tab -g
    geneslist.txt --limit=1000 -r 100
```

Figure 5.6: The pyPileup -s coding flag can be used to remove intron sequences. Shown are two plots displaying the read distribution over a gene called *YFG1*. In the right panel, only the hits that mapped to exons are displayed.

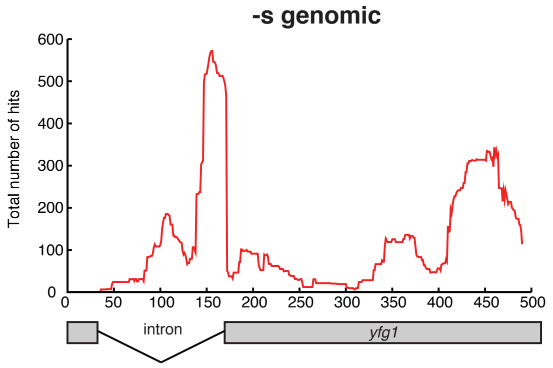

Both pyPileup and pyReadAligner provide the option to generate pileups/alignments using genomic (default) or coding reference sequences (-s or --sequence). By default genomic reference sequences are used, which include UTR and intron sequences. To limit the analysis to exons, include the command -s coding or --sequence=coding (see example in Figure 5.6). The latter can be particularly useful if your genes/transcripts have a large number of (long) introns. PyCRAC does not yet properly deal with reads overlapping exon junctions, so these may be excluded from the output. This will be improved in future versions of pyCRAC.
A more complex example of pyCRAC usage illustrating the flexibility of pyCRAC:

```
pyPileup.py -f SolexaData.novo --gtf=yeast.gtf --tab=yeast.tab -g geneslist.txt
    --blocks --align_quality=40 -s coding --discarded=discarded.txt
    --zip=output.zip
```

This command generates pileups for genes/transcripts listed in the geneslist.txt file. Only cDNAs that map to coding sequences and that have an align or mapping quality of at least 40 are considered. Discarded reads are printed to the "discarded.txt" file and all the output files are compressed as "output.zip".

5.5 pyMotif

5.5.1 Motif search algorithm

PyMotif extracts short sequences from large datasets to identify potential RNA binding motifs. **It is important to note that PyMotif only extracts k-mers from reads**

that map to genomic features. PyMotif compares the results with a control dataset, which it generates by extracting k-mers from reads randomly distributed over the same genomic features. For each k-mer the program calculates a standard score or Z-score that indicates overrepresentation of the k-mer sequence in the experimental data, and is defined as the number of standard deviations by which an actual k-mer count minus the k-mer count from random data exceeds zero. PyMotif is not as sophisticated as popular programs such as MEME but an advantage of using pyMotif for motif analyses is that it is very flexible and it can process thousands of intervals fairly quickly. Many different settings can therefore be tested in a relatively short time and the data should provide a good basis for more detailed motif analyses. The tool produces a data-rich, but human friendly, GTF formatted output file that can be further processed by many different tools.

NOTE!! We would strongly recommend not to process more than 20.000 intervals so it is best to run pyMotif on the most significant peaks or clusters. Do not run pyMotif on read intervals as this does not produce relevant results and will take too long to process.

5.5.2 Usage and option summary

To access the pyMotif help menu, type pyMotif.py -h in the terminal. Many of the common options are supported in pyMotif. These are discussed in section 4.5.1. The file input and pyMotif specific options will be discussed below.

5.5.3 Default behaviour

PyMotif expects a GTF file as input file. Normally we use pyCalculateFDRs and pyClusterReads GTF output files as input files, as this reduces sequence overrepresentation biases introduced by highly expressed genes. PyMotif requires three input files: (1) the file containing the interval data in GTF format (-f flag), (2) a GTF feature file (--gtf flag) and (3) a file containing the genomic sequences in tab-delimited format (--tab option). The GTF feature file is required to extract reads that overlap with genomic features. **NOTE** If a particular k-mer sequence is repeated many times in a sequence, it will only be counted once, as the k-mer search results would otherwise be biased towards homo-polymeric sequences.

Table 5.2: Overview of output files generated by pyMotif

**Outputfile**	**Description**
random_k-mers_count.txt	Contains a list of k-mer sequences and k-mer frequencies in the random dataset.
dataset_k-mers_count.txt	Contains a list of k-mer sequences and k-mer frequencies in the experimental dataset.
k-mer_Z_scores.txt	Contains a list of k-mer sequences, Z-scores and mutation frequencies.
top_k-mers_in_genes.gtf	A GTF file containing k-mer chromosomal locations, k-mer sequences, strand and overlapping features.

5.5.4 pyMotif-specific options

The --k_min and --k_max flags allow you to set the range of k-mer sequence lengths to be extracted from the data. By default the k-mer length is 4 to 8 nucleotides. A very large dataset may have tens of thousands unique k-mer sequences, but quite often only the top 100 k-mers are of interest. Use the -n or --numberofkmers option to set the maximum number of unique k-mer sequences you want to have reported. As stated previously, pyMotif performs k-mer analyses on reads, cDNAs or clusters that map to genomic features described in a GTF file. Any sequences that are mapped anti-sense to genomic features will also be included. To ensure that all sequences are included, annotations for intergenic regions should be added to the GTF file. Using the -a or --annotation option, you can instruct pyMotif to focus on reads mapped to genomic features with a specific source name or annotation (column 2 in the GTF feature file). By default pyMotif includes all annotations. If you are not sure what annotations are available to you, you can run the pyGetGTFSources.py script on your GTF feature file to extract these (see section 6.3.6).

5.5.5 Output files

Figure 5.7 shows an example of a k-mer_Z_scores.txt file. In this example pyMotif analysed clusters assembled from at least 10 cDNA sequence that map to protein_coding genes. The TGTAG sequence is clearly enriched in clustered reads, indicating that the protein of interest has a preference for binding RNAs with UGUAG motifs. Figure 5.8 shows a few lines from a pyMotif GTF output file. In the pyMotif GTF output file columns 3 shows the k-mer sequences and column 6 contains k-mer Z-scores. The attributes in column 9 show the gene names or genomic features in which the k-mer sequence was found. Column 7 indicates the k-mer strand. **NOTE!** Because pyMotif includes reads mapped anti-sense to genomic features, it is possible that the k-mer sequence is located on the opposite strand from the genomic feature(s) shown in column 9.

Figure 5.7: A Few lines from a k-mer_Z_scores.txt file generated by pyMotif. The first column shows the k-mer sequence, the second column the Z-score for that motif and the third column shows the mutation frequency, which indicates the percentage of motifs that have at least one mutation in the sequence.

```
# pyMotif.py -f PAR_CLIP_unique_count_output_clusters.gtf --file_type=gtf -v
# Thu Oct 11 11:46:40 2012
# k-mer lengths:
#    min: 4
#    max: 8
# 8107 clusters

# k-mer     Z-score
TGTAG       42.70
TGTA        38.38
GTAG        34.01
TTGTA       31.21
TGTAGA      28.53
TGTAA       28.09
TTGTAG      28.02
CTGTA       27.58
TGTAGT      26.00
GTAGT       24.88
GTAGA       24.57
ATGTA       22.71
```

5.5.6 Command line examples

Using pyMotif at default settings:

```
pyMotif.py -f PAR_CLIP_clusters.gtf --gtf=yeast.gtf --tab=yeast.tab
```

Restricting the number of k-mers in output files:

```
pyMotif.py -f PAR_CLIP_clusters.gtf --gtf=yeast.gtf --tab=yeast.tab
    --numberofkmers=100
```

What if you want to limit the motif search to protein coding genes?

```
pyMotif.py -f PAR_CLIP_clusters.gtf --gtf=yeast.gtf --tab=yeast.tab -a
    protein_coding
```

Or you want to look for motifs in flanking sequences but your GTF annotation files has

Figure 5.8: A Few lines from a pyMotif 'top_k-mers_in_features' GTF file.

```
##gff-version 2
# pyMotif.py -f PAR_CLIP_data_clusters.gtf -v
# Thu Oct 11 11:46:42 2012
# k-mer lengths:
#    min: 4
#    max: 8
# total number of unique k-mers reported:    1000
# total number of reads:    1061285
# total number paired reads:    0
# total number single reads:    850707
# total number of mapped clusters:    8107
# chromosome source  sequence start   end      Z-score     strand   .
attributes
2-micron    motif    TGTAG    3370    3374    42.6971678483    +    .
gene_id "R0030W"; gene_name "RAF1";
2-micron    motif    TGTAG    3564    3568    42.6971678483    +    .
gene_id "R0030W"; gene_name "RAF1";
2-micron    motif    TGTAG    3990    3994    42.6971678483    +    .
gene_id "INT_0_4"; gene_name "INT_0_4";
2-micron    motif    TGTAG    4351    4355    42.6971678483    +    .
gene_id "INT_0_4"; gene_name "INT_0_4";
2-micron    motif    TGTAG    4715    4719    42.6971678483    +    .
gene_id "INT_0_4"; gene_name "INT_0_4";
2-micron    motif    TGTAG    5837    5841    42.6971678483    +    .
gene_id "R0040C"; gene_name "REP2";
```

no UTR information:

```
pyMotif.py -f PAR_CLIP_clusters.gtf --gtf=yeast.gtf --tab=yeast.tab -a
    protein_coding -r 200
```

The -r 200 flag will add 200 nucleotide flanking sequence to each protein coding feature in the GTF file. **NOTE!** This flag tells the GTF parser to ignore any 5UTR or 3UTR information in the GTF annotation file.

5.6 pyBinCollector

PyBinCollector can be used to generated coverage plots for analysing the genome-wide distribution of reads or clusters over genomic features. To do this it normalises gene or feature lengths by dividing their sequences into equal number of bins. At default settings it calculates nucleotide densities that map to each bin, which we define as the total number of nucleotides from reads that map to the bin. To generate distribution profiles for individual genes, we have included --outputall flag. This produces tab-delimited files for sequences, substitutions and deletions and include all the genes of interest (i.e. protein_coding, snoRNAs,etc), which allows the user to generate heat maps and box plots. PyBinCollector accepts any pyCRAC GTF file as input file.

5.6.1 Usage and option summary

To access the help menu, type pyBinCollector.py -h in the terminal. Many of the common options can be used with pyBinCollector. These are discussed in detail in Chapter 4. Below we discuss the pyBinCollector-specific options and two common options.

5.6.2 Default behaviour

pyBinCollector requires two input files: (1) a pyReadCounters or pyMotif GTF file (-f flag) and (2) a GTF feature file (--gtf flag). It is also possible use a pyReadCounters or pyMotif GTF file as an annotation file (see below). Like many of the other pyCRAC tools, pyBinCollector gives you the option to focus your analysis on genomic features that associated with specific sources or annotations (column 2 in the GTF feature file). Sequences not mapped to genomic features will be ignored. By default pyBinCollector looks at all annotations; however, you can also instruct the program to look specifically in protein coding genes (e.g. -a protein_coding). If you do not know what annotations

are available to you, you can run the pyGetGTFSources.py script on your GTF feature file to extract these (see Table 1.2).
Usage example:

```
pyBinCollector -f SolexaDataCTTG.gtf --gtf=Yeast.gtf -a protein_coding -n 50 -o
    motif_distribution.pileup
```

In this example we used the CTTG motif GTF file shown in Figure 5.8 as input file and we wanted to analyse the distribution of this motif over all protein coding genes (-a protein_coding). Gene lengths were divided into 50 bins.
By default, the program will only report motifs that overlap sense with protein_coding features, however, using the --substitutions or --deletions flags, mutations can also be reported. When you include the --anti_sense flag in the command line, only reads that mapped antisense to features will be considered.
Usage examples:

```
pyBinCollector -f SolexaDataCTTG.gtf --gtf=Yeast.gtf -a protein_coding -n 50 -o
    motif_distribution.pileup --substitutions
```

```
pyBinCollector -f SolexaDataCTTG.gtf --gtf=Yeast.gtf -a protein_coding -n 50 -o
    motif_distribution.pileup --deletions
```

```
pyBinCollector -f SolexaDataCTTG.gtf --gtf=Yeast.gtf -a protein_coding -n 50 -o
    motif_distribution.pileup --deletions --anti_sense
```

To look at the distribution of the CTTG motif over all the protein coding genes individually, one can add the --outputall flag:

```
pyBinCollector -f SolexaDataCTTG.gtf --gtf=Yeast.gtf -a protein_coding -n 50
    --outputall
```

Note that when using the outputall flag you do not have include an output file name.
Although pyBinCollector is very useful for binning features, if you leave out the -n flag from the previous command line example, then it will produce a large table with read,

substitution or deletion densities for the entire gene, without binning. This can be very useful if you want to look at the distribution of your reads/clusters transcriptome-wide.

Usage example:

```
pyBinCollector -f SolexaDataCTTG.gtf --gtf=Yeast.gtf -a protein_coding --outputall
```

Because there can be a lot of variation in read density over different genes, we would recommend adding the --normalize flag in combination with the --outputall flag. This simply divides the total number of reads covering each nucleotide position or bin by the total number of reads covering the gene or feature:

```
pyBinCollector -f SolexaDataCTTG.gtf --gtf=Yeast.gtf -a protein_coding
    --outputall --normalize
```

5.6.3 Output files

At default settings pyBinCollector prints hit densities in each bin to the standard output and generates a pyBinCollector log text file. To print to a dedicated file name use the -o flag. An example pileup file is shown in Figure 5.9. By default, pyBinCollector will divide all protein coding gene sequences into 20 bins but 50 to 100 bins usually gives satisfactory results. Very high bin numbers will ultimately increase processing times. Also, if pyBinCollector encounters genes that are shorter than the total number of bins, it will not include these in the analyses and report this in the pyBinCollector log file. Figure 5.10 shows a section of a pyBinCollector pileup file generated using the --outputall flag. This output file is compatible with Cluster 3 (`http://bonsai.hgc.jp/~mdehoon/software/cluster/software.htm#ctv`), which can be used to cluster the data. Cluster 3 output files are compatible with Java TreeView (`http://jtreeview.sourceforge.net`), allowing graphical representation of the clustering results.

5.6.4 Command line examples

PyBinCollector also has a bedtool-like function that extracts intervals from the GTF file containing that map to specific features (--overlap) or specified bin numbers (--binoverlap).Consider the example discussed in section 5.6.2. Let's say we want to know

Figure 5.9: Shown is a section of a pyBinCollector pileup file generated using the --outputall flag. Gene names are listed in the first column and each following column shows the read densities for each bin.

```
# generated by BinCollector, Sun Mar 16 13:37:48 2014
# pyBinCollector.py -f /usr/local/pyCRAC/tests/test_count_output_reads.gtf -n 20 -o test_dist.tx
# bin   hits_or_fraction
1       7607.0
2       8466.0
3       7755.0
4       8538.0
5       8428.0
6       8218.0
7       8693.0
8       9199.0
9       9464.0
10      9787.0
11      10197.0
12      9285.0
13      9423.0
14      10808.0
15      13055.0
16      8688.0
17      8685.0
18      7279.0
19      7602.0
20      8591.0
```

Figure 5.10: Section of a pyBinCollector pileup file generated by including the --outputall flag. Gene names are listed in the first column and each following column shows the read densities for each bin.

```
AAR2        0        6        6        5        4        0        0
AAT1        0        0        0        0        0        0        0
AAT2        0        0        0        0        0        0        0
ABD1        0        0        0        1        2        3        3
ABZ1        0        0        0        0        0        0        0
ACB1        0        0        0        0        0        0        0
ACC1        0        0        0        0        0        4        0
ACE2        0        0        0        0        0        0        0
ACS1        0        0        0        1        2        2        1
ACS2        0        0        0        0        0        0        0
ADE3        0        0        4        5        5        6        6
ADE4        0        0        0        7        1        0        0....
....
....
```

which motifs overlap with 5'UTRs of mRNA coding genes:

```
pyBinCollector -f SolexaDataCTTG.gtf --gtf=Yeast.gtf --overlap -s 5UTR -a
    protein_coding -o motif_intervals_in_5UTR.gtf
```

What about introns?

```
pyBinCollector -f SolexaDataCTTG.gtf --gtf=Yeast.gtf --overlap -s intron -a
    protein_coding -o motif_intervals_in_introns.gtf
```

What if I want to plot the distribution of all CTTG motifs throughout mRNA coding genes without binning the features? For this you use the --outputall flag. This generates a large file with CTTG nucleotide densities for individual genes.

```
pyBinCollector -f SolexaDataCTTG.gtf --gtf=Yeast.gtf --outputall -a
    protein_coding -o CTTG_distribution_over_mRNAs.txt
```

Using the --normalize flag, for each gene it will divide the nucleotide densities for each position by the total nucleotide densities.

```
pyBinCollector -f SolexaDataCTTG.gtf --gtf=Yeast.gtf --outputall -a
    protein_coding -o CTTG_distribution_over_mRNAs.txt --normalize
```

I suspect that my protein only binds to mature mRNAs, so I do not need to have the intron sequences included:

```
pyBinCollector -f SolexaDataCTTG.gtf --gtf=Yeast.gtf --outputall -s coding -a
    protein_coding -o CTTG_distribution_over_mRNAs.txt --normalize
```

We now want to know which protein coding genes have a CTTG motif at or near the 3' end. We want to divide all gene sequences into 50 bins and ask pyBinCollector to extract those intervals from the GTF file that mapped to the last five bins:

```
pyBinCollector -f SolexaDataCTTG.gtf --gtf=Yeast.gtf -n 50 --binoverlap 45 50 -o
    motifs_in_3prime_end.gtf
```

The --binoverlap flag expects two numbers, separated by a space. A threshold for the sequence length of genomic features can also be set. In the example below pyBinCollector will only consider genomic features that are between 500 and 1000 nucleotides long:

```
pyBinCollector -f SolexaDataCTTG.gtf --gtf=Yeast.gtf -n 50 --binoverlap 45 50
    --min_length=500 --max_length=1000 -o motifs_in_3prime_end.gtf
```

In the following example we used pyClusterReads.py to generate a clusters GTF output file and we would like to know which clusters overlap with 5' ends of protein coding genes. Then we want to identify overrepresented sequences in these clusters. Below we used the -r 200 flag to add 200 nucleotide flanking sequences to protein coding genes.

```
pyBinCollector -f clusters.gtf --gtf=Yeast.gtf -r 200 -n 50 --binselect 1 4 -o
    clusters_in_5end.gtf
```

Alternatively, one could only select those clusters that overlap with the 200 nucleotide upstream region by using the -s 5UTR flag:

```
pyBinCollector -f clusters.gtf --gtf=Yeast.gtf -r 200 -n 50 -s 5UTR --binselect 1
    4 -o clusters_in_5UTR.gtf
```

These GTF files can then be used to perform a motif analysis:

```
pyMotif.py -f clusters_in_5end.gtf
pyMotif.py -f clusters_in_5UTR.gtf
```

We often compare pyMotif results with data produced by other motif finding programs, like the MEME suite (`http://meme.sdsc.edu/`). Almost all of these programs require a fasta formatted input file. To generate a fasta file from the selected clusters, we use the bedtools getfasta script. Make sure to include the -s option to force strandedness.

```
bedtools getfasta -bed clusters_in_5UTR.gtf -fi yeast.fasta -s -fo
    clusters_in_5UTR.fasta
```

Figure 5.11: Distribution of deletions in and around the CUUG motif identified in Nab3 CRAC data

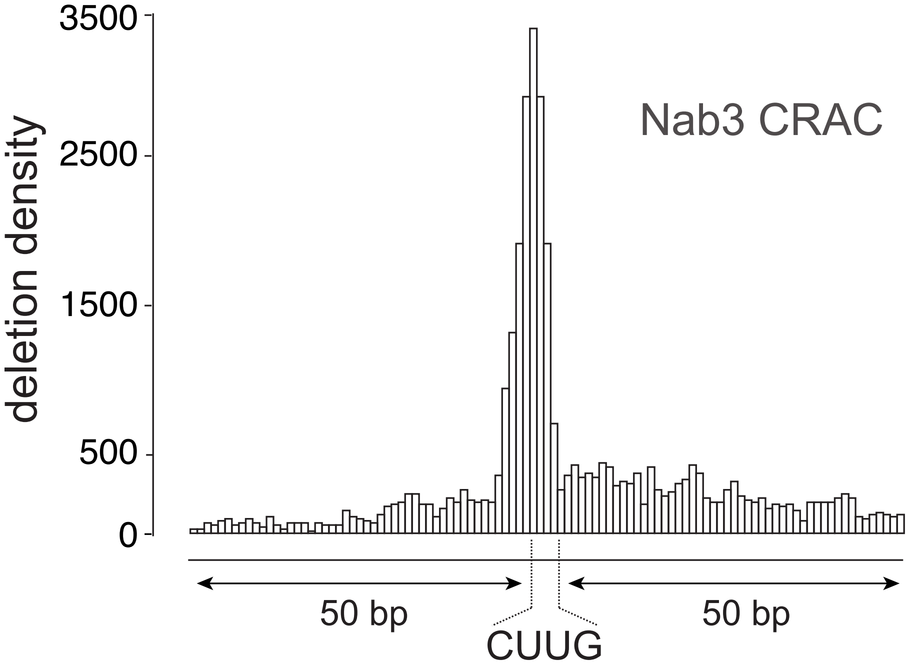

To run pyMotif to on the GTF file. Again we include the -r 100 flag to make sure that pyMotif includes clusters overlapping the 100 nt UTR sequences:

```
pyMotif.py -f SolexaData_selectedbins.gtf --gtf=yeast.gtf --tab=yeast.tab -r 100
```

Finally, pyBinCollector also has the --outputall flag that Complicated example:
The pyBinCollector program also prints out the distribution of deletions and substitutions over genomic features. We used pyBinCollector to calculate the distribution of deletions at and around the CTTG motifs present in protein coding genes. An example is shown in Figure 5.11. To generate this result we used the following command line:

```
pyBinCollector.py -f Clusters_count_output.gtf --gtf=SolexaDataCTTG.gtf -n 104 -r
    50 --max_length=104 -s exon
```

Explanation: The motifs were generated from clusters. To get the location of all the

mutations in clusters, we use a clusters gtf file as input file. As a gtf annotation file we no longer use the standard yeast one but the SolexaDataCTTG.gtf file. Each CTTG containing motif will then be associated with a gene name. The GTF2 parser does not recognise CTTG as a feature (remember it only knows 'exon','CDS','5UTR' and '3UTR') so it automatically assumes it is an 'exon' feature and adds the genomic mapping coordinates to the exon feature database. Genes in the database can have multiple exon coordinates because they can contain more than one CTTG motif. To calculate the distribution over all possible 'exon' coordinates, we use the -s exon flag. If we would use the default option (i.e. -s genomic), it would treat multiple exon features as a single gene and calculate gene start and end positions from the coordinates. PyBinCollector can do the same for introns (-s intron) and coding sequence coordinates (-s CDS). The -r flag adds 50 nucleotides to each side of the CTTG motif found in each gene. Because we only want to look at 4-mers and not longer motifs containing CTTG, we set the maximum length to 104 (=50+50+4). We then divide these sequences over a 104 bins, meaning that each bin will contain a single nucleotide. The CTTG sequence will be divided over bins 51 to 54. The graph shows that deletions in clusters are indeed enriched over the CTTG motif; strongly suggesting that CUUG is a bonafide RNA binding motif.

5.7 pyCalculateFDRs

Ideally with each CLIP experiment we would like to include many controls, however, sometimes it is difficult to generate sufficient PCR product for negative control samples for sequencing, making it difficult to assess the background level. To tackle this problem, Fred Gage's lab devised an algorithm that allows calculation of False Discovery Rates (FDRs) for CLIP data lacking negative controls (see `http://www.nature.com/nsmb/journal/v16/n2/full/nsmb.1545.html`). If a genomic region with a certain read coverage has an FDR of 0.01, then this indicates that there is a one in hundred probability that this region is not significantly enriched (i.e false positive). This approach is a good statistical method to further analyse your data, however, short genes with high read coverage will less frequently have low FDR values. Such features are tRNAs, snoRNAs and perhaps miRNAs. In these cases it might be worth adding flanking sequences using the -r flag.
A similar algorithm was later incorporated into Pyicos (`http://regulatorygenomics.upf.edu/group/media/pyicos_docs/`) and we have included a slightly modified version of this algorithm in pyCalculateFDR.py. The script requires read coordinates in the bed6, GTF or GFF format and a GTF annotation file. The user sets an FDR threshold (default = 0.05) and the program calculates for each genomic feature the minimum coverage height required to obtain an FDR lower or equal than the threshold. It generates a GTF output

file that shows the genomic regions with an FDR lower or equal to the set threshold (see Figure 5.12). It also generates a log file containing for each feature information about coverage heights and FDR values. The algorithm takes all the reads that map to a genomic feature and randomly distributes these over the same feature several times (default = 100 iterations). It then compares the results with the actual coverage. For a given coverage height (h) it calculates the probability of finding this coverage in the data as follows:

$$P_{data}(h) = (1/N) \sum_{i \geq h} n(i)$$

where n(i) is equal to the number of positions where the coverage equals i. We define 'N' as the length of the gene or genomic feature:

$$N = \sum_{i \geq 0} n(i) = featurelength$$

For each iteration in the randomised data, the probability for finding height 'h' is calculated as follows:

$$P_{random}(h)(1/N) \sum_{i \geq h} n_{random}(i)$$

After finishing the randomisation steps, it calculates the mean probabilities and standard deviation for finding height 'h' in the randomised data. The FDR is then calculated as the sum of the mean probability and standard deviation for height 'h', divided by the probability of finding the coverage in the actual data:

$$FDR(h) = \frac{\mu_{P_{control}(h)} + \sigma_{P_{control}(h)}}{P_{data}(h)}$$

NOTE. PyCalculateFDRs.py heavily relies numpy arrays to do the calculations and although this dramatically speeds up data processing, the program can consume quite a lot of RAM memory when tackling very long genomic features ($\geq$ 5 million nucleotides). Such long features are generally very rare (only 8 in the human genome), however, when analysing data originating from higher eukaryotes (Human, Mouse, Plant) we would recommend running this script on a machine with **at least 16GB of RAM**. The current version only does 100 iterations by default and will not allow features longer than 5 million nucleotides. You will get slightly better results with 500 iterations or more (limited to 1000), however, this will consume a lot of memory and reduce processing speed.

Figure 5.12: A few lines from a pyCalculateFDRs.py GTF output file.

```
##gff-version 2
# generated by pyCalculateFDRs version 0.0.3, Fri Dec 28 13:01:09 2012
# pyCalculateFDRs.py -f unique_count_output_reads.gtf -o unique_count_output_FDRs.gtf
-m 0.05 --min=5
# chromosome    feature source  start   end     minimal_coverage        strand  .
attributes
chrI    snoRNA  exon    142373  142409  37       +       .       gene_id "snR18";
gene_name "SNR18";
chrI    protein_coding  exon    106370  106413  20      -       .
gene_id "YAL023C"; gene_name "PMT2";
chrI    protein_coding  exon    143888  143950  25      +       .
gene_id "YAL002W"; gene_name "VPS8";
chrI    intergenic_region       exon    65407   65416   10      +       .
gene_id "INT_0_80"; gene_name "INT_0_80";
chrI    CUTs    exon    34540   34595   5       -       .
gene_id "ST3641"; gene_name "CUT438";
```

5.7.1 Input and output files

PyCalculateFDRs requires at least three input files: (1) a pyReadCounters GTF output file, (2) a GTF annotation file and (3) a tab delimited file containing chromosome names and chromosome lengths. More information about this tab delimited file and how to generate it can be found in section 6.1.3.

For each feature in the GTF annotation file it calculates the minimum number of overlapping reads required to obtain an FDR $\leq$ than the set threshold and reports genomic intervals with at least this read coverage in the GTF output file. Figure 5.12 shows a few lines from a pyCalculaterFDRs.py GTF output file. The output file follows the same layout as any other pyCRAC GTF file outputs, except that column 5 shows the lowest read coverage of the region. To generate this output file we used the following command line:

```
pyCalculateFDRs.py -f unique_count_output_reads.gtf -o
    unique_count_output_FDRs.gtf -c chromosomelengths.txt -m 0.05 --min=5
    --gtf=protein_coding_genes.gtf
```

As stated above, the tool also accepts bed6 and GFF formatted input files. To use these file types you need to add the --file_type flag:

```
pyCalculateFDRs.py -f myintervals.bed --file_type=bed -o
    unique_count_output_FDRs.gtf -c chromosomelengths.txt -m 0.05 --min=5
```

Figure 5.13: A few lines from a pyCalculateFDRs.py log file.

```
# generated by pyCalculateFDRs version 0.0.3, Fri Dec 28 19:32:11 2012
# pyCalculateFDRs.py -f unique_count_output_reads.gtf
-o unique_count_output_FDRs_intronless.gtf -m 0.05 --min=5
--gtf=intronless_protein_coding_with_UTR.gtf

####### chromosome chrI #######

# YCR015C
coverage        FDR     actual_density  mean_random_density
>=24    0.0                  4260         0.0
23      0.000708032135397    142          0.01
22      0.00202534068174     149          0.03
21      0.00588089245981     154          0.09
20      0.00786276448056     162          0.16
19      0.0100555677884      163          0.23
18      0.0139914843309      168          0.45
17      0.0242502015449      170          1.13
16      0.0395981737816      170          3.45
15      0.0641036452189      171          7.89
```

```
    --gtf=protein_coding_genes.gtf
pyCalculateFDRs.py -f myintervals.gff --file_type=gff -o
    unique_count_output_FDRs.gtf -c chromosomelengths.txt -m 0.05 --min=5
    --gtf=protein_coding_genes.gtf
```

Here we set the FDR threshold to 0.05 (-m 0.05) and we only analysed regions with a read coverage of at least 5 (--min=5). The output shows genomic intervals with an FDR $\leq$ 0.05.

The tool also generates a log file where for each genomic feature it reports nucleotide densities for specific read coverages and the FDR value. A few lines of a pyCalculateFDRs log file are shown in figure 5.13. The log shows a column with coverage, which indicates the coverage heights found in a genomic feature. The second column shows the FDR for that height. The third column shows the total number of nucleotides that have a coverage of that particular height. The last column shows the average number of nucleotides with a coverage of that height.

5.7.2 Selecting significant clusters using pyCalculateFDRs and bedtools

The bedtools intersect tool can be used with the pyCalculateFDRs.py GTF output file to select reads and clusters overlapping with significant regions. For example:

```
bedtools intersect -s -wa -a count_output_clusters.gtf -b count_output_FDRs_005.gtf > significant_clusters.gtf
bedtools intersect -s -wa -a count_output_reads.gtf -b count_output_FDRs_005.gtf > reads_significant_regions.gtf
```

The -s flag instructs bedtools to only compare intervals that are on the same strand and with the -wa flag the program only outputs those intervals in the count_output_clusters.gtf file that overlap with the FDRs gtf file. One could generate clusters from reads overlapping with significant intervals:

```
bedtools intersect -s -wa -a count_output_reads.gtf -b count_output_FDRs_005.gtf > reads_significant_regions.gtf
pyClusterReads.py -f reads_significant_regions.gtf -o significant_clusters.gtf --co=5 --ch=10
```

NOTE! for pyClusterReads to work properly, the GTF input file has to be sorted by chromosome, then by strand and then by start position. If you are not sure whether your file is correctly sorted, you can run the following sort command line on your pyReadCounters GTF file:

```
sort -k1,1 -k7,7 -k4,4n reads_significant_regions.gtf > sorted_reads_significant_regions.gtf
pyClusterReads.py -f sorted_reads_significant_regions.gtf -o significant_clusters.gtf --co=5 --ch=10
```

The resulting clusters.gtf file could then be used with pyMotif to identify motifs from significant regions. For example:

```
pyMotif.py -f count_output_clusters.gtf -v
```

The reads_significant_regions can also be used with pyPileup and pyReadAligner (see sections 5.4):

```
pyPileup.py -f reads_significant_regions.gtf --file_type=gtf -g genes.list
pyReadAligner.py -f reads_singificant_regions.gtf --file_type=gtf -g genes.list --limit=500
```

5.8 pyCalculateMutationFrequencies

This tool takes three input files: (1) a GTF interval file (pyMotif, pyClusterReads or pyCalculateFDRs output file), (2) a pyReadCounters GTF file and (3) a tab delimited file containing chromosome names and chromosome lengths. More information about this tab delimited file and how to generate it can be found in section 6.1.3.
For each interval in file (1), it calculates mutation frequencies for each nucleotide in the interval. The tool produces a GTF output file with mutation frequencies added as comments.

NOTE! for pyCalculateMutationFrequencies to work properly, the pyReadCounters GTF file has to be sorted by chromosome, then by strand and then by start position. If you modified the file and you are not sure whether your file is correctly sorted, you can run the following sort command line:

```
sort -k1,1 -k7,7 -k4,4n count_output_reads.gtf > sorted_count_output_reads.gtf
```

5.8.1 Command line examples

If you want to add mutation frequencies to your pyCalculateFDRs GTF output file:

```
pyCalculateMutationFrequencies.py -i count_output_FDRs_005.gtf -r
    sorted_count_output_reads.gtf -c chromosomelengths.txt -o
    count_output_FDRs_005_with_mutsfreqs.gtf
```

Or you would like to know mutation frequencies for your enriched motifs:

```
pyCalculateMutationFrequencies.py -i mytopmotifs.gtf -r
    sorted_count_output_reads.gtf -c chromosomelengths.txt -o
    mytopmotifs_with_mutsfreqs.gtf
```

Chapter 6

The pyCRAC scripts

The pyCRAC package contains numerous scripts that can be useful for converting files to different formats or extracting information from output files. All of the scripts have detailed help menus which can be access using the -h or --help flag. This Chapter briefly describes what each script does and provides command line examples.

6.1 Utilities

6.1.1 pyAlignment2Tab.py

The pyAlignment2Tab script in pyCRAC converts the pyReadAligner fasta output format into a tabular format that is easier to read in the terminal and text files.
Usage example:

```
pyAlignment2Tab.py -f sense-PAR_CLIP_RDN18-1.fasta -o 18S_alignment.tab
```

By default, the tool automatically prints to the standard output. To print to a file, use the -o flag. Each line in the tab output file will contain a maximum of 90 characters, making the alignment easier to read. You can change this setting using the --limit flag. When printing to the standard output, each nucleotide will be coloured, which makes it much easier to locate mutations and therefore putative protein cross-linking sites. An example is shown in Figure 6.1. To generate this figure we included the "genes.list" file containing the gene_name *SUP19* in a single column, and then used the command lines listed below.

```
pyReadAligner.py -f SolexaData.novo -g genes.list --blocks --limit=100
    --gtf=yeast.gtf --tab=yeast.tab
pyAlignment2Tab.py -f sense-cDNAs_SolexaData_SUP19_genomic.fasta --limit=90
```

Figure 6.1: PyAlignment2tab can generate colourful tab formatted alignments in the terminal. The example here shows a handful of reads from PAR-CLIP and CRAC data aligned to the yeast *SUP19* tRNA gene. The plot above the alignment shows the corresponding pyPileup result. The gaps in the sequence show deletions, whereas substitutions are indicated in lower case.

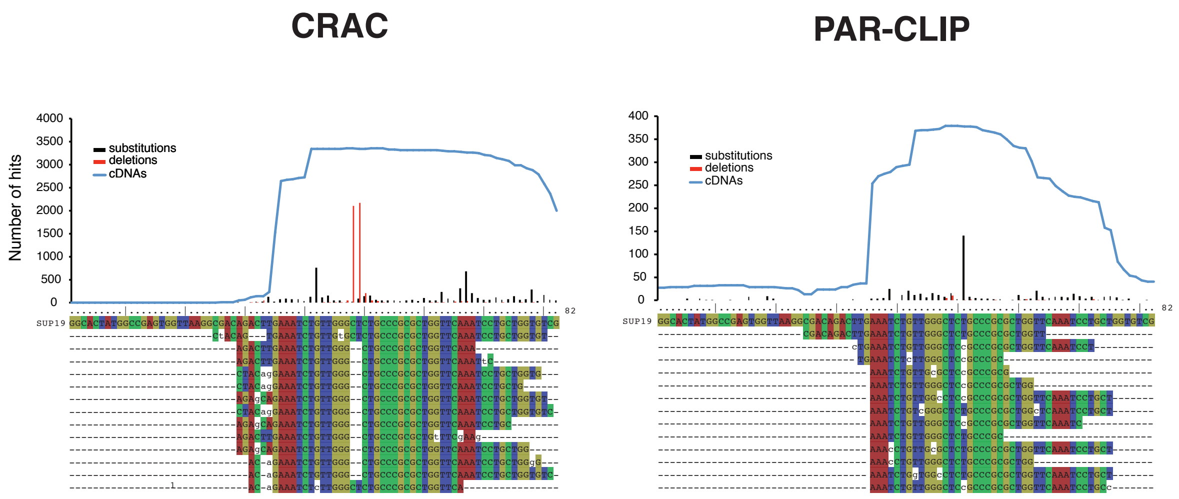

6.1.2 pyFasta2Tab.py

PyFasta2Tab converts fasta formatted files in to the pyCRAC-compatible tabular format.

Example command line:

```
pyFasta2tab.py -f yeastgenome.fasta
```

This will generate the file ”yeastgenome.tab” in the working directory.

6.1.3 pyCalculateChromosomeLengths.py

Some of the pyCRAC tools (pyGTF2BedGraph.py and pyCalculateFDRs.py) require a tab delimited file containing chromosome name and chromosome lengths. We have included the pyCalculateChromosomeLengths.py script to generate this file for you. It requires the genome sequence in fasta or tab format. The .length output file is structured as shown in figure 6.2.

The script generates a file with a .length extension. A command line example:

Figure 6.2: An example of a pyCalculateChromosomeLenghts.py output file.

```
2-micron    6318
Mito    85779
chrI    230208
chrII   813178
chrIII  316617
chrIV   1531919
chrIX   439885
chrV    576869
chrVI   270148
chrVII  1090947
chrVIII 562643
chrX    745741
chrXI   666454
chrXII  1078175
chrXIII 924429
chrXIV  784334
chrXV   1091289
chrXVI  948062
```

```
pyCalculateChromosomeLengths.py -f
    /usr/local/pyCRAC/db/Saccharomyces_cerevisiae.EF2.59.1.0.fa.tab --file_type=tab
```

6.2 Processing fastq and fasta formatted data

6.2.1 Removing PCR duplicates by collapsing the data

PCR over-amplification can be a problem with CLIP/CRAC experiments and can give a false impression of the actual number of reads mapped to genomic features. These duplicate reads can be removed using the fastx_collapser (`http://hannonlab.cshl.edu/fastx_toolkit/`) or the pyCRAC pyFastqDuplicateRemover tool. Both programs function by collapsing identical sequences into one.
Usage example:

```
fastx_collapser -i rawdata.fastq -o collapseddata.fasta
```

At the time of writing this manual, fastx_collapser could not handle paired end data. If you want to process paired-end data using fastx_collapser you will need to merge the two raw data files first. For this purpose we included the pyFastqJoiner.py script in pyCRAC.

Usage example:

```
pyFastqJoiner -f rawdata_1.fastq rawdata_2.fastq -o merged_rawdata
```

By default pyFastqJoiner writes to the standard output and the output can therefore be piped directly to fastx_collapser:

```
pyFastqJoiner -f rawdata_1.fastq rawdata_2.fastq | fastx_collapser >
    merged_rawdata.fasta
```

To split the data again we have included the pyFastqSplitter.py tool in pyCRAC, which can read from the standard input:

```
pyFastqJoiner -f rawdata_1.fastq rawdata_2.fastq | fastx_collapser |
    pyFastqSplitter.py --file_type=fasta --stdin -o collapsed_rawdata
```

NOTE that you should only provide one output file name for pyFastqSplitter.py. The pyFastqJoiner tool can also handle gzip-compressed files and compress output files:

```
pyFastqJoiner --filetype=fastq.gz -f rawdata_1.fastq.gz rawdata_2.fastq.gz |
    fastx_collapser | pyFastqSplitter.py --file_type=fasta --stdin -o
    collapsed_rawdata --gzip
```

The main reason we wrote our own joiner and splitter scripts is because existing tools required sequences from the forward and reverse reaction to have the same length. Hence, it is not possible to do any sequence pre-processing or trimming steps, such as removing adapter sequences, prior to collapsing your data. pyFastqJoiner includes a -c flag that allows you to insert a character between the two sequences. For example, adding the -c ":" inserts two hashes between the joined sequences. Setting the same flag in pyFastqSplitter will tell the tool where to split the data.
Example:

```
pyFastqJoiner --filetype=fastq.gz -f rawdata_1.fastq.gz rawdata_2.fastq.gz -c ":"
    | do something else | pyFastqSplitter.py -c ":" --file_type=fasta --stdin -o
    collapsed_rawdata
```

This does, however, cause problems with the fastx_collapser tool, which will complain that there are illegal characters in the DNA sequence. PyFastqDuplicateRemover ignores these characters:

```
pyFastqJoiner --filetype=fastq.gz -f rawdata_1.fastq.gz rawdata_2.fastq.gz -c ":"
    | pyFastqDuplicateRemover.py | pyFastqSplitter.py -c ":" --file_type=fasta
    --stdin -o collapsed_rawdata
```

6.2.2 Removing PCR duplicates using random nucleotide information

We routinely include three to six random nucleotides in the 5' adapter sequence. During demultiplexing, pyBarcodeFilter will attach the two hashes and random barcode sequence to the read header. The pyFastqDuplicateRemover script will look at both the sequence and look for random nucleotide sequences in the header (see Figure ??). The pyFastqDuplicateRemover tool has the added advantage that it can process paired-end fastq and fasta data directly without having to join and split the sequences.

pyFastqDuplicateRemover usage examples:
Single-end data:

```
pyFastqDuplicateRemover.py -f rawdata.fastq -o collapseddata.fasta
```

Paired-end data:

```
pyFastqDuplicateRemover.py -f rawdata_1.fastq -r rawdata_2.fastq -o collapseddata
```

NOTE When processing paired-end data you should not add a file extension to the name of the output file.

The tool looks for two hashes near the end of the header (blue) and assumes that the sequence following these hashes (red) is the random barcode sequence. If it encounters two identical (paired-) end sequences with the same random barcode sequences, it will collapse them into one sequence. The orange characters indicate that this header originates from the forward sequencing reaction. The green characters indicate an Illumina indexing sequence.

Although pyFastqDuplicateRemover can process paired-end data, in some cases you might want to do multiple processing steps on your data in a stream. In this case you

Figure 6.3: pyFastqDuplicateRemover scans the header for the presence of two hashes near the end (blue) and assumes that the sequence following these hashes (red) is the random barcode sequence. If it encounters two identical (paired-) sequences with the same random barcode sequences, it assumes they are PCR duplicates and collapse them into one sequence. The orange characters indicate that this header originates from the forward sequencing reaction. The green characters indicate an Illumina indexing sequence.

```
FCC0TU2ACXX:4:1101:1968:2135#ACAGTGAT1##GTTCTC
```

will need to join the sequences first, as described in previous examples. In the following command lines we first split the data based on the random barcode sequence using pyBarcodeFilter. We then joined the forward and reverse reads together. However, because the sequence in the forward read had the barcode removed, it is shorter than the reverse read sequence. Hence we need to add a character using the -c flag between the two sequences so that pyFastqSplitter later on knows where the two sequences were joined. **NOTE!** pyFastqDuplicateRemover produces a fasta output file so the --file_type=fasta flag must be added to the pyFastqSplitter command line.

```
pyBarcodeFilter.py -f rawdata_1.fastq -r rawdata_2.fastq -b barcodes.txt -m 1
```

This produced the rawdata_1_NNNATGC.fastq and rawdata_2_NNNATGC.fastq output files. After joining these files, you can essentially do many processing steps in a single stream, as shown below:

```
pyFastqJoiner.py -f rawdata_1_NNNATGC.fastq rawdata_2_NNNATGC.fastq -c ":" |
    pyFastqDuplicateRemover.py | do many more things | pyFastqSplitter.py
    --file_type=fasta -c ":" -o collapsed_rawdata
```

This command line produced the collapsed_rawdata_1.fasta and collapsed_rawdata_2.fasta files.

Figure 6.4 shows examples of how the data is processed during each step.

6.3 GTF file manipulation tools

6.3.1 pyCheckGTFfile.py

The GTF2 parser included in pyCRAC primarily uses the 'gene_name' to indicate feature names. If no gene_name entries exist, it will look for 'gene_id' entries. One of the

Figure 6.4: Example showing what happens to the data during pyBarcodeFilter and pyFastqDuplicateRemover processing steps. The random barcode sequence is indicated in red, where as the barcode for the experiment is indicated in blue. If the barcode list file contains random nucleotide (see Table 5.1) then the pyBarcodeFilter tool will attach two hashes followed by the random barcode sequence to the header and remove the barcode from the sequence. The pyFastqDuplicateRemover tool then collapses the data and converts the fastq entry into the fasta format and included the random nucleotide sequence (red) and the number of identical sequences it found in the raw data (orange)

Unprocessed fastq data:

```
@FCC102EACXX:3:1101:3231:2110#TGACCAAT/1
GCGCCTGCCAATTCCATCGTAATGATTAATAGGGACGGTCGGGGGCATC
+
bb_ceeeeggggggiiiiiifghiihiihiiiiiiiiiifggfhiecccc
```

After pyBarcodeFilter:

```
@FCC102EACXX:3:1101:3231:2110#TGACCAAT/1##GCGCCT
TCCATCGTAATGATTAATAGGGACGGTCGGGGGCATC
+
giiiiiifghiihiihiiiiiiiiiifggfhiecccc
```

```
This entry is printed to the NNNNNNGCCAAT barcode file
```

After pyFastqDuplicateRemover:

```
>1_GCGCCT_5
TCCATCGTAATGATTAATAGGGACGGTCGGGGGCATC
```

biggest problems with GTF annotation files from ENSEMBL is that they sometimes contain duplicated gene_name entries. This will seriously confuse the pyCRAC tools and cause errors. Therefore it is **ESSENTIAL** that you run the pyCheckGTFfile.py script before you use the pyCRAC tools. It will rename duplicate gene_name entries into the corresponding transcript_name or gene_id and printed to the standard output. As an alternative approach, one can modify the GTF file by ensuring that the gene_name entries in the GTF file are a fusion of gene_id and gene_name. This immediately removes duplicate gene_name entries.
Usage example:

```
pyCheckGTF.py --gtf=Homo_sapiens.GRCh37.69.gtf -o
    Homo_sapiens.GRCh37.69_corrected.gtf
```

6.3.2 pyExtractLinesFromGTF.py

Sometimes you might be interested in looking at hits for handful of genes and you do not want the program to load the entire GTF file into memory. You may also be interested in grabbing your favourite genes from a pyMotif or pyReadCounters GTF output file. To simplify this, we included pyExtractLinesFromGTF.py, an homage to ”grep”. This script takes a long list of gene or transcript names and then reads through a GTF file and outputs (”greps”) all the lines in the GTF file that include names in the list. These gene or transcript names should be supplied in a text file in a single column. The script automatically prints to the terminal standard output. By default pyExtractLinesFromGTF.py assumes that the data is coming from the standard input.
Usage example:

```
pyExtractLinesFromGTF.py -g genes_list.txt --gtf=Yeast.gtf > new.gtf
```

6.3.3 pyGTF2bed

pyGTF2bed can be used to convert GTF files, including all pyCRAC GTF output files to the bed6 format.
NOTE! The GTF input file has to be sorted by chromosome, then by strand and then by start position. The pyReadCounters tool sorts the read intervals for you so if you did not modify the file in any way it should work without any problems. If you are not sure whether your file is correctly sorted, you can run the following sort command line on your

pyReadCounters GTF file:

```
sort -k1,1 -k7,7 -k4,4n reads.gtf > reads_sorted.gtf
```

A few command line examples:

```
pyGTF2bed.py --gtf=data_count_reads.gtf -o data_count_reads.bed
```

To visualise the data in the UCSC genome browser, we often convert the GTF output files to bed6 files to reduce the file size. This script also allows you can use add a name for the experiment and a description using the -n and -d flags, respectively. In addition to change a color for the track and the strand we included the -c and -s flags.

```
pyGTF2bed.py --gtf=data_count_reads.gtf -o data_count_reads.bed -n my_data -d
    my_data
```

Adding -c red to the command line instructs the UCSC genome browser to color the track red:

```
pyGTF2bed.py --gtf=data_count_reads.gtf -o data_count_reads.bed -n my_data -d
    my_data -c red
```

The following example shows the usage of the -s flag. Here the "+" strand will be coloured green and the "-" strand cyan.

```
pyGTF2bed.py --gtf=data_count_reads.gtf -o data_count_reads.bed -n my_data -d
    my_data -c red -s 'green,cyan'
```

NOTE!!. To change the color of the strands the colours need to be in quotes, exactly as shown in the example. If the script does not recognise the colours you entered it will display a list of colours that you can choose from.

6.3.4 pyGTF2bedGraph

This script is an homage to the bedtools genomecoverage script. It is certainly not as fast as bedtools genomecoverage but has the advantage that it can generate bedgraph files

for substitutions and deletions from the comments in pyReadCounters GTF files (For example: # 4562D;). It also generates bedgraph files for both strands at the same time. **NOTE!** The GTF input file has to be sorted by chromosome. The pyReadCounters tool sorts the read intervals for you so if you did not modify the file in any way it should work without any problems. If you are not sure whether your file is correctly sorted, you can run the following sort command line on your pyReadCounters GTF file:

```
sort -k1,1 -k7,7 -k4,4n reads.gtf > reads_sorted.gtf
```

The script requires at least two inputs: a GTF file with read intervals and a tab-delimited file containing information about the lengths of each chromosome. To generate the second file, you can use the pyCalculateChromosomeLengths.py script (see section 6.1.3). A few command line examples:

```
pyGTF2bedGraph.py --gtf=data_count_output_reads.gtf -o mydata_intervals -c
    chromosomelengths.txt
```

This generates bedgraph files for the read intervals specified in the data_count_output_reads.gtf file. By default, the script assumes that each interval is unique and ignores the number of identical reads reported by pyReadCounters (in the 'score' column, see section 4.4.2). If you want to generate bedgraph files containing hits from all reads, you need to include the --count flag:

```
pyGTF2bedGraph.py --gtf=data_count_output_reads.gtf -o mydata_intervals -c
    chromosomelengths.txt --count
```

To make bedgraph files for substitutions:

```
pyGTF2bedGraph.py --gtf=data_count_output_reads.gtf -o mydata_substitutions -c
    chromosomelengths.txt -t substitutions
```

For deletions:

```
pyGTF2bedGraph.py --gtf=data_count_output_reads.gtf -o mydata_deletions -c
    chromosomelengths.txt -t deletions
```

This GTF conversion script can also report counts for 5' end and 3' ends of intervals [**NOTE!**: –iCLIP flag has been removed since version 1.2.2.2]. Examples:

```
pyGTF2bedGraph.py --gtf=data_count_output_reads.gtf -t startpositions -o
    mydata_startpositions -c chromosomelengths.txt
pyGTF2bedGraph.py --gtf=data_count_output_reads.gtf -t endpositions -o
    mydata_endpositions -c chromosomelengths.txt
```

NEW since version 1.2.2.2:
If you want to normalize your data to hits per million, use the --permillion flag. This will ONLY work if the GTF interval file contains information about the total number of mapped reads, so do not delete the lines starting with a hash!.

```
pyGTF2bedGraph.py --gtf=data_count_output_reads.gtf -o
    mydata_intervals_normalized -c chromosomelengths.txt --count --permillion
pyGTF2bedGraph.py --gtf=data_count_output_reads.gtf -o
    mydata_startpositions_normalized -t startpositions -c chromosomelengths.txt
    --count --permillion
```

6.3.5 pyGTF2sgr.py

This tool produces sgr files from cluster, motif or interval GTF files. It is similar to bedtools genomecov but we have added a few features to make it also possible to extract mutation information from pyReadCounters or pyClusterReads GTF output files. It produces output files for the + and - strand simultaneously. The sgr output files can be loaded into the genome browsers to visualise read distribution of the intervals over the genome. It is also quite easy to extract hits for individual features from these files. The script requires at least two inputs: a GTF file with intervals and a tab-delimited file containing information about the length of each chromosome. To generate the latter, you can used the pyCalculateChromosomeLenghts.py script included in the pyCRAC package (see section 6.1.3). Most of the flags that can be used in pyGTF2sgr are also present in the pyGTF2bedGraph.py script (see section 6.3.4)

Usage examples:

```
pyGTF2sgr.py --gtf=data_count_output_reads.gtf -c chromosomelengths.txt -o
    mydata_intervals
```

This produces an intervals_plus_strand.sgr and an intervals_minus_strand.sgr file. Note that chromosomal positions without any coverage are not reported in these output files. To include these you need to add the --zeros flag:

```
pyGTF2sgr.py --gtf=data_count_output_reads.gtf -c chromosomelengths.txt -o
    mydata_intervals --zeros
```

By default, the script assumes that each interval is unique and ignores the number of identical reads reported by pyReadCounters (in the score column, see section 4.4.2). If you want to generate an sgr file from all the reads, including duplicates, you need to include the --count flag:

```
pyGTF2sgr.py --gtf=data_count_output_reads.gtf -c chromosomelengths.txt -o
    mydata_intervals --zeros --count
```

If you only want to include positions that have a certain read coverage, lets say 4000 reads, then you can filter the data using the --min flag. All of the positions with a read coverage less than the set minimum will be set to zero. The following command line will only report those positions with a coverage of 4000 or higher:

```
pyGTF2sgr.py --gtf=data_count_output_reads.gtf -c chromosomelengths.txt -o
    mydata_intervals_min_4000 --count --min=4000
```

This GTF conversion script can also report counts for 5' end and 3' ends of intervals [**NOTE!**: –iCLIP flag has been removed since version 1.2.2.2]. Examples:

```
pyGTF2sgr.py --gtf=data_count_output_reads.gtf --type startpositions -o
    mydata_startpositions -c chromosomelengths.txt --count
pyGTF2sgr.py --gtf=data_count_output_reads.gtf --type endpositions -o
    mydata_endpositions -c chromosomelengths.txt --count
```

NEW since version 1.2.2.2:
If you want to normalize your data to hits per million, use the --permillion flag. This

will ONLY work if the GTF interval file contains information about the total number of mapped reads, so do not delete the lines starting with a hash!.

```
pyGTF2sgr.py --gtf=data_count_output_reads.gtf -o mydata_intervals_normalized -c
    chromosomelengths.txt --count --permillion
pyGTF2sgr.py --gtf=data_count_output_reads.gtf -o
    mydata_startpositions_normalized -t startpositions -c chromosomelengths.txt
    --count --permillion
```

6.3.6 pyGetGTFSources.py

The pyGetGTFSources script extracts the source names or annotations from GTF files (column 2, see Table 4.2). The following command line extracts the source names from the default yeast GTF feature file:

```
pyGetGTFSources.py
    --gtf=/usr/local/pyCRAC/db/Saccharomyces_cerevisiae.EF2.59.1.3.gtf
```

This should produce the following output:

```
# pyGetGTFSources.py --gtf==Saccharomyces_cerevisiae.EF2.59.1.3.gtf
# Thu Jul 21 18:07:17 2011
# source list generated from: /usr/local/pyCRAC/db/
Saccharomyces_cerevisiae.EF2.59.1.3.gtf
rRNA
intergenic_region
SUTs
tRNA
protein_coding
pseudogene
CUTs
snoRNA
ncRNA
snRNA
```

The script can also count the occurrence of each source or annotation in GTF files. Fore example:

```
pyGetGTFSources.py
    --gtf=/usr/local/pyCRAC/db/Saccharomyces_cerevisiae.EF2.59.1.3.gtf --count
```

This should produce the following output:

```
# pyGetGTFSources.py --gtf=Saccharomyces_cerevisiae.EF2.59.1.3.gtf --count
# Sat Dec 15 11:23:04 2012
# source list generated from:
/usr/local/pyCRAC/db/Saccharomyces_cerevisiae.EF2.59.1.3.gtf
pseudogene        23
snRNA             6
intergenic_region 6884
SUTs              847
tRNA              359
protein_coding    27491
rRNA              29
CUTs              925
snoRNA            79
ncRNA             15
```

6.3.7 pyGetGeneNamesFromGTF.py

The pyGetGeneNames.py scripts extracts gene or transcript names from a GTF file. By default the list will be printed to the standard output in the terminal. Use the -h flag to get a help menu.

Usage examples:

```
pyGetGeneNamesFromGTF.py --gtf=SolexaDataCTTG.gtf --attribute=gene_name >
    CTTG_gene_names.txt
```

What if we are only interested in gene_ids?

```
pyGetGeneNamesFromGTF.py --gtf=SolexaDataCTTG.gtf --attribute=gene_id >
    CTTG_gene_ids.txt
```

You can also use the --count flag with this script to count the occurrences of each gene or transcript name:

```
pyGetGeneNamesFromGTF.py --gtf=SolexaDataCTTG.gtf --attribute=gene_name --count >
    CTTG_gene_names.txt
```

6.3.8 pySelectMotifsFromGTF.py

We included pySelectMotifsFromGTF.py to make it easy for users to extract k-mers containing a particular sequence from a pyMotif GTF file. As always, use the -h or --help flag with any pyCRAC program to get a help menu. This script requires the name of the pyMotif GTF file (--gtf flag), a sequence string (-m or --motif flag) and a name of a GTF output file (-o flag).

In the following example we use the script to extract any k-mer containing the CTTG sequence from a pyMotif GTF file that have a Z-score of 5 or higher.

```
pySelectMotifsFromGTF.py --gtf=SolexaDataCTTG.gtf -m CTTG -z 5.0 -o
    SolexaDataCTTG.gtf
```

Note that you can include degenerate nucleotides in your motif search string:

```
N = A, G, C or T
R = A or G = puRine
Y = C or T = pYrimidine
M = A or C = aroMatic
S = G or C
W = A or T
K = G or T = Keto
V = A, C or G = Not T (letter after)
D = A, G or T = Not C
H = A, C or T = Not G
B = C, G or T = Not A
```

So if you enter KBCTTG as search string and length=6, then the program will extract a large number of six-mers from your data. If you set length = 8, it will look for this pattern in a stretch of 8 nucleotides.
Example:

```
pySelectMotifsFromGTF.py --gtf=SolexaDataCTTG.gtf -m KBCTTG -l 8 -z 5.0 -o
    SolexaDataKBCTTG.gtf
```

If you do not include an output file name, the results will be printed to the standard

output in the terminal. This allows you to pipe the output to other tools. The GTF output file can be viewed in genome browsers (such as UCSC or IGB). If you want to analyse the distribution of top k-mers on genomic features with a particular annotation (for example all protein coding genes), you can use the pyBinCollector program (see section 5.6). The SolexaDataCTTG.gtf file also contains gene_name and transcript_name attributes. To obtain a list of all the genes with CTTG motifs, one can use the pyGetGeneNamesFromGTF.py script.

6.3.9 pyNormalizeIntervalLengths.py

pyCalculateFDRs.py or pyClusterReads.py sometimes report 10-15 nucleotide intervals in GTF output files. These may be too short to motif searches with pyMotif or MEME or perhaps RNAfold if you want to look at secondary structures of selected intervals. This tool allows you to increase the length of your intervals to make these analyses possible. With pyNormalizeIntervalLengths.py you can set (1) a fixed length for each interval or (2) you can set a minimum length for each feature. The script requires at least two inputs: a GTF/bed6/GFF file with intervals and a tab-delimited file containing information about the length of each chromosome. To generate the latter, you can used the pyCalculateChromosomeLenghts.py script included in the pyCRAC package (see section 6.1.3).

Usage examples:

```
pyNormalizeIntervalLengths.py -f myintervals.gtf --min=30 -o
    myintervals_min30.gtf -c chromosomelengths.txt
```

The resulting output file will contain intervals with a minimum length of 30 nucleotides.

```
pyNormalizeIntervalLengths.py -f myintervals.gtf -l 30 -o myintervals_all30.gtf
    -c chromosomelengths.txt
```

In this case all the intervals in the resulting output file will be 30 nucleotides in length.
